# Social, psychosocial, and lifestyle determinants of diabetes and prediabetes in US adults before and after COVID-19: a cross-sectional NHANES analysis

**DOI:** 10.1186/s13098-026-02100-8

**Published:** 2026-01-31

**Authors:** Yuan Zhao, Dongyu Hu, Jiacheng Cheng, Huili Cao, Xiaojuan Wang, Junhua He, Yikun Zhu, Jin Li

**Affiliations:** 1 Department of Endocrinology and Metabolism, The Second Hospital of Shanxi Medical University, Shanxi Medical University, 382 Wuyi Road, Xinghualing, Taiyuan, 030001 China; 2 Department of Cardiology, The Second Hospital of Shanxi Medical University, Shanxi Medical University, Taiyuan, China

**Keywords:** NHANES, COVID-19, Diabetes, Prediabetes, Social determinants, Psychosocial factors, Lifestyle behaviors, Risk behaviors, Sex differences, Interaction analysis, Cross-sectional study, Population-based study

## Abstract

**Background:**

Type 2 diabetes and prediabetes are increasingly shaped by social, psychological, and lifestyle determinants beyond traditional biomedical risk factors. The COVID-19 pandemic profoundly disrupted these determinants. We aimed to assess, using a comparative cross-sectional analysis of nationally representative National Health and Nutrition Examination Survey (NHANES) data, how social, psychosocial, and behavioral determinants of dysglycemia in US adults shifted before and after the COVID-19 pandemic, accounting for differences by sex, age, and disease stage, as well as statistical interactions between these factors and the pandemic period.

**Methods:**

We conducted a comparative cross-sectional analysis of nationally representative data from the NHANES, comparing pre-pandemic (2017–March 2020, n = 4,946) and post-pandemic (2021–2023, n = 2,820) periods, applying NHANES sampling weights to ensure population representativeness. Multivariable logistic regression examined associations between sociodemographic factors, depressive symptoms, lifestyle behaviors, and glycemic status. To explore potential pandemic-related effect modifications, we performed sex- and age-stratified analyzes and formally tested for interactions by including interaction terms between these determinants and the pandemic period in the models.

**Results:**

The age-adjusted prevalence of type 2 diabetes significantly decreased from 13.9% to 11.3% after the pandemic (P = 0.001), while the prevalence of prediabetes remained stable. Higher education consistently and significantly reduced the risk of type 2 diabetes (Pre-pandemic: OR 0.49,95%CI:0.33–0.73, P = 0.002; Pandemic: OR 0.44, 95% CI: 0.29–0.65, P = 0.004). Income effects varied by sex—protective for women (OR 0.43, 95% CI: 0.27–0.71, P = 0.010) but associated with a higher risk of prediabetes in men (OR 1.54, 95% CI: 1.13–2.08, P = 0.024). Depression showed stage-specific moderation: it significantly mitigated the risk-enhancing effects of behavioral factors on prediabetes (smoking, ROR 0.36, 95% CI: 0.16–0.83, P = 0.025; alcohol, ROR 0.75, 95% CI: 0.57–0.99, P = 0.048), but amplified these risk effects in type 2 diabetes (smoking, ROR 3.17, 95% CI: 1.32–7.61, P = 0.017; alcohol, ROR 4.79, 95% CI: 1.96–11.71, P = 0.002). The protective effect of high physical activity against prediabetes in young adults (20–39 years) observed pre-pandemic was attenuated post-pandemic. Longer sleep duration significantly protected men from prediabetes before the pandemic (OR 0.54, 95% CI: 0.37–0.79, P = 0.005), with no effect observed in women (OR 0.98, 95% CI: 0.68–1.40). Dietary inflammatory potential (DII) emerged as a significant risk factor for type 2 diabetes among young adults during the pandemic (OR 2.10, 95% CI: 1.36–3.26, P = 0.009).

**Conclusions:**

The COVID-19 pandemic coincided with changes in risk patterns for prediabetes and type 2 diabetes that were highly specific to sex, age, and disease stage. These findings underscore the importance of incorporating psychosocial and behavioral determinants into diabetes surveillance and prevention strategies in the post-pandemic era.

**Supplementary Information:**

The online version contains supplementary material available at 10.1186/s13098-026-02100-8.

## Introduction

Type 2 diabetes is a major global health concern, affecting an estimated 462 million adults worldwide and contributing significantly to mortality and healthcare costs [1]. While type 2 diabetes is well recognized to be associated with established biomedical risk factors such as excess adiposity, metabolic derangements, and genetic predisposition [2–4], these factors alone inadequately explain the heterogeneity of disease risk observed across populations [5]. As a result, increasing attention has been given to broader social, psychological, and lifestyle determinants of health, which have profound and often interdependent effects on metabolic health [6].

Social determinants—including the conditions in which people are born, live, and work—influence healthcare access and opportunities for healthy living. Lower socioeconomic status (SES) is consistently associated with higher diabetes risk and poorer glycemic control [7, 8]. Psychological factors, such as depressive symptoms and chronic stress, contribute to metabolic dysfunction through dysregulation of the hypothalamic–pituitary–adrenal axis, increased systemic inflammation, and impaired insulin sensitivity [9, 10]. Lifestyle behaviors, including unhealthy dietary patterns, physical inactivity, smoking, and alcohol consumption, significantly increase the risk of metabolic syndrome, with additive effects when multiple lifestyle risk factors coexist [11]. Importantly, these domains do not operate in isolation; rather, they are interdependent. Structural social conditions shape psychosocial burdens, which, in turn, influence behavioral patterns that directly affect glucose metabolism [12]. This interconnected, layered framework—linking social context, psychosocial stressors, and behavioral risk factors—offers an integrated conceptual model for understanding dysglycemia risk, particularly in the context of societal disruption. Prior research also indicates that physiological stress responses, mental health burdens, and behavioral adaptations vary significantly by sex and age [13, 14], highlighting the importance of demographic stratification and interaction-based analyzes.

The COVID-19 pandemic served as a profound societal disruptor, affecting multiple aspects of life. Widespread lockdowns, economic uncertainty, and disruptions to healthcare systems significantly hindered the ability to conduct routine screenings and manage chronic conditions [15]. Additionally, the pandemic led to significant shifts in daily behaviors, such as eating habits, sleep patterns, alcohol consumption, and physical activity [16]. At the same time, the pandemic heightened psychosocial stressors, such as social isolation, unemployment, and increasing rates of depression, all of which negatively impacted diabetes self-care and metabolic health [17].

Although some studies have documented pandemic-related changes in behaviors, mental health, and glycemic control among individuals with diabetes or COVID-19 survivors, these are primarily based on clinical populations or retrospective recall, lacking a pre-pandemic baseline at the population level [18, 19]. To date, no study has simultaneously examined changes in the social, psychosocial, and behavioral determinants of dysglycemia using nationally representative pre- and post-pandemic data. The United States offers a critical context for this investigation, given its unique combination of widespread COVID infection, varying public health mandates, and significant socioeconomic disruption. The National Health and Nutrition Examination Survey (NHANES) provides the most comprehensive, nationally representative data on metabolic and behavioral health in the US, enabling direct comparisons of pre- and post-pandemic periods using harmonized measures. Utilizing this resource, our study adopts a comparative cross-sectional design to analyze shifts in the social, psychosocial, and lifestyle determinants of dysglycemia among US adults before and after the COVID-19 pandemic, based on NHANES data. We further conduct sex- and age-stratified analyzes and formally test for statistical interactions with the pandemic period to identify subgroup-specific vulnerabilities and to elucidate evolving risk pathways.

## Methods

### Study population

This study employed a comparative cross-sectional design using two nationally representative samples from the NHANES: one representing the pre-pandemic period (2017–March 2020) and the other representing the post-pandemic period (2021–2023). The pre-pandemic sample was constructed by merging the 2017–2018 and 2019–March 2020 cycles, in accordance with National Center for Health Statistics (NCHS) guidelines. The 2019–March 2020 cycle was suspended early due to the pandemic and is not nationally representative on its own. By combining it with the preceding complete cycle, we restore representativeness for the immediate pre-pandemic era. Starting from 2017 ensures temporal proximity to the pandemic and consistency in key measurement protocols. The 2021–2023 cycle was selected as the post-pandemic period because it is the first completed NHANES survey conducted entirely after the acute phase of the pandemic, reflecting a stabilized societal context.

The initial pool included 22,499 participants across both periods. To construct our analytical sample, we applied a series of exclusion criteria. First, we excluded individuals under the age of 20 (n = 7,906) and those who were pregnant at the time of examination (n = 130), as these groups have distinct metabolic and physiological characteristics. Second, to ensure a complete-case analysis, we excluded participants with missing data on any key variable central to our research question. These key variables included: 1) glycemic status (defined by fasting plasma glucose [FPG] or hemoglobin A1c [HbA1c]); 2) core sociodemographic factors (age, sex, race/ethnicity, education, family income-to-poverty ratio [PIR], marital status, employment, health insurance, and nativity); 3) psychosocial measures (Patient Health Questionnaire-9 [PHQ-9] score for depressive symptoms); and 4) lifestyle behaviors (smoking status, alcohol consumption, physical activity level, sleep duration, and sedentary time). A total of 6,780 participants were excluded due to missing data on one or more of these variables. The specific diagnostic thresholds, measurement methods, and categorization schemes for all key variables are described in detail in the following ‘Clinical and Laboratory Evaluations’ subsection. No further clinical or epidemiological exclusion criteria were applied. Following these exclusions, the final analytic sample consisted of 7,766 participants (pre-pandemic, 2017–March 2020: n = 4,768; post-pandemic, 2021–2023: n = 2,988). A flowchart outlining participant selection is provided in Supplementary Fig. 1. The NHANES protocol was approved by the NCHS Research Ethics Review Board, and all participants provided written informed consent.

### Clinical and laboratory evaluations

#### Sociodemographic factors

Sociodemographic variables included age (years), sex (men, women), race/ethnicity (non-Hispanic White, non-Hispanic Black, Mexican American, other Hispanic, non-Hispanic Asian, other races), educational attainment (less than college, some college, college graduate or above), family poverty-income ratio (PIR; categorized as low: < 1.3, middle: 1.3–3.5, high: ≥ 3.5), marital status (never married, divorced/separated/widowed, married/living with partner), employment status (not employed, part-time employee, full-time employee), health insurance coverage (yes, no), and place of birth (US-born, foreign-born).

#### Psychological factors

Depressive symptoms were assessed using the PHQ-9. A summary score ≥ 10 was used to define the presence of moderate-to-severe depressive symptoms, consistent with established clinical thresholds [20].

#### Lifestyle and behavioral factors

Smoking status was categorized as never, former, or current smoker. Alcohol consumption was calculated as grams per week and analyzed as a continuous variable. Sleep duration was derived from self-reported typical sleep hours on weekdays and weekends, and was categorized as short (< 6 h), optimal (6–8 h), or long (≥ 8 h). Physical activity level was calculated from self-reported frequency and duration of moderate and vigorous leisure-time activities over the past 7 days. Total minutes per week were computed and categorized according to WHO 2020 guidelines as low (< 150 min/week), moderate (150–300 min/week), or high (≥ 300 min/week) [21]. Sedentary time was assessed as the self-reported number of hours spent sitting per day.

#### Dietary assessment

Dietary intake was assessed using a 24-h dietary recall. Nutrient intakes, including total energy, carbohydrates, sugars, dietary fiber, proteins, fats (saturated, polyunsaturated), cholesterol, specific fatty acids, vitamins, and minerals, were analyzed. Two composite dietary indices were calculated: the Dietary Inflammatory Index (DII) was constructed using the standardized scoring algorithm developed by Shivappa et al., which quantifies the inflammatory potential of an individual’s diet [22]. For each participant, intake of 27 food parameters from NHANES 24-h recalls (e.g., energy, macronutrients, vitamins, minerals, flavonoids) was converted to Z-scores using the global comparative database. These Z-scores were then transformed into centered percentiles and multiplied by literature-derived inflammatory effect scores for each parameter. The component scores were summed to calculate the overall DII, with higher values indicating more pro-inflammatory dietary patterns. The Composite Dietary Antioxidant Index (CDAI) was calculated using six antioxidant nutrients available in NHANES (vitamins A, C, and E, selenium, zinc, and manganese) [23]. For each nutrient, intake was standardized to Z-scores based on sample means and SDs, with positive Z-scores indicating higher antioxidant exposure. The CDAI was obtained by summing the standardized scores, following established methodology. Higher CDAI values reflect greater overall antioxidant capacity in the diet. Since neither DII nor CDAI has established clinical cut-off points, both indices were analyzed as continuous variables in the primary models.

#### Clinical measurements and morbidities

Clinical measurements included weight, height, waist circumference (WC), systolic and diastolic blood pressure, FPG, HbA1c, total cholesterol (TC), and high-density lipoprotein (HDL) cholesterol. Diabetes was defined as self-reported physician diagnosis, current use of glucose-lowering medication, FPG ≥ 126 mg/dL, or HbA1c ≥ 6.5%. Prediabetes was defined as FPG 100–125 mg/dL or HbA1c 5.7–6.4%. Normoglycemia was defined as HbA1c < 5.7% and FPG < 100 mg/dL without diagnosed diabetes [24]. Obesity was defined as a body mass index (BMI) ≥ 30 kg/m^2^, calculated as weight in kilograms divided by height in meters squared. Hypertension was defined as a self-reported physician diagnosis, current use of antihypertensive medication, or an average systolic blood pressure ≥ 140 mmHg or diastolic blood pressure ≥ 90 mmHg based on three consecutive blood pressure measurements [25]. Multimorbidity is defined as the co-occurrence of two or more chronic conditions. These chronic conditions include arthritis, cancer, cardiovascular diseases (such as congestive heart failure, coronary heart disease, myocardial infarction, stroke, or angina), hypertension, pulmonary diseases (including chronic obstructive pulmonary disease, emphysema, chronic bronchitis, or asthma), and chronic kidney disease (CKD) [26]. CKD is defined according to the 2021 Kidney Disease: Improving Global Outcomes (KDIGO) Clinical Practice Guideline as an estimated glomerular filtration rate (eGFR) of less than 60 mL/min/1.73 m^2^ [27].

### Statistical analysis

To ensure comparability between periods, we harmonized all variable definitions and applied identical data processing procedures. All analyzes accounted for the complex, multi-stage sampling design of NHANES by incorporating sampling weights, strata, and primary sampling units [28], using the svydesign function from the nhanesR package in R software to create a survey design object. Descriptive statistics were reported as weighted proportions for categorical variables and weighted medians for continuous variables, stratified by pandemic period and glycemic status. Differences were assessed using Kruskal–Wallis tests for continuous variables and chi-square tests for categorical variables. Age-standardized prevalences of prediabetes and diabetes were estimated using direct standardization to the 2000 US Census population.

Multivariable logistic regression, stratified by sex and age, was performed to examine the associations between social, behavioral, and psychosocial determinants and glycemic outcomes. This analysis adjusted for potential confounders, including age, sex, and race/ethnicity. To evaluate whether associations varied across subgroups and shifted after the COVID-19 pandemic, we tested interaction terms between each determinant and the pandemic period (determinant × period). Variables were selected for interaction testing based on theoretical relevance and prior evidence of differential associations by sex, age, psychosocial burden, or behavioral risk. Interaction terms were included as cross-product variables in survey-weighted logistic regression models, with statistical significance assessed using Wald tests (two-sided α = 0.05). Effect modification was quantified using ratios of odds ratios (RORs) and 95% confidence intervals. Higher-order interactions were examined to assess complex heterogeneity. Three-way interactions (determinant × period × subgroup, such as sex or age group) were tested when the corresponding two-way interaction demonstrated suggestive evidence (p < 0.10) or strong theoretical justification. Additionally, four-way interactions (determinant × age group × sex × period) were evaluated to explore potential combined modification by demographic structure and pandemic context. To account for multiple comparisons arising from these analyzes, the Benjamini–Hochberg false discovery rate (FDR) correction was applied across all interaction tests, with FDR-adjusted q < 0.05 considered statistically significant.

The primary analyses were conducted using complete-case analysis (CCA). This approach was selected to maintain consistency of the analytic sample across multiple stratified and interaction models and to facilitate interpretability of higher-order interactions. Although missingness in NHANES is known to be socially patterned—particularly with respect to socioeconomic status, psychosocial factors, and health behaviors—CCA remains appropriate when accompanied by rigorous sensitivity analyses. Patterns of missingness were examined descriptively and were most consistent with a missing-at-random (MAR) mechanism, whereby missingness was associated with observed covariates but not directly with outcomes conditional on these variables. Missing-not-at-random (MNAR) mechanisms could not be formally excluded due to data limitations. To evaluate the potential impact of exclusion-related bias and to assess internal validity, sensitivity analyses were performed using multiple imputation by chained equations (MICE). Twenty imputed datasets were generated, incorporating all exposure variables, outcomes, covariates, and auxiliary variables used in the analytic models. Survey-weighted regression models were re-estimated within each imputed dataset, and results were pooled according to Rubin’s rules. Consistency in the direction, magnitude, and statistical significance of estimates between CCA and imputed analyses was interpreted as evidence of robustness.

All analyzes were performed using R software (version 4.5.1) with the ‘survey’ and ‘nhanesR’ packages. A two-sided P-value < 0.05 was considered statistically significant.

## Results

### Characteristics of the study population

This analysis included 7,766 US adults from the NHANES 2017–2020 (pre-pandemic) and 2021–2023 (post-pandemic) cycles, stratified by glycemic status. Age showed a significant increase only in the prediabetes group, rising from 54.0 to 58.0 years post-pandemic (Table 1). Notable socioeconomic shifts were observed: college graduation rates increased in the normoglycemia group from 37.9% to 48.6%, and health insurance coverage improved in both the normoglycemia (from 87.1% to 92.8%) and prediabetes groups (from 90.2% to 94.5%). Employment declined in the prediabetes group, with the percentage of non-employed individuals rising from 35.8% to 43.5%.

**Fig. 1:**
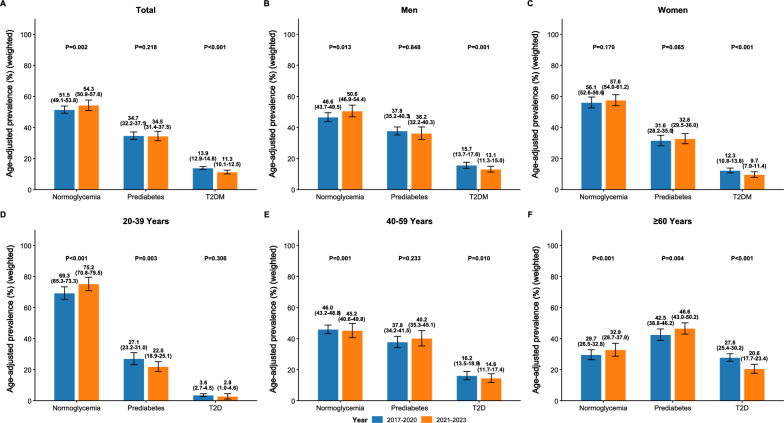
Age-adjusted prevalence of normoglycemia, prediabetes, and T2D pre- (2017–2020) and post- (2021–2023) COVID-19 pandemic. (**A**) Total population. (**B**) Men. (**C**) Women. (**D**) Ages 20–39 years. (**E**) Ages 40–59 years. (F) Ages ≥ 60 years. All prevalence estimates are age-standardized to the 2000 US Census population and incorporate NHANES survey weights. The y‑axis shows weighted, age‑adjusted prevalence (%) with 95% confidence intervals represented by error bars. Unweighted sample sizes (n) for each prevalence estimate are provided in Supplementary Table S1. P-values were derived from chi-square tests comparing the prevalence of each glycemic category between pre- and post-pandemic periods. Given multiple comparisons across three glycemic categories, p-values were adjusted using the Benjamini–Hochberg false discovery rate (FDR) correction, with FDR-adjusted q < 0.05 considered statistically significant

A comprehensive analysis of the age-adjusted prevalence revealed significant shifts in the glycemic landscape of US adults following the COVID-19 pandemic. In the overall population, a statistically significant increase was observed in the prevalence of normoglycemia, rising from 51.5% in the pre-pandemic period (2017–2020) to 54.3% in the post-pandemic period (2021–2023) (Fig. 1A and Table S1). Concurrently, the prevalence of type 2 diabetes demonstrated a significant decline from 13.9% to 11.3%. Prediabetes remained largely unchanged, from 34.7% to 34.5%, with no statistically significant difference (Table 1).

**Table 1:** Sociodemographic and clinical characteristics of the study population across glycemic status

Variables	Total (n = 7,766)	Normoglycemia (n = 3,364)			Prediabetes (n = 2,981)			T2D (n = 1,421)		
		2017–2020 (n = 2,000)	2021–2023 (n = 1,364)	P-value	2017–2020 (n = 1,804)	2021–2023 (n = 1,177)	P-value	2017–2020 (n = 964)	2021–2023 (n = 457)	P-value
Age years	50.0(34.0,63.0)	40.0(29.0,55.0)	41.0(30.0,58.0)	0.100	54.0(39.0,65.0)	58.0(42.0,67.0)	0.010	61.0(51.0,70.0)	61.0(51.0,68.0)	0.900
Sex				0.700			0.200			0.800
Men	51.5(47.2,55.9)	55.5(52.2,58.8)	54.7(51.8,57.7)		47.4(44.1,50.7)	50.6(47.1,54.2)		46.0(41.2,50.8)	45.2(39.4,51.0)	
Women	48.5(44.5,52.4)	44.5(41.2,47.8)	45.3(42.3,48.2)		52.6(49.3,55.9)	49.4(45.8,52.9)		54.0(49.2,58.8)	54.8(49.0,60.6)	
Race				0.400			0.900			0.600
Non-hispanic white	71.1(64.1,78.2)	73.3(68.4,78.2)	73.7(70.9,76.5)		69.6(65.7,73.5)	69.3(64.2,74.4)		68.1(61.4,74.9)	66.0(59.7,72.3)	
Non-hispanic black	9.7(7.8,11.7)	8.4(6.1,10.8)	7.4(5.5, 9.3)		11.0(8.3,13.7)	11.0(7.3,14.7)		12.2(8.5,15.9)	13.8(9.4,18.3)	
Mexican American	6.4(4.5, 8.3)	6.8(4.7, 9.0)	4.5(1.5, 7.5)		7.1(5.2, 9.1)	6.1(1.3,11.0)		7.9(5.6,10.3)	6.8(−0.5,14.1)	
Non-hispanic Asian	5.3(3.7, 6.8)	4.6(3.0,6.2)	5.8(3.1,8.4)		5.2(3.5,6.9)	6.2(3.4,8.9)		5.6(3.3,7.9)	3.8(0.5,7.1)	
Married status				0.500			1.000			0.400
Never married	17.9(15.7,20.2)	25.2(21.3,29.1)	22.5(19.4,25.6)		13.0(10.4,15.7)	13.4(11.3,15.4)		7.8(5.6,10.0)	10.1(7.5,12.7)	
Divorced, separated or widowed	18.1(16.3,19.8)	12.8(11.0,14.5)	13.4(11.0,15.8)		22.5(18.7,26.3)	22.5(19.9,25.1)		23.6(18.8,28.4)	25.4(19.5,31.4)	
Married or living with partner	64.0(58.7,69.3)	62.1(58.4,65.7)	64.1(61.3,66.9)		64.4(59.6,69.3)	64.2(60.8,67.6)		68.7(63.6,73.7)	64.5(57.8,71.1)	
Place of birth				0.600			0.900			0.500
US-born	84.0(77.1,91.0)	86.1(83.9,88.3)	85.1(82.6,87.7)		82.0(78.9,85.0)	82.1(79.6,84.6)		82.4(77.8,87.1)	85.1(79.8,90.3)	
Born outside the US	16.0(14.0,17.9)	13.9(11.7,16.1)	14.9(12.3,17.4)		18.0(15.0,21.1)	17.9(15.4,20.4)		17.6(12.9,22.2)	14.9(9.7,20.2)	
Educational levels				0.040			0.500			0.300
Less than college	28.9(26.4,31.4)	28.1(23.9,32.3)	21.3(16.4,26.1)		31.6(27.2,36.0)	27.8(22.2,33.5)		41.9(37.6,46.2)	35.8(27.9,43.7)	
Some college	32.7(30.0,35.5)	34.0(31.1,37.0)	30.1(24.4,35.8)		32.0(28.9,35.0)	32.2(27.9,36.5)		33.8(29.1,38.5)	39.4(35.8,43.0)	
College graduate or above	38.4(32.2,44.6)	37.9(32.6,43.2)	48.6(40.4,56.8)		36.4(30.6,42.2)	40.0(33.0,46.9)		24.3(19.2,29.4)	24.8(17.9,31.8)	
PIR levels				0.400			0.600			0.100
Low income	14.7(13.3,16.0)	16.2(14.3,18.1)	14.3(11.2,17.3)		14.0(11.5,16.4)	12.4(9.3,15.4)		15.9(13.5,18.2)	15.6(11.5,19.7)	
Middle income	34.5(31.4,37.7)	30.8(27.3,34.2)	34.0(29.4,38.6)		35.6(31.6,39.6)	34.3(29.4,39.2)		37.3(30.8,43.9)	48.0(39.7,56.3)	
High income	50.8(44.6,57.0)	53.0(48.7,57.3)	51.7(45.5,57.9)		50.4(46.2,54.7)	53.3(46.8,59.9)		46.8(40.4,53.3)	36.4(26.4,46.4)	
Health insurance	91.0(83.1,98.9)	87.1(84.1,90.0)	92.8(90.0,95.5)	0.010	90.2(87.9,92.4)	94.5(92.9,96.1)	0.003	92.4(89.8,94.9)	95.2(92.5,98.0)	0.200
Private insurance	65.0(58.3,71.7)	65.6(61.7,69.5)	68.1(63.6,72.6)	0.400	65.9(61.8,70.0)	65.5(61.9,69.2)	0.900	59.2(54.7,63.6)	54.2(46.8,61.5)	0.300
Smoking status				0.004			0.600			0.500
Never	61.2(55.1,67.2)	62.8(58.9,66.8)	68.8(65.4,72.3)		56.7(54.1,59.2)	59.6(53.7,65.5)		54.2(47.0,61.4)	54.5(48.5,60.4)	
Ex-smoker	25.2(22.9,27.4)	21.4(18.6,24.2)	21.8(19.0,24.5)		27.8(25.1,30.4)	25.6(22.2,28.9)		34.9(28.0,41.7)	31.7(24.9,38.5)	
Current smoker	13.7(12.2,15.1)	15.8(12.7,18.8)	9.4(7.0,11.8)		15.6(13.6,17.6)	14.8(11.0,18.7)		10.9(7.4,14.4)	13.8(10.4,17.2)	
Work				0.040			0.030			0.300
Not employed	36.7(33.6,39.9)	28.1(24.6,31.5)	32.9(29.9,35.9)		35.8(32.3,39.3)	43.5(40.1,46.8)		51.1(46.0,56.2)	56.5(52.9,60.2)	
Part-time employee	13.8(12.2,15.4)	17.4(14.7,20.1)	13.8(11.5,16.2)		13.2(11.0,15.4)	12.0(9.1,14.9)		9.9(6.9,12.9)	9.3(6.7,11.9)	
Full-time employee	49.5(45.0,53.9)	54.6(51.8,57.4)	53.2(50.9,55.6)		51.0(46.4,55.6)	44.5(40.1,48.9)		39.0(32.6,45.5)	34.1(31.0,37.3)	
Obesity	41.4(38.2,44.6)	31.9(28.3,35.4)	30.4(26.5,34.4)	0.600	46.6(41.7,51.6)	44.3(40.6,48.0)	0.500	67.1(62.5,71.6)	65.4(60.8,70.1)	0.600
Hypertension	38.4(35.5,41.3)	24.7(21.2,28.2)	24.9(21.7,28.2)	0.900	44.9(40.8,49.0)	44.0(39.8,48.2)	0.800	72.2(68.5,76.0)	67.6(63.1,72.1)	0.100
CVD	8.8(7.6,10.0)	4.0(2.8, 5.3)	4.2(2.8, 5.6)	0.800	9.2(7.1,11.3)	9.9(8.3,11.5)	0.600	24.4(18.8,30.1)	22.6(17.9,27.4)	0.600
Multimorbidity	36.9(33.9,39.8)	24.4(20.6,28.2)	25.6(23.1,28.0)	0.600	43.4(39.2,47.5)	40.3(37.0,43.5)	0.300	70.8(66.8,74.9)	57.4(53.0,61.8)	< 0.001
Depression levels				< 0.001			0.300			0.200
No/minimal depression	74.0(67.8,80.2)	77.9(75.7,80.1)	70.7(67.4,74.1)		75.8(71.7,80.0)	73.0(69.4,76.6)		71.6(67.2,76.1)	66.6(61.4,71.9)	
Depression symptoms	26.0(23.7,28.3)	22.1(19.9,24.3)	29.3(25.9,32.6)		24.2(20.0,28.3)	27.0(23.4,30.6)		28.4(23.9,32.8)	33.4(28.1,38.6)	

Data are presented as weighted medians (interquartile ranges) for continuous variables and percentages (95% confidence intervals) for categorical variables. All estimates are survey-weighted. PIR: poverty income ratio; T2D, type 2 diabetes; CVD: cardiovascular disease

Lifestyle changes were pronounced. Smoking decreased in the normoglycemia group, with current smokers dropping from 15.8% to 9.4%, while physical activity levels improved across all groups, particularly in type 2 diabetes, where high activity levels increased from 15.2% to 26.2% (Table 2). Dietary intake also declined significantly in the prediabetes group, including reductions in energy intake (from 2073 to 1924 kcal), carbohydrates, and sodium. Additionally, symptoms of depression rose in the normoglycemia group, increasing from 22.1% to 29.3%. Clinical profiles remained largely stable, although multimorbidity decreased significantly in the type 2 diabetes group, falling from 70.8% to 57.4%.

Multivariable logistic regression and interaction analyzes revealed that the associations between sociodemographic, psychosocial, and lifestyle factors and prediabetes and type 2 diabetes were significantly modified by gender and the COVID-19 pandemic period (Table 2).

**Table 2:** Biochemical parameters and lifestyle behaviors of the study population across glycemic status

Variables	Total (n = 7,766)	Normoglycemia (n = 3,364)			Prediabetes (n = 2,981)			T2D (n = 1,421)		
		2017–2020 (n = 2,000)	2021–2023 (n = 1,364)	P-value	2017–2020 (n = 1,804)	2021–2023 (n = 1,177)	P-value	2017–2020 (n = 964)	2021–2023 (n = 457)	P-value
BMI, kg/m2	28.5(24.7,33.4)	26.9(23.4,31.3)	26.7(23.6,31.2)	0.800	29.2(25.9,34.4)	29.1(25.6,33.7)	0.300	32.5(28.7,36.9)	33.3(28.1,37.2)	1.000
WC, cm	99.3(88.8,111.1)	93.0(84.0,104.8)	94.1(84.0,105.4)	0.700	102.3(93.4,113.0)	101.4(92.5,112.0)	0.700	111.8(101.8,121.9)	111.4(100.5,121.8)	0.800
WHR	0.9(0.9,1.0)	0.9(0.8,1.0)	0.9(0.8,1.0)	1.000	1.0(0.9,1.0)	0.9(0.9,1.0)	0.800	1.0(0.9,1.0)	1.0(0.9,1.0)	0.400
WHtR	0.6(0.5,0.7)	0.6(0.5,0.6)	0.6(0.5,0.6)	0.900	0.6(0.6,0.7)	0.6(0.5,0.7)	0.800	0.7(0.6,0.7)	0.7(0.6,0.7)	1.000
FPG, mmol/L	5.6(5.3,6.1)	5.3(5.0,5.4)	5.2(5.0,5.4)	0.200	5.9(5.7,6.2)	5.8(5.6,6.1)	0.020	7.5(6.9,9.4)	7.4(6.7,9.6)	0.900
HbA1c, %	5.5(5.2,5.8)	5.3(5.1,5.4)	5.3(5.0,5.4)	1.000	5.7(5.4,5.8)	5.7(5.4,5.9)	0.800	6.7(6.1,7.6)	6.8(6.3,7.7)	0.200
SBP	119.3(109.7,130.0)	115.7(107.3,126.0)	116.0(108.0,126.7)	0.500	122.0(113.3,132.7)	121.5(111.0,132.3)	0.200	124.7(113.3,137.3)	124.3(115.0,133.3)	0.600
DBP	73.7(67.0,81.0)	71.7(65.3,79.0)	73.7(67.3,80.0)	0.003	74.7(68.3,82.0)	75.3(68.7,82.7)	0.800	74.0(67.7,82.5)	75.3(68.0,83.0)	0.300
TC, mmol/L	4.8(4.2,5.5)	4.8(4.2,5.4)	4.9(4.3,5.5)	0.020	4.9(4.3,5.7)	5.0(4.3,5.7)	0.600	4.4(3.8,5.3)	4.3(3.6,5.1)	0.400
HDL-C, mmol/L	1.3(1.1,1.6)	1.4(1.2,1.7)	1.4(1.2,1.7)	0.900	1.3(1.1,1.6)	1.3(1.1,1.6)	0.100	1.1(1.0,1.4)	1.2(1.0,1.4)	0.010
Dietary composition										
Energy intake, kcal	1964.0(1450.0,2594.0)	2010.0(1496.0,2652.0)	1869.0(1408.0,2472.0)	0.004	2073.0(1584.0,2749.0)	1924.0(1410.0,2480.0)	< 0.001	1934.0(1374.0,2580.0)	1909.0(1384.0,2555.0)	0.700
Carbohydrates, g	218.4(155.8,298.0)	225.1(158.4,304.2)	206.2(150.9,288.0)	0.020	233.2(165.2,317.0)	212.4(149.3,281.4)	< 0.001	210.6(156.8,284.9)	208.4(148.0,278.1)	0.500
Sugars, g	85.2(51.9,129.6)	88.7(51.8,133.7)	80.0(48.2,121.5)	0.003	95.0(57.0,137.5)	83.2(50.8,123.6)	< 0.001	83.2(51.4,128.1)	80.4(53.0,118.4)	0.500
Dietary, fiber, g	14.6(9.5,21.2)	14.5(9.4,21.0)	14.8(10.0,21.4)	0.400	15.0(9.6,22.1)	14.3(9.2,21.9)	0.200	14.4(9.3,20.0)	13.7(9.4,19.8)	0.900
Proteins, g	72.3(52.1,99.2)	73.2(53.6,101.3)	68.9(49.6, 95.9)	0.100	77.4(54.9,104.0)	68.5(51.6, 91.8)	0.001	73.0(50.7, 99.6)	70.3(50.1, 96.6)	0.500
Fats, g	79.9(56.0,111.8)	80.5(55.7,113.8)	76.6(54.0,106.6)	0.100	83.5(58.8,116.8)	78.9(54.6,108.3)	0.100	79.6(57.4,111.4)	79.4(53.6,114.3)	0.900
Saturated fats, g	25.4(16.6,37.0)	26.2(16.8,38.6)	23.3(15.6,34.5)	0.001	27.0(17.7,37.7)	24.8(16.3,35.1)	0.040	25.9(16.8,36.9)	25.2(17.1,36.8)	0.700
Polyunsaturated fats, g	17.7(11.6,26.3)	17.7(11.7,25.9)	16.9(11.3,25.4)	0.400	18.3(12.3,28.7)	17.7(11.7,25.1)	0.040	18.0(11.4,26.1)	17.7(11.0,27.5)	0.700
Cholesterol, mg	245.0(134.0,428.0)	255.0(138.0,408.0)	216.0(123.0,404.0)	0.040	249.0(141.0,440.0)	243.0(138.0,439.0)	0.600	264.0(143.0,454.0)	244.0(138.0,465.0)	0.600
Linoleic acid, g	15.7(10.1,23.2)	15.6(10.3,22.8)	15.0(9.7,22.6)	0.300	16.2(10.7,25.4)	15.7(10.1,22.1)	0.030	16.1(10.1,22.9)	15.8(9.7,24.7)	0.600
Vitamin D, µg	3.0(1.2,5.7)	2.9(1.1,5.4)	2.9(1.2,5.8)	0.500	3.1(1.2,5.7)	3.2(1.3,5.9)	0.700	3.3(1.4,5.4)	3.4(1.2,6.1)	0.600
Vitamin E, mg	8.0(5.2,11.9)	7.7(5.1,11.7)	8.2(5.3,12.1)	0.500	8.3(5.4,12.0)	8.1(5.4,12.1)	0.400	7.8(4.6,11.7)	7.8(4.9,12.3)	0.600
Vitamin K, µg	77.5(43.4,142.9)	73.7(41.9,138.0)	82.0(44.9,150.0)	0.300	83.9(46.1,151.3)	78.7(45.3,138.4)	0.400	72.2(41.6,132.3)	69.3(40.7,130.8)	0.700
Sodium, mg	3049.0(2188.0,4200.0)	3197.0(2263.0,4294.0)	2874.0(2077.0,3956.0)	0.010	3302.0(2435.0,4442.0)	2768.0(2071.0,3873.0)	< 0.001	3154.0(2118.0,4209.0)	2990.0(2081.0,4239.0)	0.500
Magnesium, mg	274.0(200.0,372.0)	275.0(203.0,378.0)	277.0(199.0,369.0)	0.700	287.0(212.0,381.0)	263.0(195.0,371.0)	0.010	264.0(188.0,359.0)	264.0(188.0,352.0)	0.900
Selenium, µg	100.2(69.7,138.7)	101.8(71.1,140.1)	97.0(65.5,130.0)	0.040	107.5(75.3,143.3)	94.7(67.9,132.4)	0.002	99.8(70.9,141.5)	98.4(64.9,133.1)	0.400
Caffeine, mg	128.0(36.0,240.0)	129.0(36.0,238.0)	120.0(27.0,205.0)	0.100	144.0(44.0,260.0)	118.0(37.0,218.0)	0.010	134.0(43.0,257.0)	121.0(42.0,229.0)	0.900
CDAI	−0.1(−2.0, 2.3)	0.2(−2.1,2.4)	−0.1(−2.2,2.1)	0.500	0.1(−1.8,2.6)	−0.3(−2.2,2.1)	0.020	−0.3(−1.9,2.2)	−0.6(−2.2,1.6)	0.300
DII	1.8(0.2,3.0)	1.8(0.2,3.1)	1.7(0.2,3.0)	0.800	1.6(0.1,2.9)	1.9(0.2,3.0)	0.100	2.0(0.3,3.2)	1.9(0.6,3.2)	0.500
Alcohol consumption, g/week	16.1(3.2,64.4)	24.2(4.8,80.5)	24.2(4.8,80.5)	0.600	14.0(3.2,55.8)	24.2(3.2,83.8)	0.100	7.2(1.2,27.9)	8.1(2.4,32.2)	0.300
Sedentary time, min/day	360.0(240.0,480.0)	300.0(180.0,480.0)	360.0(240.0,540.0)	0.030	300.0(180.0,480.0)	300.0(180.0,480.0)	0.700	360.0(240.0,480.0)	360.0(240.0,480.0)	0.700
PA levels				< 0.001			< 0.001			0.010
Low active	51.7(48.0,55.5)	50.8(46.4,55.2)	37.2(33.6,40.9)		62.9(58.2,67.6)	47.1(42.3,52.0)		70.7(65.0,76.3)	57.4(51.1,63.7)	
Moderate active	15.9(13.8,17.9)	13.3(11.4,15.2)	20.3(18.0,22.7)		13.0(9.8,16.1)	18.8(16.3,21.3)		14.1(9.5,18.7)	16.4(13.1,19.6)	
High active	32.4(28.7,36.1)	36.0(31.5,40.4)	42.5(38.4,46.5)		24.1(21.3,26.9)	34.1(29.7,38.5)		15.2(10.8,19.6)	26.2(19.5,32.9)	
Sleep hours (weekdays)				0.400			0.005			0.010
< 6 h	7.8(6.8, 8.8)	6.4(5.0, 7.8)	5.6(4.6, 6.6)		10.7(8.2,13.1)	7.9(5.5,10.2)		8.8(6.3,11.3)	10.8(6.8,14.8)	
6–8 h	44.2(40.4,48.0)	45.0(42.2,47.7)	43.3(39.9,46.7)		47.7(44.4,50.9)	40.9(38.0,43.8)		47.8(43.7,52.0)	35.4(28.7,42.0)	
≥ 8 h	48.0(43.9,52.2)	48.7(45.6,51.7)	51.1(47.3,54.8)		41.7(37.0,46.4)	51.2(48.2,54.3)		43.4(38.8,47.9)	53.9(47.1,60.7)	
Sleep hours (weekends)				0.500			0.040			1.000
< 6 h	19.2(17.7,20.6)	19.2(16.7,21.6)	17.1(14.0,20.3)		20.2(17.8,22.6)	18.6(15.3,21.8)		21.6(18.2,25.1)	21.4(17.8,25.0)	
6–8 h	26.6(23.9,29.3)	23.9(21.4,26.4)	23.3(19.5,27.2)		31.3(28.1,34.6)	26.3(23.3,29.4)		30.5(25.5,35.6)	30.0(24.8,35.3)	
≥ 8 h	54.2(49.2,59.3)	56.9(53.6,60.2)	59.5(55.7,63.3)		48.5(44.6,52.4)	55.1(52.3,57.9)		47.8(43.2,52.5)	48.5(42.9,54.1)	

Data are presented as weighted medians (interquartile ranges) for continuous variables and percentages (95% confidence intervals) for categorical variables. All estimates are survey-weighted. T2D, type 2 diabetes; BMI: body mass index; WC: waist circumference; WHR: waist-to-hip ratio; WHtR: waist-to-height ratio; FPG: fasting plasma glucose; SBP: systolic blood pressure; DBP: diastolic blood pressure; TC: total cholesterol; HDL-C: high-density lipoprotein cholesterol; CDAI: composite dietary antioxidant index; DII: dietary inflammatory index; PA, physical activity

### Prevalence of prediabetes and type 2 diabetes pre- and post-COVID-19 pandemic

Sex-stratified analyzes revealed notable differential trends between men and women. Among men, the pattern mirrored the overall population, with a significant increase in normoglycemia prevalence from 46.6% to 50.6% (Fig. 1B), accompanied by a significant decrease in type 2 diabetes prevalence from 15.7% to 13.1%. The prevalence of prediabetes among men remained stable (37.8% to 36.2%, p = 0.848). While a non-significant increase in normoglycemia was observed in women (from 56.1% to 57.6%) (Fig. 1C), women experienced a notable and significant reduction in type 2 diabetes prevalence, which declined from 12.3% to 9.7%. Furthermore, a borderline non-significant increase was detected in the prevalence of prediabetes among women, rising from 31.6% to 32.8%, a trend not observed in men.

Age-stratified analyzes revealed distinct temporal patterns across different life stages. Among adults aged 20–39 years, the glycemic profile improved substantially, marked by a significant increase in normoglycemia (from 69.3% to 75.2%, p < 0.001) and notable reductions in both prediabetes (from 27.1% to 22.0%, p = 0.003) and type 2 diabetes (from 3.6% to 2.8%, p = 0.306), though the latter did not reach statistical significance (Fig. 1D). In contrast, middle-aged adults (40–59 years) exhibited a more complex pattern, with a modest increase in normoglycemia (from 46.0% to 45.2%, p = 0.001) and a non-significant rise in prediabetes (from 37.8% to 40.2%, p = 0.233), alongside a significant decrease in type 2 diabetes (from 16.2% to 14.6%, p = 0.010) (Fig. 1E). Most strikingly, adults aged 60 years and older demonstrated the most pronounced changes, with significant improvements in both normoglycemia (from 29.7% to 32.9%, p < 0.001) and type 2 diabetes (from 27.8% to 20.6%, p < 0.001), albeit at the cost of a significant increase in prediabetes prevalence (from 42.5% to 46.6%, p = 0.004) (Fig. 1F).

### Associations and interactions of social, psychosocial, and lifestyle determinants with prediabetes and type 2 diabetes pre- and post-COVID-19 pandemic

Sociodemographic factors showed dynamic associations with diabetes risk. Higher education consistently protected against type 2 diabetes, with college graduates exhibiting lower risk in both pre-pandemic (OR 0.49, 95% CI: 0.33–0.73) and pandemic periods (OR 0.44, 95% CI: 0.29–0.65) (Table 3). Age-stratified analysis refined this finding, revealing that among adults aged 40–59, this protective effect was particularly consistent both before (OR 0.42, 95% CI: 0.0.25–0.71) and during the pandemic (OR 0.40, 95% CI: 0.21–0.75) (Table S2).

**Table 3:** Associations of social, psychosocial, and lifestyle determinants with prediabetes and type 2 diabetes pre- and post-COVID-19 pandemic

Variables		Prediabetes (n = 2,981)				T2D (n = 1,421)			
		2017–2020 (n = 1,804)		2017–2020 (n = 964)		2017–2020 (n = 964)		2021–2023 (n = 457)	
		OR (95%CI)	P-value	OR (95%CI)	P-value	OR (95%CI)	P-value	OR (95%CI)	P-value
Educational levels	Less than college	Reference		Reference		Reference		Reference	
	Some college	0.98 (0.79–1.22)	0.877	0.97 (0.77–1.22)	0.808	0.81 (0.61–1.07)	0.154	1.01 (0.80–1.26)	0.957
	College graduate or above	1.11 (0.87–1.42)	0.422	0.88 (0.68–1.14)	0.362	0.49 (0.33–0.73)	0.002	0.44 (0.29–0.65)	0.004
Married status	Never married	Reference		Reference		Reference		Reference	
	Divorced, separated or widowed	1.59 (1.14–2.23)	0.014	1.15 (0.86–1.54)	0.367	1.03 (0.69–1.55)	0.881	1.10 (0.69–1.75)	0.698
	Married or living with partner	1.28 (0.96–1.71)	0.106	1.05 (0.81–1.36)	0.728	1.27 (0.89–1.81)	0.202	1.13 (0.77–1.67)	0.553
Place of birth	US-born	Reference		Reference		Reference		Reference	
	Born outside the US	1.29 (1.04–1.59)	0.033	1.20 (0.94–1.54)	0.167	0.97 (0.60–1.58)	0.917	0.71 (0.41–1.21)	0.238
PIR levels	Low income	Reference		Reference		Reference		Reference	
	Middle income	1.19 (0.96–1.47)	0.132	1.02 (0.82–1.26)	0.872	0.86 (0.72–1.02)	0.105	1.08 (0.82–1.43)	0.600
	High income	1.08 (0.88–1.33)	0.472	1.24 (1.00–1.54)	0.084	0.74 (0.58–0.94)	0.025	0.56 (0.39–0.81)	0.014
Work	Not-employed	Reference		Reference		Reference		Reference	
	Part-time employee	1.08 (0.80–1.47)	0.612	1.11 (0.82–1.51)	0.509	0.71 (0.48–1.05)	0.101	0.69 (0.48–0.98)	0.071
	Full-time employee	1.32 (1.04–1.67)	0.034	1.20 (0.96–1.49)	0.143	0.96 (0.73–1.26)	0.781	0.81 (0.67–0.97)	0.055
Health insurance	No	Reference		Reference		Reference		Reference	
	Yes	1.05 (0.80–1.37)	0.723	1.12 (0.80–1.57)	0.517	1.02 (0.76–1.37)	0.898	1.24 (0.66–2.35)	0.524
Depression levels	No/minimal depression	Reference		Reference		Reference		Reference	
	Depression-symptoms	1.06 (0.84–1.33)	0.645	1.01 (0.81–1.26)	0.929	1.44 (1.21–1.72)	< 0.001	1.61 (1.21–2.12)	0.009
Smoking status	Never	Reference		Reference		Reference		Reference	
	Ex-smoker	1.11 (0.90–1.36)	0.345	1.01 (0.80–1.27)	0.962	1.31 (0.88–1.95)	0.201	1.33 (0.99–1.77)	0.092
	Current smoker	1.24 (1.05–1.47)	0.024	1.65 (1.21–2.24)	0.014	0.95 (0.62–1.45)	0.806	1.48 (1.04–2.10)	0.060
PA_levels	Low active	Reference		Reference		Reference		Reference	
	Moderate active	0.86 (0.62–1.19)	0.378	0.89 (0.72–1.10)	0.310	0.89 (0.57–1.39)	0.618	0.63 (0.50–0.79)	0.004
	High active	0.78 (0.60–1.02)	0.090	0.87 (0.68–1.10)	0.273	0.53 (0.37–0.74)	0.002	0.57 (0.38–0.85)	0.026
Sleep hours weekdays	< 6 h	Reference		Reference		Reference		Reference	
	6–8 h	0.70 (0.54–0.91)	0.018	0.93 (0.67–1.29)	0.683	1.05 (0.74–1.50)	0.795	0.59 (0.35–1.02)	0.094
	≥ 8 h	0.57 (0.39–0.83)	0.009	0.91 (0.60–1.40)	0.691	0.83 (0.53–1.28)	0.404	0.70 (0.39–1.26)	0.269
Sleep hours weekends	< 6 h	Reference		Reference		Reference		Reference	
	6–8 h	1.12 (0.87–1.45)	0.387	0.90 (0.68–1.21)	0.518	0.85 (0.59–1.21)	0.372	0.90 (0.61–1.31)	0.591
	≥ 8 h	0.85 (0.66–1.09)	0.214	0.91 (0.72–1.16)	0.479	0.74 (0.56–0.97)	0.042	0.69 (0.53–0.90)	0.026
Energy intake, kcal		1.13 (1.04–1.22)	0.009	1.07 (0.96–1.20)	0.264	0.93 (0.82–1.06)	0.310	1.10 (0.93–1.30)	0.312
Alcohol_consumption_g_week		0.99 (0.90–1.08)	0.797	1.11 (1.01–1.21)	0.058	0.76 (0.52–1.11)	0.176	0.82 (0.57–1.18)	0.315
CDAI		1.05 (0.94–1.17)	0.397	1.03 (0.93–1.12)	0.614	0.89 (0.78–1.00)	0.067	0.93 (0.80–1.07)	0.330
DII		0.93 (0.84–1.04)	0.209	0.99 (0.89–1.09)	0.803	1.18 (1.06–1.31)	0.006	1.12 (1.00–1.26)	0.077
Hypertension	No	Reference		Reference		Reference		Reference	
	Yes	1.11 (0.91–1.35)	0.310	1.05 (0.78–1.41)	0.749	3.30 (2.57–4.24)	< 0.001	2.81 (2.19–3.61)	< 0.001
Obesity	No	Reference		Reference		Reference		Reference	
	Yes	1.29 (1.06–1.58)	0.021	1.33 (1.06–1.66)	0.033	3.85 (3.03–4.90)	< 0.001	3.65 (3.14–4.25)	< 0.001
Multimorbidity	No	Reference		Reference		Reference		Reference	
	Yes	1.07 (0.86–1.34)	0.541	1.07 (0.88–1.31)	0.495	3.27 (2.46–4.34)	< 0.001	2.03 (1.65–2.50)	< 0.001

Models were adjusted for age, sex, and race. All models were estimated using survey-weighted logistic regression. Sample sizes for each model and subgroup are reported. Variables included in the models were prespecified a priori based on prior literature and theoretical relevance to glycemic regulation. T2D, type 2 diabetes; PIR: poverty income ratio; CDAI: composite dietary antioxidant index; DII: dietary inflammatory index; PA, physical activity

Income effects varied by sex and period. During the pandemic, high income was protective against type 2 diabetes overall (OR 0.56, 95% CI: 0.39–0.81), driven mainly by women (OR 0.43, 95% CI: 0.27–0.71) (Table S3). Conversely, among men, high income emerged as a risk factor for prediabetes (OR 1.54, 95% CI: 1.13–2.08). Age-stratified analysis further showed this protection was significant in the 40–59 age group during the pandemic (OR 0.49, 95% CI: 0.31–0.78). Notably, the impact of high income was further influenced by multimorbidity status. Interaction analysis revealed that during the pandemic, the presence of multimorbidity significantly strengthened the positive association between high income and prediabetes (ROR = 2.59, 95% CI: 1.17–5.71, P = 0.028). In stark contrast, for type 2 diabetes, multimorbidity substantially weakened the protective association of high income (ROR = 0.32, 95% CI: 0.11–0.91, P = 0.044).

In older adults (≥ 60 years), being married or divorced was associated with a protective effect against prediabetes before the pandemic; however, this protective effect was no longer observed during the pandemic. Similarly, nativity effects varied, with foreign birth associated with increased prediabetes risk pre-pandemic (OR 1.29, 95% CI: 1.04–1.59), particularly among women (OR 1.54, 95% CI: 1.09–2.18).

Behavioral and psychosocial interactions revealed a striking pattern of effect modification by depressive symptoms, which differed significantly between prediabetes and type 2 diabetes. For prediabetes, depressive symptoms consistently mitigated the risk-enhancing effects of behavioral factors during the pandemic. This protective moderation is evidenced by significant three-way interactions for both current smoking (ROR 0.36, 95% CI: 0.16–0.83) and alcohol consumption (ROR 0.75, 95% CI: 0.57–0.99) (Table 4 and Table S4). In contrast, for type 2 diabetes, depressive symptoms amplified the risk effects of these same behaviors, demonstrating a strong synergistic effect. This is shown by significant three-way interactions for current smoking (ROR 3.17, 95% CI: 1.32–7.61) and alcohol consumption (ROR 4.79, 95% CI: 1.96–11.71).

**Table 4:** Significant interaction effects of social, psychosocial, and lifestyle determinants with sex, age, and pandemic period on prediabetes and type 2 diabetes

Disease	Interaction Term	ROR (95%CI)	β (SE)	P-interaction
Prediabetes	≥ 60 years × US-born × pandemic	1.59 (1.07–2.38)	0.47 (0.20)	0.032
	40–59 years × US-born × pandemic	1.41 (1.05–1.90)	0.34 (0.15)	0.033
	Alcohol consumption × depression symptoms × pandemic	0.75 (0.57–0.99)	−0.28 (0.14)	0.048
	Alcohol consumption × no/minimal depression × pandemic	1.25 (1.04–1.50)	0.22 (0.09)	0.025
	College graduate or above × men × pre-pandemic	1.56 (1.11–2.18)	0.44 (0.17)	0.017
	Current smoker × depression symptoms × pandemic	0.36 (0.16–0.83)	−1.01 (0.42)	0.025
	Current smoker × no/minimal depression × pandemic	2.02 (1.32–3.10)	0.71 (0.22)	0.004
	Cepression symptoms × ≥ 60 years × pre-pandemic	0.48 (0.27–0.86)	−0.74 (0.30)	0.022
	DII × ≥ 60 years × pre-pandemic	0.70 (0.55–0.90)	−0.35 (0.12)	0.009
	DII × 40–59 years × pre-pandemic	0.72 (0.57–0.89)	−0.33 (0.11)	0.007
	Divorced, separated or widowed × ≥ 60 years × pre-pandemic	0.20 (0.07–0.54)	−1.62 (0.51)	0.006
	Ex-smoker × ≥ 60 years × pre-pandemic	0.39 (0.20–0.75)	−0.94 (0.34)	0.012
	Health insurance × 40–59 years × pre-pandemic	0.54 (0.32–0.92)	−0.61 (0.27)	0.034
	High active × ≥ 60 years × pre-pandemic	1.93 (1.38–2.70)	0.66 (0.17)	0.001
	Low active × ≥ 60 years × pandemic	1.72 (1.09–2.70)	0.54 (0.23)	0.031
	Low active × 40–59 years × pandemic	1.89 (1.29–2.78)	0.64 (0.20)	0.004
	Married or living with partner × ≥ 60 years × pandemic	3.96 (1.54–10.17)	1.38 (0.48)	0.011
	Married or living with partner × ≥ 60 years × pre-pandemic	0.25 (0.12–0.52)	−1.40 (0.38)	0.002
	Moderate active × 40–59 years × pre-pandemic	2.56 (1.42–4.61)	0.94 (0.30)	0.006
	Sleep hours (weekdays): 6–8 h × men × pre-pandemic	0.50 (0.30–0.85)	−0.69 (0.27)	0.018
	Multimorbidity × men × pre-pandemic	0.72 (0.55–0.94)	−0.33 (0.14)	0.025
	Non-multimorbidity × alcohol consumption × pandemic	1.21 (1.05–1.41)	0.19 (0.07)	0.015
	Multimorbidity × high income × pandemic	2.59 (1.17–5.71)	0.95 (0.40)	0.028
	Non-multimorbidity × middle income × pandemic	0.63 (0.42–0.94)	−0.47 (0.21)	0.036
	Multimorbidity × current smoker × pandemic	0.31 (0.14–0.66)	−1.18 (0.39)	0.006
	Non-multimorbidity × current smoker × pandemic	2.31 (1.41–3.78)	0.84 (0.25)	0.003
T2D	Alcohol consumption × depression symptoms × pre-pandemic	0.37 (0.17–0.83)	−0.99 (0.41)	0.022
	Alcohol consumption × depression symptoms × pandemic	4.79 (1.96–11.71)	1.57 (0.46)	0.002
	Alcohol consumption × men × pre-pandemic	2.65 (1.20–5.84)	0.97 (0.40)	0.023
	Born outside the US × ≥ 60 years × pre-pandemic	0.36 (0.17–0.77)	−1.02 (0.38)	0.014
	Born outside the US × ≥ 60 years × pandemic	8.41 (1.41–50.31)	2.13 (0.91)	0.029
	Born outside the US × 40–59 years × pre-pandemic	0.27 (0.12–0.60)	−1.31 (0.40)	0.004
	Born outside the US × 40–59 years × pandemic	8.86 (1.47–53.43)	2.18 (0.92)	0.026
	Born outside the US × pandemic	0.11 (0.02–0.61)	−2.17 (0.85)	0.018
	CDAI × ≥ 60 years × pandemic	2.38 (1.42–3.97)	0.87 (0.26)	0.003
	CDAI × 40–59 years × pandemic	2.80 (1.58–4.94)	1.03 (0.29)	0.002
	CDAI × pandemic	0.46 (0.28–0.76)	−0.78 (0.25)	0.005
	Current smoker × depression symptoms × pandemic	3.17 (1.32–7.61)	1.15 (0.45)	0.017
	Depression symptoms × men × pre-pandemic	0.58 (0.35–0.97)	−0.55 (0.26)	0.046
	DII × ≥ 60 years × pandemic	0.38 (0.22–0.66)	−0.97 (0.28)	0.002
	DII × 40–59 years × pre-pandemic	1.61 (1.11–2.35)	0.48 (0.19)	0.02
	DII × pandemic	2.47 (1.47–4.16)	0.91 (0.27)	0.002
	Full-time employee × men × pre-pandemic	0.53 (0.35–0.83)	−0.63 (0.22)	0.01
	Health insurance × men × pre-pandemic	3.04 (1.54–5.97)	1.11 (0.35)	0.003
	Married or living with partner × pandemic	0.55 (0.35–0.87)	−0.59 (0.23)	0.017
	Obesity × men × pandemic	1.83 (1.04–3.24)	0.61 (0.29)	0.046
	Sleep hours (weekends) ≥ 8 h × ≥ 60 years × pandemic	4.46 (1.26–15.86)	1.50 (0.65)	0.034
	Sleep hours (weekends) ≥ 8 h × 40–59 years × pandemic	5.64 (1.25–25.51)	1.73 (0.77)	0.038
	Sleep hours (weekends) ≥ 8 h × pandemic	0.21 (0.06–0.70)	−1.58 (0.63)	0.022
	Sleep hours (weekdays): 6–8 h × pandemic	0.53 (0.31–0.92)	−0.63 (0.28)	0.03
	Multimorbidity × women × pandemic	0.51 (0.32–0.79)	−0.68 (0.23)	0.006
	Multimorbidity × pandemic	0.54 (0.40–0.74)	−0.61 (0.16)	0.001
	Multimorbidity × not-employed × pandemic	0.52 (0.34–0.81)	−0.65 (0.22)	0.008
	Multimorbidity × born outside the US × pandemic	2.03 (1.06–3.89)	0.71 (0.33)	0.041
	Non-multimorbidity × born outside the US × pandemic	0.51 (0.30–0.84)	−0.68 (0.26)	0.014
	Multimorbidity × CDAI × pre-pandemic	0.71 (0.59–0.87)	−0.34 (0.10)	0.002
	Multimorbidity × CDAI × pandemic	1.59 (1.16–2.17)	0.46 (0.16)	0.008
	Multimorbidity × no/minimal depression × pandemic	0.46 (0.31–0.68)	−0.77 (0.20)	0.001
	Multimorbidity × low active × pandemic	0.59 (0.39–0.87)	−0.53 (0.20)	0.015
	Multimorbidity × high income × pandemic	0.32 (0.11–0.91)	−1.14 (0.53)	0.044
	Non-multimorbidity × middle income × pandemic	2.10 (1.06–4.14)	0.74 (0.35)	0.044
	Multimorbidity × sleep hours (weekends) < 6 h × pandemic	0.43 (0.19–0.94)	−0.85 (0.40)	0.045
	Multimorbidity × never-smoker × pandemic	0.55 (0.36–0.85)	−0.59 (0.22)	0.012

Models were adjusted for age, sex, and race. Only interactions reaching statistical significance after false discovery rate (FDR) correction (q < 0.05) are shown. Full results, including non-significant interactions, are provided in Supplementary Table S4. Reference groups: pre-pandemic period, less than college, never married, men, never-smoker, US-born, low income, not employed, no/minimal depression, no health insurance, low active, 20–39 years old, non-obese, non-hypertension, and < 6 h sleep. CDAI: composite dietary antioxidant index; DII: dietary inflammatory index; ROR, ratio of odds ratios; T2D, type 2 diabetes

High physical activity was protective against prediabetes in young adults (ages 20–39) pre-pandemic (OR = 0.53, 95% CI: 0.37–0.74), but this effect diminished and became non-significant during the pandemic (OR = 0.86, 95% CI: 0.57–1.29). In contrast, among adults aged ≥ 60, high physical activity was not associated with prediabetes risk in either period (pre-pandemic OR = 1.03, 95% CI: 0.67–1.59; pandemic OR = 1.01, 95% CI: 0.76–1.33). Notably, for the more advanced endpoint of type 2 diabetes, high physical activity remained strongly and consistently protective in the ≥ 60 age group both before (OR = 0.49, 95% CI: 0.34–0.72) and during the pandemic (OR = 0.48, 95% CI: 0.31–0.72).

Sleep duration of 6–8 h on weekdays was protective against prediabetes pre-pandemic in the overall population (OR 0.70, 95% CI: 0.54–0.91). This association was driven entirely by men (OR 0.54, 95% CI: 0.37–0.79), with no effect observed in women (OR 0.98, 95% CI: 0.68–1.40). A significant interaction (sleep × men × pre-pandemic, ROR 0.50, 95% CI: 0.30–0.85) confirmed this sex-specific effect, which disappeared during the pandemic. Age-stratified analyzes revealed distinct protective sleep patterns during the pandemic: weekend sleep of ≥ 8 h was associated with a significantly lower risk of type 2 diabetes among adults aged 20–39 (OR 0.16, 95% CI: 0.06–0.41), while weekday sleep of ≥ 8 h provided protection for individuals in the 40–59 year age group (OR 0.48, 95% CI: 0.27–0.84).

Analysis of dietary indices revealed distinct patterns. Before the pandemic, higher DII was significantly associated with increased odds of type 2 diabetes in men (OR = 1.23, 95% CI: 1.06–1.42). Age-stratified results indicated that a higher DII was protectively associated with prediabetes in older adults pre-pandemic. Pre-pandemic, the risk for type 2 diabetes was concentrated in individuals aged 40–59 years (OR 1.32, 95% CI: 1.11–1.58), but during the pandemic, the risk transitioned to a significant factor among adults aged 20–39 years (OR 2.10, 95% CI: 1.36–3.26). Furthermore, analyses of the CDAI revealed a significant temporal and age-dependent shift in its association with type 2 diabetes. In the overall population, CDAI exhibited a borderline non-significant protective trend pre-pandemic (OR = 0.89, 95% CI: 0.78–1.00), which diminished during the pandemic. Crucially, interaction analyses indicated that the protective association of a higher CDAI was strongly influenced by age and time period. Specifically, during the pandemic, a higher CDAI was linked to a significantly stronger protective effect against type 2 diabetes among adults aged 40–59 years (ROR = 2.80, 95% CI: 1.58–4.94) and those aged 60 years and older (ROR = 2.38, 95% CI: 1.42–3.97).

Multimorbidity, hypertension, and obesity were strongly and consistently associated with type 2 diabetes across all subgroups. Hypertension (OR range: 2.09–8.84), obesity (OR range: 1.80–15.97), and multimorbidity (OR range: 1.81–4.28) demonstrated significant effects. For prediabetes, obesity was associated with higher odds in adults aged 20–39 during the pandemic (OR = 1.80, 95% CI: 1.20–2.71, P = 0.019) as well as in the overall population. Multimorbidity significantly interacted with multiple factors: individuals with multimorbidity who were born outside the US had a relative odds ratio (ROR) of 2.03 (95% CI: 1.06–3.89, P = 0.041) for type 2 diabetes compared to their counterparts without multimorbidity, indicating that the risk associated with foreign birth was amplified in the presence of multiple chronic conditions. Conversely, foreign-born individuals without multimorbidity exhibited lower relative odds (ROR = 0.51, 95% CI: 0.30–0.84, P = 0.014). Similarly, the association between smoking and prediabetes varied depending on multimorbidity status during the pandemic. Among individuals without multimorbidity, current smoking was linked to a significantly stronger positive association with prediabetes (ROR = 2.31, 95% CI: 1.41–3.78, P = 0.003) compared to the reference group. In contrast, and counterintuitively, among individuals with multimorbidity, current smoking was associated with a significant reduction in its risk effect (ROR = 0.31, 95% CI: 0.14–0.66, P = 0.006).

Sensitivity analyzes using multiply imputed data confirmed the robustness of our primary findings (Table S5 and Table S6). The key interactions between sex, pandemic period, and sociodemographic and behavioral factors remained consistent. Notably, the protective effect of higher education against type 2 diabetes and the shifting risk associated with marital status were replicated. The differential effect modification by depressive symptoms—where it attenuated behavioral risks for prediabetes but amplified them for type 2 diabetes—was also upheld. Furthermore, these analyzes provided increased precision, reinforcing that the protective effect of high income was primarily driven by women and that the sex-specific protective effect of sleep was a pre-pandemic phenomenon. The stability of these complex interactions across analytical methods underscores the reliability of our conclusions about the dynamic nature of diabetes determinants across the pandemic.

## Discussion

This study offers a conceptual synthesis of how social, psychological, and lifestyle determinants interact to shape dysglycemia risk in the context of a major societal disruption. This integrative perspective is grounded in the social determinants of diabetes framework, which emphasizes the interdependence of structural resources, psychosocial stressors, and health behaviors in shaping metabolic risk. [7, 12]. Our findings underscore that these domains are not independent; rather, they operate through interconnected pathways—such as socioeconomic resources influencing health behaviors and psychological distress modulating behavioral risks—that were differentially impacted by the pandemic.

Recently, Inoue et al. reported a modest but statistically insignificant decline in the prevalence of type 2 diabetes among US adults during the COVID-19 pandemic [18]. In contrast, our study observed a statistically significant reduction, from 13.9% to 11.3%, most pronounced among adults aged ≥ 60 years. Concurrently, prediabetes increased substantially in the same age group. Several system-level and methodological explanations merit consideration. First, reduced access to routine screenings and diagnostic services may have contributed to underdetection of undiagnosed diabetes [29]. Second, excess mortality in older adults—particularly those with severe COVID-19 or existing glycemic disorders—may have altered population composition [30–32]. Third, exclusion of individuals with missing social or behavioral data may have inadvertently enriched the analytic sample with higher socioeconomic strata [33]. Together, these factors likely contributed to the divergence between our findings and previous national estimates.

Within this altered landscape, our findings reveal a dynamic reconfiguration of established social gradients in glycemic outcomes, highlighting the interplay between structural resources and gendered contexts. The protective role of education aligns with longstanding evidence linking higher educational attainment to improved metabolic outcomes through enhanced health literacy, occupational advantages, and healthier environments. In contrast, the sex-specific reversal we observed does not contradict the pre-pandemic literature but rather extends it. Prior evidence consistently shows that higher income is generally associated with lower diabetes risk, yet this protective effect varies by factors such as age, sex, and physical activity [34–36]. Our findings build on this non-uniform pattern by demonstrating that, under pandemic conditions, income became metabolically protective for women but a potential liability for men. These divergent associations may reflect the complex interplay between socioeconomic status and gendered contexts, where the same structural resource (high income) translates into different psychosocial burdens and behavioral patterns for men and women. For men, high income and occupational status may be linked to increased metabolic risk due to social and occupational pressures, such as long working hours, sedentary work, or stress-related overeating [37]. In contrast, for women, higher income may provide better access to health resources, knowledge, and environments that support healthier lifestyles [38]. Therefore, income does not have a uniform effect on metabolic risk but interacts with sex-specific social contexts to shape differential susceptibility.

Prior research consistently identifies marriage as protective for cardiometabolic health, primarily through mechanisms involving shared routines, social support, and pooled household resources [39]. However, our finding that married and divorced older adults lost their pre-pandemic protection against prediabetes aligns with emerging longitudinal analyzes showing increased loneliness, disrupted social ties, and declining engagement with preventive healthcare among older adults during lockdowns [40]. Our study, therefore, extends existing literature by demonstrating that relationship status—usually considered a stable determinant—became sensitive to macro-level constraints, highlighting that social support mechanisms may be more fragile during crises than previously assumed. Before the pandemic, foreign-born individuals consistently exhibited higher vulnerability to metabolic risk due to structural barriers such as limited healthcare access, acculturation stress, and constrained dietary environments [41]. However, our finding of disappearing nativity-related disparities during the pandemic diverges from these well-established patterns. This shift may be attributable to broader changes in dietary and social behaviors during the lockdowns, which potentially narrowed cultural and environmental gaps. Beyond individual determinants, our findings underscore the social significance of dysglycemia risk during periods of societal disruption. The shifting associations of income, marital status, and nativity with metabolic outcomes reflect structural processes—such as economic inequality, social exclusion, and unequal access to institutional support—that were magnified during the pandemic. The disappearance of nativity-related disparities and the erosion of marital-status protection among older adults suggest that longstanding vulnerabilities were restructured under crisis conditions. These patterns highlight how structural constraints and social position influenced individuals’ ability to maintain health-promoting behaviors, emphasizing the need to view dysglycemia risk within the broader context of inequality rather than attributing it solely to personal choices.

The most compelling evidence for the interconnected nature of our framework comes from the differential moderating role of depressive symptoms, which sits at the nexus of psychosocial distress and behavioral regulation. Depressive symptoms were assessed using the PHQ-9, a validated and widely used instrument for population-based screening of depression severity [42]. Prior literature identifies depression as a metabolic risk enhancer through neuroendocrine dysregulation, inflammation, impaired adherence, and unhealthy coping behaviors [9]. While our results support this pattern for type 2 diabetes—where depression synergistically amplified the harmful effects of alcohol and smoking—they reveal an opposite dynamic in prediabetes, where depressive symptoms attenuated risk-enhancing behaviors during the pandemic. Descriptive patterns in Table S7 reinforce this: among individuals with prediabetes and elevated depressive symptoms, alcohol intake and smoking both declined; in contrast, individuals with diabetes and depressive symptoms experienced substantial increases in these behaviors. These divergent pathways suggest that depression may induce behavioral withdrawal or increased health-seeking in early dysglycemia, but exacerbate metabolic harm in established diabetes due to greater physiological burden and impaired self-management [43, 44]. Given the cross-sectional design, reverse causality cannot be ruled out. It is plausible that individuals with diabetes may experience increased psychological distress due to the disease burden, lifestyle restrictions, or financial strain, which could potentially exaggerate the observed associations between depressive symptoms and glycemic status. Longitudinal studies are needed to disentangle these bidirectional relationships and establish clearer temporal sequencing.

Lifestyle determinants, the most immediate factors in our framework, also exhibited distinct patterns that reflect broader social and psychosocial disruptions upstream. Physical activity is strongly protective against type 2 diabetes in older adults in our study, reinforcing robust evidence from large aging cohorts that demonstrates how movement attenuates age-related metabolic decline [45]. However, the diminished protective effect among young adults during the pandemic aligns closely with accumulating reports of reduced structured physical activity, the closure of exercise facilities, and ongoing lifestyle instability [46, 47]. There is substantial evidence linking shorter sleep duration to an increased risk of prediabetes and diabetes [48, 49]. Sleep deprivation can induce insulin resistance through various metabolic pathways, including reduced glucose tolerance, altered hormone secretion (such as elevated cortisol and changes in leptin and ghrelin levels), and impaired insulin signaling in adipose tissue [50]. Studies during the COVID-19 lockdowns found that while people slept more overall, they experienced less deep sleep and poorer sleep quality [51]. Pandemic-specific factors, such as predisposing vulnerabilities, stress from health fears, social isolation, and perpetuating behaviors like irregular sleep schedules, may have contributed to this decline in sleep quality [52]. In our study, sleep duration was protective against prediabetes in men before the pandemic, but this effect disappeared in the post-pandemic period. This suggests that promoting adequate sleep duration alone may be insufficient to mitigate risk if sleep quality and circadian rhythm integrity are not also addressed. Age-stratified analyzes further revealed distinct protective sleep patterns during the pandemic: weekend sleep of ≥ 8 h was associated with a markedly lower risk of type 2 diabetes among younger adults, whereas weekday sleep of ≥ 8 h conferred protection for middle-aged adults. These findings suggest that the metabolic benefits of sleep are influenced by both developmental stage and weekly rhythms, with weekend "catch-up" sleep particularly important for younger adults [53], while consistent weekday sleep remains crucial for middle-aged populations [54].

Dietary patterns, another key behavioral domain, further illustrate sharp differences across age groups, socioeconomic strata, and household contexts. The DII has been extensively validated as a literature-derived, population-based measure of dietary inflammatory potential, with robust construct validity and reproducible associations with systemic inflammatory markers such as C-reactive protein [55–57]. In our study, higher DII scores emerged as a significant risk factor for type 2 diabetes among young adults during the pandemic. This pattern is consistent with pre-pandemic evidence indicating that younger adults and women tend to exhibit stronger metabolic sensitivity to pro-inflammatory diets [58]. This shift may reflect the unique vulnerabilities of younger adults to pandemic-related dietary changes, including increased reliance on processed and delivery foods, along with the disruption of established eating patterns [59]. However, our findings that pre-pandemic pro-inflammatory diets were paradoxically associated with lower prediabetes risk among older adults suggest that such diets may have been counterbalanced by other protective factors in middle-aged and older populations before the societal disruption. This age-specific reversal highlights that the impact of behavioral risk factors is not static; rather, it can be dramatically altered when the surrounding social and environmental context—such as food access, work patterns, and economic stress—is abruptly changed. There is a substantial and growing body of research utilizing the CDAI, which broadly supports its role as a stable, nutrient-based indicator of cumulative antioxidant intake [60–62]. In our study, the enhanced protective association of higher antioxidant intake (CDAI) among middle-aged and older adults during the pandemic underscores the potential role of diet quality as a resilience factor in the face of chronic stress and immune challenges. The COVID-19 pandemic was marked by sustained inflammatory activation, oxidative stress, immune dysregulation, and psychosocial strain, all of which disproportionately impacted metabolic homeostasis in older adults, whose mitochondrial reserves, β-cell compensatory capacity, and antioxidant defenses are already diminished [63, 64]. Within this framework, a higher dietary intake of antioxidants may have provided a buffering effect against stress-induced metabolic deterioration specifically among adults aged 40 and older, where physiological redundancy is limited and cumulative oxidative damage is more pronounced. Notably, the lack of a similar protective effect in younger adults supports the notion that the metabolic significance of dietary antioxidant exposure is contingent upon both life stage and environmental context, rather than reflecting a static or universally protective nutritional effect. This finding complements the DII results, indicating that during a systemic crisis, dietary risks associated with inflammation may become evident earlier in life, while dietary protections from antioxidants may take on critical importance in later life stages, when physiological resilience is diminished.

Comorbid conditions, including obesity, hypertension, and multimorbidity, not only exerted robust independent effects across all glycemic outcomes but also served as critical modifiers of the influence of social, psychosocial, and behavioral determinants. Hypertension’s stable association with type 2 diabetes across periods and subgroups underscores its well-established link to insulin resistance, endothelial dysfunction, and cardiometabolic clustering [65, 66] In contrast, obesity exhibited significant effect heterogeneity: it was strongly associated with prediabetes in younger adults, but more weakly or inconsistently in older adults, while remaining a consistent and powerful risk factor for type 2 diabetes across all age groups. Research on children and adolescents with obesity also shows strong links between excess adiposity, prediabetes, and progression to type 2 diabetes, with many youth reverting to normoglycemia when their weight stabilizes or decreases. This supports the notion that, in younger individuals, adiposity-driven insulin resistance plays a particularly dominant role in prediabetes risk [67]. In contrast, in older adults, transitions from normal glycemia to prediabetes may be driven less by absolute adiposity and more by qualitative changes in muscle mass, fat distribution, and declining β-cell compensation [68, 69]. The observed increase in type 2 diabetes risk among foreign-born individuals with multimorbidity underscores the synergistic interplay between structural disadvantages, chronic disease burdens, and potentially limited access to healthcare. This supports the concept of syndemic interactions, where social and biological vulnerabilities converge to exacerbate metabolic risk. Mechanistically, individuals with multiple chronic conditions may experience heightened systemic inflammation, increased insulin resistance, and a greater psychological and self-management burden, all of which can amplify the detrimental effects of socioeconomic or behavioral risk factors [70]. Conversely, the counterintuitive association of current smoking with reduced prediabetes risk among participants with multimorbidity during the pandemic may reflect complex behavioral adaptations, such as increased adherence to other health-promoting behaviors, survivorship bias, or compensatory metabolic changes, while also highlighting the limitations inherent in cross-sectional analyses. These findings diverge from prior longitudinal cohort studies that predominantly report uniformly elevated metabolic risk among smokers or individuals with multimorbidity [71]. This suggests that societal disruptions, such as the COVID-19 pandemic, may temporarily reshape the interactions between behavior and metabolic outcomes.

In contrast to the modest decline in diabetes prevalence observed in the present US analysis, several international studies have reported opposing trends, underscoring the heterogeneous impact of the pandemic across different regions. For instance, research from Italy revealed a significant rise in new-onset type 2 diabetes and deteriorating glycemic control, factors partly driven by reduced physical activity and disruptions in diabetes care [19, 72]. Pan-European surveys further highlighted widespread disruptions to diabetes services, increased psychological distress among patients, and a growing dependence on remote care, reflecting the considerable strain on diabetes management throughout Europe [73]. On a global scale, experts have stressed the importance of coordinated recovery strategies to reinstate screening, monitoring, and educational services that were curtailed during the pandemic [16]. In contrast, the relative decline in diabetes prevalence observed in our US sample may be partially attributed to differences in population structure, disease detection timelines, and behavioral adaptations during the recovery phase. Notably, studies from both the United States and South Korea have reported rising diabetes incidence in younger age groups, suggesting that the metabolic effects of the pandemic were age- and context-dependent [74, 75]. Together, these international findings highlight that, although the direction of epidemiological changes varies, the COVID-19 pandemic led to significant shifts in the behavioral, psychosocial, and healthcare determinants of metabolic health.

The present findings have direct relevance for public policy, health service delivery, and population-level communication strategies. First, several observed social gradients—such as the stable protective effect of education, sex-specific income patterns, and the loss of marital-status protection in older adults—highlight the need for diabetes prevention efforts to be explicitly embedded within structural policies targeting economic security, social vulnerability, and health literacy. These results align with the CDC’s Social Determinants of Health Framework and suggest that diabetes prevention programs—such as the National Diabetes Prevention Program (DPP)—should incorporate sex- and age-stratified recruitment strategies. Enhanced outreach should be directed at older adults living alone, high-income men exposed to occupational stress, and immigrant women, whose pre-pandemic disadvantages reemerged under structural strain. Second, the divergent moderating effects of depressive symptoms across glycemic stages underscore the urgent need for integrated behavioral and mental health services within primary care and endocrinology settings. Specifically, our findings support reimbursement policies that formally integrate PHQ-9 screening into routine diabetes visits, care coordination between mental health professionals and diabetes educators, and expanded telehealth infrastructure to ensure continuity of psychological support during societal disruptions. For individuals with type 2 diabetes, depression amplified the harmful metabolic effects of smoking and alcohol use, suggesting that behavioral counseling and digital cessation tools should be incorporated into diabetes management pathways. For individuals with prediabetes, depressive symptoms attenuated lifestyle risk, indicating that early identification and psychosocial stabilization could prevent the transition to overt disease. Third, the observed changes in sleep patterns, dietary inflammatory burden, and physical activity across age groups emphasize the need for crisis-responsive, demographically tailored health promotion strategies. For younger adults, policies should prioritize weekend sleep regularization, regulate access to ultra-processed foods during emergencies, and strengthen digital platforms that facilitate physical activity when in-person settings are disrupted. For middle-aged adults, consistent weekday sleep and protected work–life boundaries should be emphasized through employer-level health promotion programs and labor regulations that mitigate sedentary stress associated with high-income jobs. For older adults, sustained access to safe physical activity opportunities, nutrition support programs, and social network interventions will be essential to counteract pandemic-related functional decline. Finally, the strong age- and sex-specific interaction patterns underscore the importance of communication strategies that move beyond one-size-fits-all public messaging. Health agencies should adopt segmented communication approaches that reflect differential behavioral vulnerabilities—for example, emphasizing circadian regularity in middle-aged adults, behavioral risk amplification in depressed individuals with established diabetes, and dietary inflammatory risk among young adults during crises. Together, these implications suggest that dysglycemia prevention must be situated within a multisectoral, context-sensitive policy framework that integrates primary care, mental health services, social support, community networks, and targeted communication to sustain metabolic health during and beyond periods of societal disruption.

Although this study employed validated and widely used indicators, each tool carries conceptual and methodological limitations. The PHQ-9, while sensitive to acute emotional distress, may capture short-term pandemic-related reactivity rather than chronic psychological burden. The DII, which reflects nutrient-level inflammatory potential, is constrained by single-day recall and may underestimate structural dietary constraints or the increased consumption of ultra-processed foods during societal disruptions. The CDAI similarly measures antioxidant nutrient intake but does not account for broader dietary patterns or changes in the food environment, limiting its sensitivity to pandemic-related shifts. Self-reported data on sleep, alcohol, and tobacco use are subject to differential reporting biases based on sex and age, which may attenuate interaction estimates. These considerations highlight that, while suitable for population surveillance, the selected indicators capture only a portion of the multidimensional behavioral and psychosocial complexity underlying dysglycemia, emphasizing the need for objective and longitudinal measures in future studies.

Despite its strengths, including a nationally representative sample, robust statistical modeling, and pre- versus post-pandemic comparisons, this study has several limitations. Its cross-sectional design prevents definitive causal conclusions, and the reliance on self-reported measures of lifestyle and psychosocial factors may introduce reporting bias. Although the PHQ-9 is a validated instrument for assessing depressive symptoms, its self-reported nature may be subject to response bias, especially during the pandemic when acute stressors could have temporarily heightened symptom reporting. Future studies using clinical diagnoses or longitudinal psychological assessments would offer greater diagnostic precision. Additionally, while our primary analysis relied on complete-case data—a recognized limitation—we conducted a rigorous sensitivity analysis using multiple imputation, which confirmed the robustness of our core conclusions. The differences observed for certain variables (e.g., sleep duration) underscore the complexity of missing data mechanisms and demonstrate that our key findings are based on associations consistent across both analytical approaches. Moreover, residual confounding from unmeasured variables, such as regional pandemic effects, occupational exposures, or diet composition, remains a possibility. Selection bias also warrants explicit consideration. Missingness in NHANES is not random: individuals with lower income, poorer mental health, and more unstable lifestyle patterns are more likely to have incomplete psychosocial or behavioral data. As a result, complete-case analysis may disproportionately retain participants with higher socioeconomic stability, potentially attenuating associations between adverse psychosocial exposures and dysglycemia. Although multiple imputation analyzes yielded broadly consistent results, the modest differences observed in sleep duration and depressive symptoms suggest that excluding participants with greater vulnerability may bias interaction estimates toward the null. Therefore, our findings likely represent conservative estimates of the true magnitude of social and psychosocial disparities.

Building on these findings, a clearer research agenda is essential. First, longitudinal cohort studies are needed to explicitly test the temporal sequencing suggested by our results—for example, whether depressive symptoms drive glycemic deterioration in individuals with diabetes but moderate risk in those with prediabetes, and whether the pandemic accelerated these divergent pathways. Second, future research should explore the mechanisms underlying the stable sex differences observed across cycles, particularly focusing on the structural, occupational, and psychosocial processes that may explain why income and lifestyle behaviors operate differently in men and women. Third, studies should evaluate natural experiments and policy adaptations introduced during and after the pandemic—such as the expansion of telemedicine, the integration of mental health care in primary care, and changes in food assistance programs—to assess their causal impact on metabolic trajectories. Fourth, mechanistic and behavioral studies are needed to examine how stress-related neuroendocrine pathways, dietary shifts, and sleep disturbances interact to influence dysglycemia during periods of societal disruption. Finally, the development and validation of risk-stratified, sex-specific, and context-responsive intervention strategies should be prioritized to translate these findings into actionable public health practice.

## Conclusions

This nationally representative study suggests that the COVID-19 pandemic was associated with significant shifts in the social, psychological, and behavioral factors related to prediabetes and type 2 diabetes among US adults, revealing risk patterns highly specific to sex, age, and disease stage. By employing an integrated conceptual framework, we demonstrate how the pandemic’s disruption acted through interconnected pathways: altering structural and socioeconomic conditions (e.g., income gradients, marital support), which in turn influenced psychosocial distress (depressive symptoms), and subsequently modulated health behaviors (diet, sleep, substance use) and their metabolic consequences. Crucially, these pathways were differentially activated by sex, age, and disease stage, revealing that societal crises do not affect metabolic risk uniformly but reconfigure it along pre-existing demographic and clinical fault lines. While overall type 2 diabetes prevalence declined, the growth in prediabetes—particularly among older adults—and the heightened psychological burden signal emerging long-term risks. Education consistently conferred protection across periods, whereas income, marital status, and nativity displayed shifting associations that reflect the broader social reorganization triggered by the pandemic. The markedly different moderating effects of depressive symptoms on alcohol and tobacco use across glycemic stages highlight the need to embed mental health within metabolic risk frameworks. These results emphasize that diabetes prevention and management must extend beyond individual lifestyle advice to adopt socially informed, psychologically integrative, and sex-sensitive approaches. Our findings translate into clear, actionable implications across three domains: 1. For Clinical Practice: Integrate routine depression screening (e.g., PHQ‑9) into diabetes and prediabetes care. Tailor behavioral counseling to disease stage—prioritizing integrated cessation support for patients with type 2 diabetes and depression, and early psychosocial stabilization for those with prediabetes and depression. 2. For Public Health Policy: Design interventions that are sex‑responsive and age‑specific. Key targets include mitigating work‑related stress and sedentarism in high‑income men, addressing dietary inflammation in younger adults, and strengthening social support and access to preventive services for older adults and immigrant communities. 3. For Health Surveillance and Communication: Incorporate indicators of social, psychological, and behavioral well‑being into metabolic health monitoring. Public health messaging should move beyond generic advice to segment communication (e.g., highlighting sleep regularity for middle‑aged adults, dietary risks for young adults during crises). Ethical considerations are central: the groups most affected by changing risk pathways (older adults, low-income individuals, and immigrant communities) are those least equipped to buffer structural shocks, underscoring the societal responsibility to anticipate and mitigate crisis-related metabolic harms.

This study makes several distinct contributions. It provides the first integrated post-pandemic assessment of how social, psychological, and behavioral determinants jointly reorganized across glycemic stages; identifies age- and sex-specific interaction patterns that challenge assumptions of uniform behavioral risk; and demonstrates that depressive symptoms can amplify or attenuate lifestyle–glycemia relationships depending on disease stage. At the same time, caution is warranted in interpreting complex interaction terms, which are exploratory and may be susceptible to residual confounding or multiple comparison effects. Replication in longitudinal datasets is necessary to differentiate stable causal pathways from transient pandemic-related dynamics.

Future research should prioritize longitudinal cohort studies to establish temporal ordering, quasi-experimental evaluations of policy and service adaptations introduced during the pandemic, and mechanistic investigations of stress-related neuroendocrine pathways linking psychosocial burden and metabolic change. Validation of psychosocial and dietary indicators in crisis contexts, as well as the development of risk-stratified and context-sensitive interventions, is also essential to refine predictive models of dysglycemia. Finally, although NHANES provides national representativeness, variations in cultural norms, structural vulnerabilities, health system capacity, and pandemic severity may constrain generalizability to other settings, highlighting the need for comparative international research.

Overall, this study contributes a multidomain framework that advances theoretical understanding and provides actionable insights for clinicians, policymakers, and public health practitioners preparing for the long-term metabolic consequences of global crises.

## Supplementary Information

Additional file1

## Data Availability

Publicly available datasets were analyzed in this study. This data can be found here: https://www.cdc.gov/nchs/nhanes/.

## Abbreviations

BMI: Body mass index
CDAI: Composite dietary antioxidant index
CVD: Cardiovascular disease
DII: Dietary inflammatory index
FPG: Fasting plasma glucose
HbA1c: Hemoglobin A1c
NCHS: National center for health statistics
NHANES: National health and nutrition examination survey
OR: Odds ratio
PHQ-9: Patient health questionnaire-9
PIR: Poverty-income ratio
ROR: Ratio of odds ratios
SES: Socioeconomic status
WC: Waist circumference
WHO: World health Organization
