# Supplementary material for: Social, psychosocial, and lifestyle determinants of diabetes and prediabetes in US adults before and after COVID-19: a cross-sectional NHANES analysis
Source: Diabetol Metab Syndr. 2026 Jan 31;18:70. doi: 10.1186/s13098-026-02100-8 (PMC12934095; doi:10.1186/s13098-026-02100-8)
Supplement: Supplementary file 1 — Additional file1 [file 13098_2026_2100_MOESM1_ESM.docx]

**Figure S1.** Flowchart for selection of study population


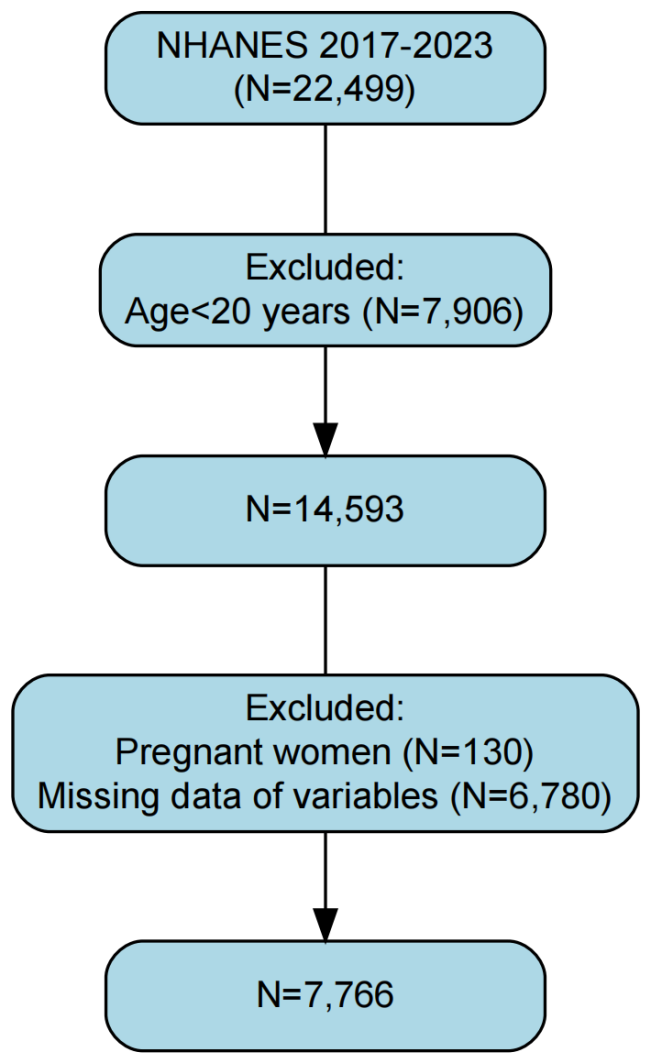


**Table S1.** Age-adjusted prevalence of normoglycemia, prediabetes and T2D pre- (2017-2020) and post- (2021-2023) COVID-19 pandemic.

| Group | Glycemic Status | Pre-pandemic (2017-2020) | Post-pandemic (2021-2023) | P-value |
| --- | --- | --- | --- | --- |
| Overall | Normoglycemia | 51.5 (49.1, 53.8) [n=2,000] | 54.3 (50.9, 57.6) [n=1,364] | 0.002 |
|  | Prediabetes | 34.7 (32.3, 37.1) [n=1,804] | 34.5 (31.4, 37.5) [n=1,177] | 0.218 |
|  | T2D | 13.9 (12.9, 14.8) [n=964] | 11.3 (10.1, 12.5) [n=457] | <0.001 |
| Men | Normoglycemia | 46.6 (43.7, 49.5) [n=883] | 50.6 (46.9, 54.4) [n=555] | 0.013 |
|  | Prediabetes | 37.8 (35.2, 40.3) [n=947] | 36.2 (32.2, 40.3) [n=540] | 0.848 |
|  | T2D | 15.7 (13.7, 17.6) [n=521] | 13.1 (11.3, 15.0) [n=233] | 0.001 |
| Women | Normoglycemia | 56.1 (52.6, 59.6) [n=1,117] | 57.6 (54.0, 61.2) [n=809] | 0.17 |
|  | Prediabetes | 31.6 (28.2, 35.0) [n=857] | 32.8 (29.5, 36.0) [n=637] | 0.085 |
|  | T2D | 12.3 (10.8, 13.8) [n=443] | 9.7 (7.9, 11.4) [n=224] | <0.001 |
| Age 20-39 | Normoglycemia | 69.3 (65.3, 73.4) [n=963] | 75.2 (70.9, 79.6) [n=505] | <0.001 |
|  | Prediabetes | 27.1 (23.2, 31.0) [n=409] | 22.0 (18.9, 25.1) [n=151] | 0.003 |
|  | T2D | 3.6 (2.7, 4.5) [n=59] | 2.8 (1.0, 4.6) [n=21] | 0.306 |
| Age 40-59 | Normoglycemia | 46.0 (43.2, 48.8) [n=626] | 45.2 (40.6, 49.8) [n=386] | 0.001 |
|  | Prediabetes | 37.8 (34.2, 41.5) [n=674] | 40.2 (35.3, 45.1) [n=331] | 0.233 |
|  | T2D | 16.2 (13.5, 18.9) [n=318] | 14.6 (11.7, 17.4) [n=130] | 0.01 |
| Age ≥60 | Normoglycemia | 29.7 (26.5, 32.8) [n=411] | 32.9 (28.7, 37.0) [n=473] | <0.001 |
|  | Prediabetes | 42.5 (38.8, 46.2) [n=721] | 46.6 (43.0, 50.2) [n=695] | 0.004 |
|  | T2D | 27.8 (25.4, 30.2) [n=587] | 20.6 (17.7, 23.4) [n=306] | <0.001 |

T2D, type 2 diabetes.

**Table S2.** Association of social, psychosocial, and lifestyle determinants with prediabetes and type 2 diabetes before and after the COVID-19 pandemic, stratified by age.

| Age | Variables |  | Prediabetes | | | | T2D | | | |  |  |
| --- | --- | --- | --- | --- | --- | --- | --- | --- | --- | --- | --- | --- |
|  |  |  | 2017-2020 | | 2021-2023 | | 2017-2020 | | 2021-2023 | |  |  |
|  |  |  | OR （95%CI） | P-value | OR （95%CI） | P-value | OR （95%CI） | P-value | OR （95%CI） | P-value |  |  |
| 20-39 | Educational levels | Less than college | Reference |  | Reference |  | Reference |  | Reference |  |  |  |
|  |  | Some college | 1.15 (0.72-1.82) | 0.570 | 1.12 (0.54-2.35) | 0.766 | 0.62 (0.37-1.06) | 0.095 | 0.99 (0.45-2.21) | 0.987 |  |  |
|  |  | College graduate or above | 1.46 (0.84-2.55) | 0.198 | 0.57 (0.21-1.53) | 0.300 | 0.50 (0.18-1.42) | 0.210 | 0.30 (0.09-1.02) | 0.090 |  |  |
|  | Married status | Never married | Reference |  | Reference |  | Reference |  | Reference |  |  |  |
|  |  | Divorced, separated or widowed | 2.44 (1.36-4.36) | 0.008 | 2.61 (1.43-4.75) | 0.014 | 0.84 (0.11-6.34) | 0.867 | 0.00 (0.00-0.00) | <0.001 |  |  |
|  |  | Married or living with partner | 1.86 (1.31-2.64) | 0.003 | 1.21 (0.79-1.85) | 0.409 | 1.29 (0.68-2.43) | 0.446 | 1.14 (0.44-2.98) | 0.795 |  |  |
|  | Place of birth | US-born | Reference |  | Reference |  | Reference |  | Reference |  |  |  |
|  |  | Born outside the US | 0.91 (0.53-1.56) | 0.731 | 1.00 (0.65-1.53) | 0.986 | 4.35 (1.43-13.26) | 0.018 | 0.28 (0.06-1.24) | 0.128 |  |  |
|  | PIR levels | Low income | Reference |  | Reference |  | Reference |  | Reference |  |  |  |
|  |  | Middle income | 1.30 (0.84-2.01) | 0.249 | 0.76 (0.47-1.23) | 0.293 | 0.83 (0.45-1.51) | 0.545 | 0.97 (0.42-2.25) | 0.948 |  |  |
|  |  | High income | 1.33 (0.83-2.12) | 0.251 | 1.08 (0.50-2.33) | 0.849 | 0.60 (0.20-1.81) | 0.375 | 0.19 (0.04-0.84) | 0.060 |  |  |
|  | Work | Not-employed | Reference |  | Reference |  | Reference |  | Reference |  |  |  |
|  |  | Part-time employee | 0.86 (0.55-1.36) | 0.528 | 0.73 (0.32-1.65) | 0.473 | 0.56 (0.23-1.38) | 0.224 | 0.92 (0.21-3.96) | 0.911 |  |  |
|  |  | Full-time employee | 1.39 (0.88-2.20) | 0.178 | 0.71 (0.43-1.17) | 0.212 | 0.38 (0.16-0.87) | 0.035 | 0.79 (0.17-3.67) | 0.775 |  |  |
|  | Health insurance | No | Reference |  | Reference |  | Reference |  | Reference |  |  |  |
|  |  | Yes | 1.54 (1.02-2.34) | 0.056 | 1.36 (0.81-2.29) | 0.269 | 1.01 (0.46-2.20) | 0.985 | 0.68 (0.17-2.77) | 0.605 |  |  |
|  | Depression levels | No/minimal depression | Reference |  | Reference |  | Reference |  | Reference |  |  |  |
|  |  | Depression-symptoms | 1.32 (0.86-2.03) | 0.212 | 1.00 (0.70-1.42) | 0.988 | 1.34 (0.78-2.30) | 0.310 | 1.55 (0.68-3.53) | 0.320 |  |  |
|  | Smoking status | Never | Reference |  | Reference |  | Reference |  | Reference |  |  |  |
|  |  | Ex-smoker | 1.68 (0.95-2.96) | 0.090 | 1.01 (0.53-1.93) | 0.980 | 1.42 (0.46-4.33) | 0.550 | 3.24 (1.23-8.55) | 0.045 |  |  |
|  |  | Current smoker | 1.18 (0.88-1.59) | 0.270 | 2.00 (1.36-2.94) | 0.008 | 0.84 (0.34-2.10) | 0.717 | 2.24 (0.61-8.18) | 0.257 |  |  |
|  | PA_levels | Low active | Reference |  | Reference |  | Reference |  | Reference |  |  |  |
|  |  | Moderate active | 0.53 (0.27-1.04) | 0.079 | 0.70 (0.35-1.39) | 0.335 | 1.27 (0.26-6.12) | 0.773 | 0.34 (0.07-1.63) | 0.216 |  |  |
|  |  | High active | 0.53 (0.37-0.74) | 0.002 | 0.86 (0.57-1.29) | 0.486 | 0.48 (0.16-1.42) | 0.201 | 0.78 (0.18-3.27) | 0.740 |  |  |
|  | Sleep hours weekdays | <6h | Reference |  | Reference |  | Reference |  | Reference |  |  |  |
|  |  | 6-8h | 0.58 (0.34-1.01) | 0.072 | 1.12 (0.62-2.02) | 0.719 | 1.21 (0.45-3.21) | 0.713 | 0.24 (0.07-0.88) | 0.063 |  |  |
|  |  | ≥8h | 0.51 (0.26-0.99) | 0.063 | 1.31 (0.64-2.68) | 0.484 | 0.66 (0.27-1.67) | 0.395 | 0.59 (0.19-1.88) | 0.399 |  |  |
|  | Sleep hours weekends | <6h | Reference |  | Reference |  | Reference |  | Reference |  |  |  |
|  |  | 6-8h | 1.35 (0.81-2.25) | 0.263 | 1.03 (0.52-2.05) | 0.925 | 1.38 (0.65-2.94) | 0.413 | 1.04 (0.32-3.44) | 0.948 |  |  |
|  |  | ≥8h | 1.01 (0.63-1.61) | 0.973 | 0.99 (0.64-1.53) | 0.971 | 1.03 (0.51-2.09) | 0.932 | 0.16 (0.06-0.41) | 0.005 |  |  |
|  | Energy intake, kcal |  | 0.91 (0.75-1.11) | 0.378 | 1.13 (0.95-1.35) | 0.209 | 1.00 (0.79-1.27) | 0.995 | 0.64 (0.37-1.10) | 0.139 |  |  |
|  | Alcohol_consumption_g_week |  | 0.87 (0.70-1.07) | 0.202 | 0.83 (0.63-1.09) | 0.209 | 0.38 (0.13-1.06) | 0.081 | 0.72 (0.18-2.96) | 0.660 |  |  |
|  | CDAI |  | 0.90 (0.74-1.09) | 0.293 | 1.05 (0.85-1.30) | 0.683 | 1.06 (0.78-1.46) | 0.703 | 0.45 (0.25-0.81) | 0.027 |  |  |
|  | DII |  | 1.25 (1.02-1.52) | 0.041 | 1.00 (0.84-1.20) | 1.000 | 0.84 (0.58-1.22) | 0.368 | 2.10 (1.36-3.26) | 0.009 |  |  |
|  | Hypertension | No | Reference |  | Reference |  | Reference |  | Reference |  |  |  |
|  |  | Yes | 2.49 (1.74-3.56) | <0.001 | 1.48 (0.88-2.48) | 0.170 | 2.73 (1.13-6.59) | 0.038 | 8.84 (3.87-20.18) | 0.001 |  |  |
|  | Obesity | No | Reference |  | Reference |  | Reference |  | Reference |  |  |  |
|  |  | Yes | 2.32 (1.46-3.69) | 0.002 | 1.80 (1.20-2.71) | 0.019 | 4.75 (2.44-9.27) | <0.001 | 15.97 (3.38-75.44) | 0.007 |  |  |
|  | Multimorbidity | No | Reference |  | Reference |  | Reference |  | Reference |  |  |  |
|  |  | Yes | 1.93 (1.36-2.74) | 0.002 | 1.53 (0.98-2.39) | 0.097 | 4.28 (1.99-9.18) | 0.001 | 3.40 (1.40-8.22) | 0.024 |  |  |
| 40-59 | Educational levels | Less than college | Reference |  | Reference |  | Reference |  | Reference |  |  |  |
|  |  | Some college | 1.00 (0.66-1.53) | 0.990 | 0.82 (0.54-1.25) | 0.391 | 0.57 (0.37-0.88) | 0.019 | 1.01 (0.62-1.67) | 0.956 |  |  |
|  |  | College graduate or above | 0.84 (0.57-1.24) | 0.384 | 0.92 (0.58-1.44) | 0.713 | 0.42 (0.25-0.71) | 0.004 | 0.40 (0.21-0.75) | 0.021 |  |  |
|  | Married status | Never married | Reference |  | Reference |  | Reference |  | Reference |  |  |  |
|  |  | Divorced, separated or widowed | 1.89 (1.08-3.33) | 0.039 | 0.99 (0.51-1.91) | 0.973 | 0.82 (0.38-1.74) | 0.604 | 1.34 (0.66-2.73) | 0.441 |  |  |
|  |  | Married or living with partner | 1.02 (0.65-1.60) | 0.939 | 0.77 (0.42-1.39) | 0.412 | 0.92 (0.55-1.54) | 0.755 | 1.00 (0.47-2.12) | 0.998 |  |  |
|  | Place of birth | US-born | Reference |  | Reference |  | Reference |  | Reference |  |  |  |
|  |  | Born outside the US | 1.71 (1.17-2.50) | 0.012 | 1.36 (0.84-2.20) | 0.241 | 0.74 (0.44-1.24) | 0.270 | 0.57 (0.27-1.22) | 0.184 |  |  |
|  | PIR levels | Low income | Reference |  | Reference |  | Reference |  | Reference |  |  |  |
|  |  | Middle income | 1.27 (0.87-1.84) | 0.230 | 0.89 (0.52-1.52) | 0.680 | 0.79 (0.52-1.20) | 0.283 | 1.22 (0.65-2.31) | 0.554 |  |  |
|  |  | High income | 0.83 (0.53-1.28) | 0.409 | 1.14 (0.62-2.09) | 0.681 | 0.67 (0.46-0.98) | 0.052 | 0.49 (0.31-0.78) | 0.017 |  |  |
|  | Work | Not-employed | Reference |  | Reference |  | Reference |  | Reference |  |  |  |
|  |  | Part-time employee | 1.09 (0.54-2.19) | 0.821 | 0.71 (0.47-1.08) | 0.145 | 0.70 (0.35-1.42) | 0.340 | 0.49 (0.17-1.39) | 0.215 |  |  |
|  |  | Full-time employee | 1.07 (0.68-1.68) | 0.776 | 1.01 (0.64-1.59) | 0.964 | 0.83 (0.58-1.18) | 0.317 | 0.50 (0.34-0.74) | 0.009 |  |  |
|  | Health insurance | No | Reference |  | Reference |  | Reference |  | Reference |  |  |  |
|  |  | Yes | 0.76 (0.49-1.18) | 0.241 | 1.14 (0.64-2.02) | 0.671 | 1.43 (0.80-2.55) | 0.237 | 1.77 (0.77-4.09) | 0.213 |  |  |
|  | Depression levels | No/minimal depression | Reference |  | Reference |  | Reference |  | Reference |  |  |  |
|  |  | Depression-symptoms | 1.32 (0.93-1.86) | 0.134 | 1.21 (0.79-1.85) | 0.394 | 1.24 (0.89-1.74) | 0.221 | 2.06 (1.22-3.50) | 0.025 |  |  |
|  | Smoking status | Never | Reference |  | Reference |  | Reference |  | Reference |  |  |  |
|  |  | Ex-smoker | 1.36 (0.86-2.14) | 0.206 | 0.95 (0.56-1.61) | 0.842 | 1.09 (0.70-1.71) | 0.709 | 1.20 (0.51-2.85) | 0.690 |  |  |
|  |  | Current smoker | 1.42 (0.99-2.02) | 0.071 | 1.34 (0.74-2.42) | 0.357 | 0.92 (0.52-1.61) | 0.770 | 1.50 (0.98-2.31) | 0.101 |  |  |
|  | PA_levels | Low active | Reference |  | Reference |  | Reference |  | Reference |  |  |  |
|  |  | Moderate active | 1.29 (0.76-2.21) | 0.362 | 0.82 (0.54-1.24) | 0.370 | 0.85 (0.51-1.41) | 0.540 | 0.65 (0.41-1.03) | 0.103 |  |  |
|  |  | High active | 0.83 (0.61-1.15) | 0.275 | 0.69 (0.51-0.94) | 0.045 | 0.49 (0.30-0.80) | 0.010 | 0.56 (0.34-0.93) | 0.056 |  |  |
|  | Sleep hours weekdays | <6h | Reference |  | Reference |  | Reference |  | Reference |  |  |  |
|  |  | 6-8h | 0.85 (0.53-1.35) | 0.494 | 1.13 (0.70-1.85) | 0.631 | 0.72 (0.42-1.23) | 0.243 | 0.41 (0.21-0.80) | 0.031 |  |  |
|  |  | ≥8h | 0.70 (0.43-1.15) | 0.177 | 1.13 (0.59-2.15) | 0.722 | 0.77 (0.44-1.35) | 0.373 | 0.48 (0.27-0.84) | 0.034 |  |  |
|  | Sleep hours weekends | <6h | Reference |  | Reference |  | Reference |  | Reference |  |  |  |
|  |  | 6-8h | 0.92 (0.61-1.39) | 0.695 | 0.69 (0.48-0.99) | 0.076 | 0.65 (0.34-1.24) | 0.204 | 1.17 (0.51-2.68) | 0.714 |  |  |
|  |  | ≥8h | 0.58 (0.35-0.95) | 0.046 | 0.89 (0.55-1.43) | 0.643 | 0.65 (0.37-1.16) | 0.164 | 0.74 (0.48-1.14) | 0.208 |  |  |
|  | Energy intake, kcal |  | 1.20 (1.00-1.43) | 0.062 | 0.90 (0.74-1.09) | 0.293 | 0.88 (0.68-1.14) | 0.331 | 1.12 (0.94-1.35) | 0.241 |  |  |
|  | Alcohol_consumption_g_week |  | 1.03 (0.88-1.20) | 0.737 | 1.12 (0.95-1.31) | 0.219 | 0.63 (0.34-1.16) | 0.154 | 0.97 (0.74-1.28) | 0.847 |  |  |
|  | CDAI |  | 1.13 (0.92-1.40) | 0.252 | 0.96 (0.80-1.14) | 0.628 | 0.91 (0.74-1.13) | 0.418 | 0.99 (0.82-1.20) | 0.904 |  |  |
|  | DII |  | 0.85 (0.72-1.00) | 0.058 | 1.05 (0.88-1.25) | 0.586 | 1.32 (1.11-1.58) | 0.006 | 1.10 (0.93-1.31) | 0.290 |  |  |
|  | Hypertension | No | Reference |  | Reference |  | Reference |  | Reference |  |  |  |
|  |  | Yes | 1.03 (0.75-1.41) | 0.860 | 0.95 (0.67-1.34) | 0.760 | 4.26 (2.85-6.36) | <0.001 | 3.07 (1.99-4.73) | 0.001 |  |  |
|  | Obesity | No | Reference |  | Reference |  | Reference |  | Reference |  |  |  |
|  |  | Yes | 1.10 (0.88-1.37) | 0.403 | 1.39 (1.00-1.92) | 0.079 | 3.90 (2.52-6.04) | <0.001 | 4.72 (3.05-7.32) | <0.001 |  |  |
|  | Multimorbidity | No | Reference |  | Reference |  | Multimorbidity | No | Reference |  | Reference |  |
|  |  | Yes | 1.17 (0.78-1.77) | 0.457 | 1.08 (0.81-1.45) | 0.612 |  | Yes | 3.69 (2.55-5.34) | <0.001 | 2.54 (1.86-3.48) | <0.001 |
| ≥60 | Educational levels | Less than college | Reference |  | Reference |  | Reference |  | Reference |  |  |  |
|  |  | Some college | 0.84 (0.65-1.09) | 0.209 | 0.94 (0.77-1.14) | 0.559 | 1.12 (0.70-1.81) | 0.645 | 1.01 (0.75-1.34) | 0.965 |  |  |
|  |  | College graduate or above | 1.29 (0.90-1.84) | 0.181 | 1.08 (0.89-1.32) | 0.456 | 0.52 (0.31-0.86) | 0.019 | 0.50 (0.29-0.86) | 0.037 |  |  |
|  | Married status | Never married | Reference |  | Reference |  | Reference |  | Reference |  |  |  |
|  |  | Divorced, separated or widowed | 0.45 (0.24-0.82) | 0.018 | 1.14 (0.71-1.83) | 0.598 | 1.63 (0.70-3.81) | 0.272 | 1.20 (0.70-2.03) | 0.523 |  |  |
|  |  | Married or living with partner | 0.40 (0.20-0.81) | 0.020 | 1.11 (0.67-1.83) | 0.689 | 1.57 (0.70-3.55) | 0.290 | 1.14 (0.67-1.92) | 0.649 |  |  |
|  | Place of birth | US-born | Reference |  | Reference |  | Reference |  | Reference |  |  |  |
|  |  | Born outside the US | 1.62 (1.05-2.49) | 0.041 | 1.15 (0.72-1.84) | 0.574 | 0.67 (0.40-1.12) | 0.141 | 0.80 (0.48-1.35) | 0.429 |  |  |
|  | PIR levels | Low income | Reference |  | Reference |  | Reference |  | Reference |  |  |  |
|  |  | Middle income | 0.97 (0.56-1.70) | 0.923 | 1.63 (1.03-2.59) | 0.072 | 0.99 (0.71-1.36) | 0.929 | 1.05 (0.70-1.59) | 0.805 |  |  |
|  |  | High income | 1.04 (0.57-1.88) | 0.899 | 1.70 (1.09-2.66) | 0.048 | 0.71 (0.42-1.21) | 0.226 | 0.62 (0.38-1.03) | 0.102 |  |  |
|  | Work | Not-employed | Reference |  | Reference |  | Reference |  | Reference |  |  |  |
|  |  | Part-time employee | 1.21 (0.75-1.95) | 0.454 | 1.56 (1.05-2.31) | 0.057 | 0.55 (0.35-0.86) | 0.017 | 0.63 (0.40-0.98) | 0.073 |  |  |
|  |  | Full-time employee | 1.00 (0.71-1.42) | 0.983 | 1.17 (0.89-1.54) | 0.306 | 0.77 (0.50-1.21) | 0.273 | 0.67 (0.45-1.02) | 0.099 |  |  |
|  | Health insurance | No | Reference |  | Reference |  | Reference |  | Reference |  |  |  |
|  |  | Yes | 1.51 (0.79-2.86) | 0.225 | 1.20 (0.64-2.26) | 0.588 | 0.71 (0.34-1.47) | 0.364 | 1.18 (0.45-3.09) | 0.747 |  |  |
|  | Depression levels | No/minimal depression | Reference |  | Reference |  | Reference |  | Reference |  |  |  |
|  |  | Depression-symptoms | 0.66 (0.52-0.84) | 0.003 | 0.87 (0.64-1.19) | 0.419 | 1.84 (1.48-2.28) | <0.001 | 1.48 (0.94-2.33) | 0.129 |  |  |
|  | Smoking status | Never | Reference |  | Reference |  | Reference |  | Reference |  |  |  |
|  |  | Ex-smoker | 0.67 (0.51-0.87) | 0.009 | 1.07 (0.89-1.28) | 0.504 | 1.29 (0.74-2.25) | 0.384 | 1.22 (0.83-1.78) | 0.345 |  |  |
|  |  | Current smoker | 0.99 (0.61-1.60) | 0.958 | 1.51 (1.02-2.22) | 0.071 | 0.81 (0.48-1.36) | 0.437 | 1.03 (0.63-1.68) | 0.918 |  |  |
|  | PA_levels | Low active | Reference |  | Reference |  | Reference |  | Reference |  |  |  |
|  |  | Moderate active | 0.74 (0.50-1.09) | 0.146 | 1.10 (0.86-1.40) | 0.479 | 0.74 (0.41-1.34) | 0.335 | 0.65 (0.45-0.92) | 0.043 |  |  |
|  |  | High active | 1.03 (0.67-1.59) | 0.901 | 1.01 (0.76-1.33) | 0.964 | 0.49 (0.34-0.72) | 0.002 | 0.48 (0.31-0.72) | 0.008 |  |  |
|  | Sleep hours weekdays | <6h | Reference |  | Reference |  | Reference |  | Reference |  |  |  |
|  |  | 6-8h | 0.65 (0.36-1.17) | 0.168 | 0.71 (0.43-1.17) | 0.210 | 1.54 (0.79-3.00) | 0.222 | 1.34 (0.61-2.94) | 0.491 |  |  |
|  |  | ≥8h | 0.57 (0.32-1.01) | 0.071 | 0.66 (0.38-1.15) | 0.184 | 1.30 (0.68-2.47) | 0.440 | 1.65 (0.65-4.22) | 0.326 |  |  |
|  | Sleep hours weekends | <6h | Reference |  | Reference |  | Reference |  | Reference |  |  |  |
|  |  | 6-8h | 1.21 (0.76-1.93) | 0.424 | 1.04 (0.73-1.50) | 0.824 | 0.92 (0.67-1.25) | 0.582 | 0.72 (0.55-0.96) | 0.052 |  |  |
|  |  | ≥8h | 1.18 (0.78-1.80) | 0.438 | 0.89 (0.66-1.21) | 0.483 | 0.83 (0.60-1.14) | 0.265 | 0.79 (0.53-1.17) | 0.273 |  |  |
|  | Energy intake, kcal |  | 1.18 (0.98-1.41) | 0.091 | 1.11 (0.97-1.28) | 0.174 | 0.78 (0.68-0.89) | 0.001 | 0.92 (0.76-1.12) | 0.419 |  |  |
|  | Alcohol_consumption_g_week |  | 0.90 (0.70-1.16) | 0.439 | 1.19 (1.00-1.41) | 0.080 | 0.77 (0.49-1.23) | 0.292 | 0.45 (0.34-0.58) | <0.001 |  |  |
|  | CDAI |  | 1.12 (0.99-1.28) | 0.098 | 1.07 (0.99-1.16) | 0.117 | 0.81 (0.71-0.93) | 0.007 | 0.91 (0.79-1.05) | 0.222 |  |  |
|  | DII |  | 0.83 (0.71-0.98) | 0.045 | 0.96 (0.87-1.06) | 0.456 | 1.27 (1.08-1.51) | 0.011 | 1.19 (1.05-1.34) | 0.026 |  |  |
|  | Hypertension | No | Reference |  | Reference |  | Reference |  | Reference |  |  |  |
|  |  | Yes | 0.82 (0.59-1.14) | 0.254 | 1.14 (0.84-1.54) | 0.433 | 2.96 (2.14-4.10) | <0.001 | 2.09 (1.35-3.24) | 0.009 |  |  |
|  | Obesity | No | Reference |  | Reference |  | Reference |  | Reference |  |  |  |
|  |  | Yes | 0.89 (0.69-1.16) | 0.397 | 1.03 (0.80-1.33) | 0.834 | 3.58 (2.78-4.61) | <0.001 | 2.52 (1.98-3.21) | <0.001 |  |  |
|  | Multimorbidity | No | Reference |  | Reference |  | Reference |  | Reference |  |  |  |
|  |  | Yes | 0.86 (0.64-1.16) | 0.341 | 1.08 (0.84-1.38) | 0.564 | 3.24 (2.37-4.43) | <0.001 | 1.81 (1.29-2.55) | 0.008 |  |  |

Model was adjusted for sex and race. T2D, type 2 diabetes; PIR: poverty income ratio; CDAI: composite dietary antioxidant index; DII: dietary inflammatory index; PA, physical activity.

**Table S3.** Association of social, psychosocial, and lifestyle determinants with prediabetes and type 2 diabetes before and after the COVID-19 pandemic, stratified by sex.

| Sex | Variables |  | Prediabetes | | | | T2D | | | |
| --- | --- | --- | --- | --- | --- | --- | --- | --- | --- | --- |
|  |  |  | 2017-2020 | | 2021-2023 | | 2017-2020 | | 2021-2023 | |
|  |  |  | OR （95%CI） | P-value | OR （95%CI） | P-value | OR （95%CI） | P-value | OR （95%CI） | P-value |
| Men | Educational levels | Less than college | Reference |  | Reference |  | Reference |  | Reference |  |
|  |  | Some college | 1.15 (0.81-1.64) | 0.448 | 0.91 (0.59-1.41) | 0.682 | 0.86 (0.57-1.29) | 0.472 | 1.20 (0.93-1.55) | 0.203 |
|  |  | College graduate or above | 1.35 (0.94-1.94) | 0.121 | 0.81 (0.53-1.24) | 0.364 | 0.53 (0.32-0.87) | 0.021 | 0.52 (0.36-0.77) | 0.010 |
|  | Married status | Never married | Reference |  | Reference |  | Reference |  | Reference |  |
|  |  | Divorced, separated or widowed | 1.55 (0.98-2.45) | 0.078 | 1.16 (0.71-1.88) | 0.576 | 1.07 (0.60-1.91) | 0.812 | 1.99 (1.00-3.97) | 0.085 |
|  |  | Married or living with partner | 1.22 (0.78-1.90) | 0.391 | 1.13 (0.78-1.66) | 0.534 | 1.38 (0.80-2.39) | 0.263 | 1.85 (1.03-3.34) | 0.075 |
|  | Place of birth | US-born | Reference |  | Reference |  | Reference |  | Reference |  |
|  |  | Born outside the US | 1.08 (0.77-1.50) | 0.670 | 1.28 (0.85-1.93) | 0.264 | 0.77 (0.34-1.73) | 0.532 | 0.73 (0.43-1.25) | 0.279 |
|  | PIR levels | Low income | Reference |  | Reference |  | Reference |  | Reference |  |
|  |  | Middle income | 1.14 (0.75-1.72) | 0.557 | 1.22 (0.80-1.86) | 0.375 | 0.88 (0.61-1.25) | 0.475 | 1.40 (0.90-2.17) | 0.173 |
|  |  | High income | 1.17 (0.88-1.55) | 0.284 | 1.54 (1.13-2.08) | 0.024 | 0.79 (0.50-1.25) | 0.323 | 0.71 (0.44-1.17) | 0.216 |
|  | Work | Not-employed | Reference |  | Reference |  | Reference |  | Reference |  |
|  |  | Part-time employee | 1.24 (0.79-1.95) | 0.357 | 1.35 (0.84-2.15) | 0.253 | 0.76 (0.41-1.42) | 0.405 | 0.35 (0.17-0.73) | 0.023 |
|  |  | Full-time employee | 1.36 (0.93-1.99) | 0.129 | 1.21 (0.96-1.52) | 0.151 | 0.83 (0.52-1.33) | 0.446 | 0.74 (0.52-1.06) | 0.138 |
|  | Health insurance | No | Reference |  | Reference |  | Reference |  | Reference |  |
|  |  | Yes | 1.34 (0.94-1.90) | 0.121 | 1.43 (0.83-2.44) | 0.225 | 1.72 (1.12-2.65) | 0.024 | 1.42 (0.61-3.28) | 0.438 |
|  | Depression levels | No/minimal depression | Reference |  | Reference |  | Reference |  | Reference |  |
|  |  | Depression-symptoms | 0.92 (0.68-1.26) | 0.621 | 1.07 (0.76-1.50) | 0.712 | 1.08 (0.74-1.57) | 0.697 | 1.63 (1.04-2.55) | 0.061 |
|  | Smoking status | Never | Reference |  | Reference |  | Reference |  | Reference |  |
|  |  | Ex-smoker | 1.08 (0.76-1.55) | 0.669 | 1.00 (0.71-1.39) | 0.979 | 1.19 (0.75-1.88) | 0.474 | 1.35 (0.86-2.12) | 0.229 |
|  |  | Current smoker | 1.10 (0.83-1.44) | 0.527 | 2.21 (1.53-3.18) | 0.003 | 0.76 (0.51-1.14) | 0.199 | 1.14 (0.70-1.84) | 0.611 |
|  | PA_levels | Low active | Reference |  | Reference |  | Reference |  | Reference |  |
|  |  | Moderate active | 0.86 (0.59-1.25) | 0.429 | 0.95 (0.62-1.47) | 0.833 | 1.15 (0.63-2.10) | 0.648 | 0.63 (0.40-0.99) | 0.081 |
|  |  | High active | 0.86 (0.62-1.20) | 0.389 | 0.85 (0.59-1.20) | 0.378 | 0.59 (0.37-0.93) | 0.035 | 0.55 (0.30-1.02) | 0.092 |
|  | Sleep hours weekdays | <6h | Reference |  | Reference |  | Reference |  | Reference |  |
|  |  | 6-8h | 0.54 (0.37-0.79) | 0.005 | 0.97 (0.67-1.40) | 0.857 | 1.48 (0.95-2.29) | 0.097 | 0.53 (0.25-1.14) | 0.144 |
|  |  | ≥8h | 0.54 (0.33-0.91) | 0.031 | 0.83 (0.47-1.45) | 0.523 | 1.10 (0.62-1.94) | 0.744 | 0.64 (0.30-1.37) | 0.287 |
|  | Sleep hours weekends | <6h | Reference |  | Reference |  | Reference |  | Reference |  |
|  |  | 6-8h | 1.27 (0.88-1.84) | 0.216 | 0.77 (0.42-1.42) | 0.432 | 0.81 (0.50-1.31) | 0.393 | 0.99 (0.59-1.67) | 0.968 |
|  |  | ≥8h | 0.99 (0.71-1.37) | 0.944 | 0.91 (0.55-1.49) | 0.714 | 0.76 (0.44-1.33) | 0.356 | 0.59 (0.38-0.92) | 0.048 |
|  | Energy intake, kcal |  | 1.11 (0.99-1.23) | 0.087 | 0.96 (0.79-1.16) | 0.665 | 0.84 (0.70-1.00) | 0.070 | 1.11 (0.93-1.33) | 0.289 |
|  | Alcohol_consumption_g_week |  | 0.96 (0.86-1.07) | 0.475 | 1.13 (1.00-1.27) | 0.078 | 0.82 (0.56-1.20) | 0.313 | 0.83 (0.55-1.25) | 0.392 |
|  | CDAI |  | 1.05 (0.94-1.18) | 0.400 | 0.93 (0.79-1.11) | 0.465 | 0.93 (0.80-1.08) | 0.379 | 1.03 (0.86-1.23) | 0.736 |
|  | DII |  | 0.91 (0.80-1.03) | 0.164 | 1.04 (0.88-1.23) | 0.654 | 1.23 (1.06-1.42) | 0.012 | 1.11 (0.94-1.30) | 0.246 |
|  | Hypertension | No | Reference |  | Reference |  | Reference |  | Reference |  |
|  |  | Yes | 1.10 (0.87-1.38) | 0.455 | 1.01 (0.69-1.48) | 0.945 | 2.80 (2.03-3.85) | ＜0.001 | 2.52 (1.71-3.72) | 0.001 |
|  | Obesity | No | Reference |  | Reference |  | Reference |  | Reference |  |
|  |  | Yes | 1.16 (0.86-1.56) | 0.354 | 1.19 (0.84-1.68) | 0.348 | 2.80 (1.97-3.99) | ＜0.001 | 3.43 (2.70-4.37) | ＜0.001 |
|  | Multimorbidity | No | Reference |  | Reference |  | Reference |  | Reference |  |
|  |  | Yes | 0.95 (0.69-1.31) | 0.746 | 1.09 (0.83-1.43) | 0.571 | 2.98 (1.84-4.84) | <0.001 | 2.25 (1.82-2.77) | <0.001 |
| Women | Educational levels | Less than college | Reference |  | Reference |  | Reference |  | Reference |  |
|  |  | Some college | 0.86 (0.65-1.16) | 0.344 | 1.04 (0.77-1.40) | 0.805 | 0.77 (0.52-1.12) | 0.190 | 0.85 (0.63-1.15) | 0.320 |
|  |  | College graduate or above | 0.95 (0.75-1.22) | 0.698 | 0.95 (0.70-1.30) | 0.770 | 0.41 (0.25-0.68) | 0.003 | 0.33 (0.19-0.57) | 0.004 |
|  | Married status | Never married | Reference |  | Reference |  | Reference |  | Reference |  |
|  |  | Divorced, separated or widowed | 1.82 (0.95-3.51) | 0.089 | 1.13 (0.76-1.68) | 0.551 | 1.12 (0.70-1.77) | 0.642 | 0.74 (0.44-1.24) | 0.284 |
|  |  | Married or living with partner | 1.44 (0.89-2.32) | 0.157 | 0.96 (0.61-1.50) | 0.855 | 1.11 (0.72-1.71) | 0.638 | 0.68 (0.47-1.00) | 0.084 |
|  | Place of birth | US-born | Reference |  | Reference |  | Reference |  | Reference |  |
|  |  | Born outside the US | 1.54 (1.09-2.18) | 0.025 | 1.15 (0.79-1.65) | 0.484 | 1.24 (0.79-1.95) | 0.364 | 0.65 (0.34-1.23) | 0.218 |
|  | PIR levels | Low income | Reference |  | Reference |  | Reference |  | Reference |  |
|  |  | Middle income | 1.22 (0.90-1.64) | 0.214 | 0.90 (0.66-1.22) | 0.525 | 0.84 (0.62-1.13) | 0.256 | 0.90 (0.61-1.32) | 0.611 |
|  |  | High income | 0.94 (0.70-1.27) | 0.709 | 1.04 (0.76-1.43) | 0.808 | 0.64 (0.45-0.91) | 0.023 | 0.43 (0.27-0.71) | 0.010 |
|  | Work | Not-employed | Reference |  | Reference |  | Reference |  | Reference |  |
|  |  | Part-time employee | 1.02 (0.64-1.64) | 0.929 | 0.98 (0.57-1.70) | 0.955 | 0.68 (0.35-1.30) | 0.257 | 1.09 (0.55-2.16) | 0.806 |
|  |  | Full-time employee | 1.19 (0.88-1.62) | 0.270 | 1.14 (0.81-1.61) | 0.462 | 1.03 (0.75-1.43) | 0.844 | 0.79 (0.60-1.03) | 0.124 |
|  | Health insurance | No | Reference |  | Reference |  | Reference |  | Reference |  |
|  |  | Yes | 0.81 (0.58-1.15) | 0.253 | 0.83 (0.54-1.29) | 0.436 | 0.59 (0.39-0.90) | 0.023 | 1.10 (0.44-2.78) | 0.843 |
|  | Depression levels | No/minimal depression | Reference |  | Reference |  | Reference |  | Reference |  |
|  |  | Depression-symptoms | 1.22 (0.92-1.63) | 0.188 | 0.98 (0.79-1.23) | 0.881 | 2.01 (1.49-2.72) | ＜0.001 | 1.72 (1.27-2.32) | 0.007 |
|  | Smoking status | Never | Reference |  | Reference |  | Reference |  | Reference |  |
|  |  | Ex-smoker | 1.04 (0.82-1.32) | 0.757 | 1.00 (0.80-1.26) | 0.978 | 1.22 (0.68-2.19) | 0.517 | 1.17 (0.77-1.76) | 0.486 |
|  |  | Current smoker | 1.40 (1.09-1.79) | 0.016 | 1.09 (0.68-1.75) | 0.740 | 1.13 (0.61-2.09) | 0.698 | 1.93 (1.15-3.25) | 0.039 |
|  | PA_levels | Low active | Reference |  | Reference |  | Reference |  | Reference |  |
|  |  | Moderate active | 0.88 (0.54-1.42) | 0.601 | 0.83 (0.62-1.10) | 0.233 | 0.59 (0.32-1.07) | 0.099 | 0.57 (0.35-0.92) | 0.049 |
|  |  | High active | 0.63 (0.44-0.91) | 0.024 | 0.85 (0.65-1.13) | 0.296 | 0.37 (0.23-0.59) | ＜0.001 | 0.52 (0.36-0.76) | 0.010 |
|  | Sleep hours weekdays | <6h | Reference |  | Reference |  | Reference |  | Reference |  |
|  |  | 6-8h | 0.98 (0.68-1.40) | 0.908 | 0.91 (0.53-1.57) | 0.753 | 0.73 (0.40-1.33) | 0.323 | 0.73 (0.38-1.42) | 0.383 |
|  |  | ≥8h | 0.68 (0.47-0.98) | 0.055 | 1.03 (0.61-1.74) | 0.927 | 0.64 (0.34-1.20) | 0.184 | 0.83 (0.40-1.74) | 0.638 |
|  | Sleep hours weekends | <6h | Reference |  | Reference |  | Reference |  | Reference |  |
|  |  | 6-8h | 0.94 (0.65-1.34) | 0.723 | 1.03 (0.72-1.46) | 0.887 | 0.86 (0.58-1.28) | 0.471 | 0.77 (0.46-1.29) | 0.347 |
|  |  | ≥8h | 0.73 (0.54-0.97) | 0.046 | 0.90 (0.65-1.24) | 0.534 | 0.72 (0.52-1.01) | 0.070 | 0.81 (0.55-1.19) | 0.318 |
|  | Energy intake, kcal |  | 1.03 (0.86-1.24) | 0.713 | 1.28 (1.04-1.56) | 0.044 | 0.90 (0.69-1.17) | 0.427 | 0.83 (0.63-1.10) | 0.230 |
|  | Alcohol_consumption_g_week |  | 0.89 (0.70-1.12) | 0.330 | 0.99 (0.77-1.26) | 0.912 | 0.22 (0.10-0.46) | ＜0.001 | 0.64 (0.24-1.73) | 0.403 |
|  | CDAI |  | 1.04 (0.87-1.25) | 0.688 | 1.14 (0.97-1.34) | 0.138 | 0.82 (0.70-0.96) | 0.025 | 0.80 (0.65-1.00) | 0.078 |
|  | DII |  | 1.00 (0.83-1.22) | 0.963 | 0.95 (0.84-1.08) | 0.461 | 1.26 (1.09-1.45) | 0.006 | 1.28 (1.04-1.56) | 0.042 |
|  | Hypertension | No | Reference |  | Reference |  | Reference |  | Reference |  |
|  |  | Yes | 1.04 (0.74-1.48) | 0.811 | 1.08 (0.81-1.43) | 0.626 | 4.21 (3.01-5.87) | ＜0.001 | 3.26 (2.26-4.70) | ＜0.001 |
|  | Obesity | No | Reference |  | Reference |  | Reference |  | Reference |  |
|  |  | Yes | 1.43 (1.07-1.92) | 0.027 | 1.49 (1.13-1.96) | 0.020 | 6.18 (4.65-8.20) | ＜0.001 | 4.09 (3.05-5.48) | ＜0.001 |
|  | Multimorbidity | No | Reference |  | Reference |  | Reference |  | Reference |  |
|  |  | Yes | 1.24 (0.95-1.61) | 0.129 | 1.09 (0.83-1.42) | 0.545 | 3.87 (2.84-5.28) | <0.001 | 1.95 (1.37-2.76) | 0.005 |

Model was adjusted for age and race. T2D, type 2 diabetes; PIR: poverty income ratio; CDAI: composite dietary antioxidant index; DII: dietary inflammatory index; PA, physical activity.

**Table S4.** All interaction effects of social, psychosocial, and lifestyle determinants with sex, age, and pandemic period on prediabetes and type 2 diabetes.

| Disease | Interaction term | ROR （95%CI） | β (SE) | P-interaction |
| --- | --- | --- | --- | --- |
| Prediabetes | ≥60 years × less than college × pandemic | 1.22 (0.64-2.30) | 0.20 (0.33) | 0.556 |
|  | ≥60 years × never married × pandemic | 0.50 (0.23-1.09) | -0.68 (0.39) | 0.101 |
|  | ≥60 years × no health insurance × pandemic | 1.88 (0.75-4.74) | 0.63 (0.47) | 0.193 |
|  | ≥60 years × sleep hours (weekdays) <6h × pandemic | 2.69 (0.95-7.60) | 0.99 (0.53) | 0.08 |
|  | ≥60 years × US-born × pandemic | 1.59 (1.07-2.38) | 0.47 (0.20) | 0.032 |
|  | 40-59 years × less than college × pandemic | 1.10 (0.46-2.63) | 0.09 (0.45) | 0.839 |
|  | 40-59 years × never married × pandemic | 1.74 (0.82-3.70) | 0.55 (0.38) | 0.167 |
|  | 40-59 years × no health insurance × pandemic | 0.99 (0.42-2.34) | -0.01 (0.44) | 0.976 |
|  | 40-59 years × sleep hours (weekdays) <6h × pandemic | 2.19 (0.73-6.52) | 0.78 (0.56) | 0.177 |
|  | 40-59 years × US-born × pandemic | 1.41 (1.05-1.90) | 0.34 (0.15) | 0.033 |
|  | alcohol consumption × ≥60 years × pre-pandemic | 1.03 (0.75-1.41) | 0.03 (0.16) | 0.85 |
|  | alcohol consumption × ≥60 years × pandemic | 1.35 (0.87-2.08) | 0.30 (0.22) | 0.189 |
|  | alcohol consumption × 40-59 years × pre-pandemic | 1.17 (0.93-1.46) | 0.15 (0.12) | 0.197 |
|  | alcohol consumption × 40-59 years × pandemic | 1.15 (0.81-1.63) | 0.14 (0.18) | 0.439 |
|  | alcohol consumption × pandemic | 0.93 (0.70-1.25) | -0.07 (0.15) | 0.651 |
|  | born outside the US × ≥60 years × pre-pandemic | 1.20 (0.71-2.05) | 0.18 (0.27) | 0.501 |
|  | born outside the US × ≥60 years × pandemic | 0.91 (0.42-1.96) | -0.10 (0.39) | 0.81 |
|  | born outside the US × 40-59 years × pre-pandemic | 1.41 (0.89-2.23) | 0.34 (0.23) | 0.156 |
|  | born outside the US × 40-59 years × pandemic | 1.05 (0.50-2.21) | 0.05 (0.38) | 0.898 |
|  | born outside the US × 20-39 years × pandemic | 1.00 (0.61-1.63) | -0.00 (0.25) | 1 |
|  | CDAI × ≥60 years × pre-pandemic | 1.25 (1.01-1.55) | 0.22 (0.11) | 0.055 |
|  | CDAI × ≥60 years × pandemic | 0.84 (0.61-1.17) | -0.17 (0.17) | 0.308 |
|  | CDAI × 40-59 years × pre-pandemic | 1.23 (0.98-1.54) | 0.21 (0.12) | 0.091 |
|  | CDAI × 40-59 years × pandemic | 0.79 (0.56-1.11) | -0.24 (0.18) | 0.19 |
|  | CDAI × 20-39 years × pandemic | 1.13 (0.85-1.51) | 0.12 (0.15) | 0.416 |
|  | college graduate or above × ≥60 years × pre-pandemic | 1.05 (0.59-1.86) | 0.05 (0.29) | 0.87 |
|  | college graduate or above × ≥60 years × pandemic | 1.84 (0.70-4.82) | 0.61 (0.49) | 0.229 |
|  | college graduate or above × 40-59 years × pre-pandemic | 0.69 (0.36-1.31) | -0.37 (0.33) | 0.272 |
|  | college graduate or above × 40-59 years × pandemic | 2.45 (0.65-9.21) | 0.90 (0.67) | 0.201 |
|  | college graduate or above × 20-39 years × pandemic | 0.46 (0.16-1.29) | -0.78 (0.53) | 0.159 |
|  | current smoker × ≥60 years × pre-pandemic | 0.84 (0.49-1.44) | -0.18 (0.28) | 0.532 |
|  | current smoker × ≥60 years × pandemic | 0.91 (0.40-2.08) | -0.10 (0.42) | 0.824 |
|  | current smoker × 40-59 years × pre-pandemic | 1.14 (0.78-1.66) | 0.13 (0.19) | 0.503 |
|  | current smoker × 40-59 years × pandemic | 0.59 (0.27-1.31) | -0.53 (0.41) | 0.212 |
|  | current smoker × 20-39 years × pandemic | 1.58 (0.96-2.57) | 0.45 (0.25) | 0.087 |
|  | depression symptoms × ≥60 years × pre-pandemic | 0.48 (0.27-0.86) | -0.74 (0.30) | 0.022 |
|  | depression symptoms × ≥60 years × pandemic | 1.76 (0.86-3.60) | 0.56 (0.37) | 0.136 |
|  | depression symptoms × 40-59 years × pre-pandemic | 0.97 (0.69-1.35) | -0.04 (0.17) | 0.841 |
|  | depression symptoms × 40-59 years × pandemic | 1.17 (0.63-2.19) | 0.16 (0.32) | 0.628 |
|  | depression symptoms × 20-39 years × pandemic | 0.76 (0.44-1.31) | -0.27 (0.28) | 0.332 |
|  | DII × ≥60 years × pre-pandemic | 0.70 (0.55-0.90) | -0.35 (0.12) | 0.009 |
|  | DII × ≥60 years × pandemic | 1.34 (0.97-1.86) | 0.30 (0.16) | 0.086 |
|  | DII × 40-59 years × pre-pandemic | 0.72 (0.57-0.89) | -0.33 (0.11) | 0.007 |
|  | DII × 40-59 years × pandemic | 1.34 (0.99-1.82) | 0.29 (0.16) | 0.072 |
|  | DII × 20-39 years × pandemic | 0.86 (0.66-1.12) | -0.15 (0.13) | 0.268 |
|  | divorced, separated or widowed × ≥60 years × pre-pandemic | 0.20 (0.07-0.54) | -1.62 (0.51) | 0.006 |
|  | divorced, separated or widowed × ≥60 years × pandemic | 2.46 (0.70-8.64) | 0.90 (0.64) | 0.179 |
|  | divorced, separated or widowed × 40-59 years × pre-pandemic | 0.88 (0.47-1.65) | -0.13 (0.32) | 0.684 |
|  | divorced, separated or widowed × 40-59 years × pandemic | 0.45 (0.17-1.22) | -0.80 (0.51) | 0.136 |
|  | divorced, separated or widowed × 20-39 years × pandemic | 1.03 (0.46-2.32) | 0.03 (0.41) | 0.939 |
|  | ex-smoker × ≥60 years × pre-pandemic | 0.39 (0.20-0.75) | -0.94 (0.34) | 0.012 |
|  | ex-smoker × ≥60 years × pandemic | 2.65 (1.02-6.90) | 0.98 (0.49) | 0.062 |
|  | ex-smoker × 40-59 years × pre-pandemic | 0.74 (0.31-1.74) | -0.31 (0.44) | 0.495 |
|  | ex-smoker × 40-59 years × pandemic | 1.26 (0.36-4.46) | 0.23 (0.65) | 0.726 |
|  | ex-smoker × 20-39 years × pandemic | 0.56 (0.23-1.41) | -0.57 (0.47) | 0.237 |
|  | full-time employee × ≥60 years × pre-pandemic | 0.69 (0.36-1.34) | -0.37 (0.34) | 0.29 |
|  | full-time employee × ≥60 years × pandemic | 2.26 (1.02-5.02) | 0.82 (0.41) | 0.06 |
|  | full-time employee × 40-59 years × pre-pandemic | 0.74 (0.38-1.44) | -0.30 (0.34) | 0.394 |
|  | full-time employee × 40-59 years × pandemic | 1.95 (0.75-5.07) | 0.67 (0.49) | 0.191 |
|  | full-time employee × 20-39 years × pandemic | 0.50 (0.26-0.95) | -0.70 (0.33) | 0.05 |
|  | health insurance × ≥60 years × pre-pandemic | 1.19 (0.52-2.72) | 0.17 (0.42) | 0.682 |
|  | health insurance × ≥60 years × pandemic | 0.86 (0.29-2.55) | -0.15 (0.55) | 0.788 |
|  | health insurance × 40-59 years × pre-pandemic | 0.54 (0.32-0.92) | -0.61 (0.27) | 0.034 |
|  | health insurance × 40-59 years × pandemic | 1.57 (0.62-3.97) | 0.45 (0.47) | 0.348 |
|  | health insurance × 20-39 years × pandemic | 0.94 (0.52-1.70) | -0.06 (0.30) | 0.839 |
|  | high active × ≥60 years × pre-pandemic | 1.93 (1.38-2.70) | 0.66 (0.17) | 0.001 |
|  | high active × ≥60 years × pandemic | 0.63 (0.38-1.03) | -0.46 (0.25) | 0.082 |
|  | high active × 40-59 years × pre-pandemic | 1.59 (0.99-2.55) | 0.46 (0.24) | 0.071 |
|  | high active × 40-59 years × pandemic | 0.55 (0.27-1.11) | -0.60 (0.36) | 0.112 |
|  | high active × 20-39 years × pandemic | 1.54 (0.91-2.63) | 0.43 (0.27) | 0.129 |
|  | high income × ≥60 years × pre-pandemic | 0.87 (0.35-2.18) | -0.13 (0.47) | 0.777 |
|  | high income × ≥60 years × pandemic | 2.08 (0.60-7.25) | 0.73 (0.64) | 0.266 |
|  | high income × 40-59 years × pre-pandemic | 0.68 (0.35-1.33) | -0.39 (0.34) | 0.277 |
|  | high income × 40-59 years × pandemic | 1.72 (0.53-5.62) | 0.54 (0.60) | 0.381 |
|  | high income × 20-39 years × pandemic | 0.75 (0.33-1.69) | -0.29 (0.41) | 0.496 |
|  | low active × ≥60 years × pandemic | 1.72 (1.09-2.70) | 0.54 (0.23) | 0.031 |
|  | low active × 40-59 years × pandemic | 1.89 (1.29-2.78) | 0.64 (0.20) | 0.004 |
|  | low income × ≥60 years × pandemic | 0.77 (0.31-1.90) | -0.26 (0.46) | 0.582 |
|  | low income × 40-59 years × pandemic | 1.05 (0.42-2.65) | 0.05 (0.47) | 0.92 |
|  | married or living with partner × ≥60 years × pre-pandemic | 0.25 (0.12-0.52) | -1.40 (0.38) | 0.002 |
|  | married or living with partner × ≥60 years × pandemic | 3.96 (1.54-10.17) | 1.38 (0.48) | 0.011 |
|  | married or living with partner × 40-59 years × pre-pandemic | 0.64 (0.39-1.06) | -0.44 (0.25) | 0.1 |
|  | married or living with partner × 40-59 years × pandemic | 1.03 (0.47-2.25) | 0.03 (0.40) | 0.946 |
|  | married or living with partner × 20-39 years × pandemic | 0.68 (0.40-1.18) | -0.38 (0.28) | 0.187 |
|  | middle income × ≥60 years × pre-pandemic | 0.83 (0.36-1.92) | -0.18 (0.43) | 0.676 |
|  | middle income × ≥60 years × pandemic | 2.73 (0.96-7.79) | 1.01 (0.53) | 0.077 |
|  | middle income × 40-59 years × pre-pandemic | 1.05 (0.57-1.94) | 0.05 (0.31) | 0.88 |
|  | middle income × 40-59 years × pandemic | 1.11 (0.46-2.70) | 0.10 (0.45) | 0.821 |
|  | middle income × 20-39 years × pandemic | 0.58 (0.32-1.05) | -0.54 (0.30) | 0.091 |
|  | moderate active × ≥60 years × pre-pandemic | 1.58 (0.71-3.52) | 0.45 (0.41) | 0.282 |
|  | moderate active × ≥60 years × pandemic | 1.07 (0.36-3.16) | 0.07 (0.55) | 0.905 |
|  | moderate active × 40-59 years × pre-pandemic | 2.56 (1.42-4.61) | 0.94 (0.30) | 0.006 |
|  | moderate active × 40-59 years × pandemic | 0.50 (0.19-1.34) | -0.69 (0.50) | 0.187 |
|  | moderate active × 20-39 years × pandemic | 1.32 (0.52-3.36) | 0.28 (0.48) | 0.562 |
|  | never-smoker × ≥60 years × pandemic | 1.24 (0.84-1.84) | 0.22 (0.20) | 0.3 |
|  | never-smoker × 40-59 years × pandemic | 1.49 (1.01-2.20) | 0.40 (0.20) | 0.06 |
|  | not-employed × ≥60 years × pandemic | 0.95 (0.56-1.60) | -0.06 (0.27) | 0.837 |
|  | not-employed × 40-59 years × pandemic | 0.97 (0.46-2.05) | -0.03 (0.38) | 0.941 |
|  | part-time employee × ≥60 years × pre-pandemic | 1.34 (0.66-2.71) | 0.29 (0.36) | 0.431 |
|  | part-time employee × ≥60 years × pandemic | 1.59 (0.61-4.15) | 0.46 (0.49) | 0.355 |
|  | part-time employee × 40-59 years × pre-pandemic | 1.16 (0.58-2.33) | 0.15 (0.36) | 0.683 |
|  | part-time employee × 40-59 years × pandemic | 0.85 (0.25-2.91) | -0.17 (0.63) | 0.794 |
|  | part-time employee × 20-39 years × pandemic | 0.81 (0.33-2.03) | -0.21 (0.47) | 0.664 |
|  | sleep hours (weekdays) <6h × ≥60 years × pandemic | 1.81 (1.00-3.30) | 0.60 (0.31) | 0.068 |
|  | sleep hours (weekdays) <6h × 40-59 years × pandemic | 1.15 (0.65-2.05) | 0.14 (0.29) | 0.63 |
|  | sleep hours (weekdays): 6-8h × ≥60 years × pre-pandemic | 1.08 (0.43-2.69) | 0.08 (0.47) | 0.872 |
|  | sleep hours (weekdays): 6-8h × ≥60 years × pandemic | 0.65 (0.22-1.94) | -0.42 (0.56) | 0.456 |
|  | sleep hours (weekdays): 6-8h × 40-59 years × pre-pandemic | 1.40 (0.64-3.06) | 0.34 (0.40) | 0.409 |
|  | sleep hours (weekdays): 6-8h × 40-59 years × pandemic | 0.70 (0.22-2.16) | -0.36 (0.58) | 0.538 |
|  | sleep hours (weekdays): 6-8h × 20-39 years × pandemic | 1.82 (0.80-4.11) | 0.60 (0.42) | 0.17 |
|  | sleep hours (weekdays)≥8h × ≥60 years × pre-pandemic | 1.13 (0.48-2.64) | 0.12 (0.43) | 0.785 |
|  | sleep hours (weekdays)≥8h × ≥60 years × pandemic | 0.50 (0.16-1.58) | -0.69 (0.59) | 0.255 |
|  | sleep hours (weekdays)≥8h × 40-59 years × pre-pandemic | 1.33 (0.60-2.96) | 0.28 (0.41) | 0.497 |
|  | sleep hours (weekdays)≥8h × 40-59 years × pandemic | 0.63 (0.17-2.32) | -0.46 (0.67) | 0.496 |
|  | sleep hours (weekdays)≥8h × 20-39 years × pandemic | 2.50 (0.93-6.71) | 0.92 (0.50) | 0.087 |
|  | sleep hours (weekends): 6-8h × ≥60 years × pre-pandemic | 0.89 (0.40-2.00) | -0.12 (0.41) | 0.784 |
|  | sleep hours (weekends): 6-8h × ≥60 years × pandemic | 1.21 (0.44-3.30) | 0.19 (0.51) | 0.717 |
|  | sleep hours (weekends): 6-8h × 40-59 years × pre-pandemic | 0.69 (0.36-1.30) | -0.38 (0.33) | 0.263 |
|  | sleep hours (weekends): 6-8h × 40-59 years × pandemic | 1.06 (0.42-2.69) | 0.06 (0.48) | 0.907 |
|  | sleep hours (weekends): 6-8h × 20-39 years × pandemic | 0.72 (0.31-1.68) | -0.32 (0.43) | 0.464 |
|  | sleep hours (weekends)≥8h × ≥60 years × pre-pandemic | 1.24 (0.65-2.37) | 0.22 (0.33) | 0.514 |
|  | sleep hours (weekends)≥8h × ≥60 years × pandemic | 0.73 (0.32-1.65) | -0.32 (0.42) | 0.458 |
|  | sleep hours (weekends)≥8h × 40-59 years × pre-pandemic | 0.58 (0.29-1.13) | -0.55 (0.34) | 0.126 |
|  | sleep hours (weekends)≥8h × 40-59 years × pandemic | 1.53 (0.60-3.94) | 0.43 (0.48) | 0.386 |
|  | sleep hours (weekends)≥8h × 20-39 years × pandemic | 1.02 (0.55-1.91) | 0.02 (0.32) | 0.942 |
|  | some college × ≥60 years × pre-pandemic | 0.78 (0.45-1.35) | -0.25 (0.28) | 0.387 |
|  | some college × ≥60 years × pandemic | 1.05 (0.44-2.50) | 0.04 (0.45) | 0.921 |
|  | some college × 40-59 years × pre-pandemic | 0.92 (0.57-1.49) | -0.09 (0.25) | 0.729 |
|  | some college × 40-59 years × pandemic | 0.79 (0.29-2.14) | -0.23 (0.51) | 0.65 |
|  | some college × 20-39 years × pandemic | 1.08 (0.48-2.40) | 0.07 (0.41) | 0.861 |
|  | alcohol consumption × depression symptoms × pre-pandemic | 1.04 (0.87-1.25) | 0.04 (0.09) | 0.648 |
|  | alcohol consumption × depression symptoms × pandemic | 0.75 (0.57-0.99) | -0.28 (0.14) | 0.048 |
|  | alcohol consumption × no/minimal depression × pandemic | 1.25 (1.04-1.50) | 0.22 (0.09) | 0.025 |
|  | CDAI × depression symptoms × pre-pandemic | 1.04 (0.86-1.25) | 0.04 (0.10) | 0.689 |
|  | CDAI × depression symptoms × pandemic | 1.02 (0.77-1.33) | 0.02 (0.14) | 0.913 |
|  | CDAI × no/minimal depression × pandemic | 0.96 (0.83-1.12) | -0.04 (0.08) | 0.634 |
|  | CDAI × alcohol consumption × pre-pandemic | 1.04 (0.95-1.15) | 0.04 (0.05) | 0.378 |
|  | CDAI × alcohol consumption × pandemic | 0.94 (0.80-1.11) | -0.06 (0.08) | 0.496 |
|  | CDAI × current smoker × pre-pandemic | 1.03 (0.78-1.36) | 0.03 (0.14) | 0.82 |
|  | CDAI × current smoker × pandemic | 1.09 (0.76-1.57) | 0.09 (0.19) | 0.645 |
|  | CDAI × ex-smoker × pre-pandemic | 1.18 (0.97-1.45) | 0.17 (0.10) | 0.117 |
|  | CDAI × ex-smoker × pandemic | 1.00 (0.76-1.33) | 0.00 (0.14) | 0.973 |
|  | CDAI × high active × pre-pandemic | 0.82 (0.70-0.96) | -0.20 (0.08) | 0.021 |
|  | CDAI × high active × pandemic | 1.35 (1.09-1.67) | 0.30 (0.11) | 0.013 |
|  | CDAI × moderate active × pre-pandemic | 0.99 (0.75-1.29) | -0.01 (0.14) | 0.924 |
|  | CDAI × moderate active × pandemic | 1.07 (0.71-1.61) | 0.06 (0.21) | 0.764 |
|  | current smoker × depression symptoms × pre-pandemic | 1.11 (0.65-1.90) | 0.10 (0.27) | 0.707 |
|  | current smoker × depression symptoms × pandemic | 0.36 (0.16-0.83) | -1.01 (0.42) | 0.025 |
|  | current smoker × no/minimal depression × pandemic | 2.02 (1.32-3.10) | 0.71 (0.22) | 0.004 |
|  | DII × depression symptoms × pre-pandemic | 0.94 (0.76-1.17) | -0.06 (0.11) | 0.595 |
|  | DII × depression symptoms × pandemic | 0.95 (0.72-1.24) | -0.06 (0.14) | 0.694 |
|  | DII × no/minimal depression × pandemic | 1.09 (0.93-1.27) | 0.08 (0.08) | 0.303 |
|  | DII × alcohol consumption × pandemic | 1.05 (0.91-1.23) | 0.05 (0.08) | 0.508 |
|  | DII × alcohol consumption × pre-pandemic | 0.92 (0.83-1.03) | -0.08 (0.05) | 0.147 |
|  | DII × current smoker × pandemic | 0.81 (0.56-1.17) | -0.22 (0.19) | 0.265 |
|  | DII × current smoker × pre-pandemic | 1.02 (0.80-1.29) | 0.02 (0.12) | 0.898 |
|  | DII × ex-smoker × pandemic | 0.97 (0.73-1.29) | -0.03 (0.14) | 0.826 |
|  | DII × ex-smoker × pre-pandemic | 0.87 (0.70-1.07) | -0.14 (0.11) | 0.207 |
|  | DII × high active × pandemic | 0.87 (0.68-1.10) | -0.14 (0.12) | 0.242 |
|  | DII × high active × pre-pandemic | 1.13 (0.92-1.39) | 0.12 (0.11) | 0.261 |
|  | DII × moderate active × pandemic | 1.08 (0.74-1.58) | 0.08 (0.19) | 0.683 |
|  | DII × moderate active × pre-pandemic | 0.99 (0.77-1.28) | -0.01 (0.13) | 0.955 |
|  | energy intake kcal × depression symptoms × pre-pandemic | 0.93 (0.78-1.11) | -0.07 (0.09) | 0.42 |
|  | energy intake kcal × depression symptoms × pandemic | 1.12 (0.87-1.44) | 0.11 (0.13) | 0.396 |
|  | energy intake kcal × no/minimal depression × pandemic | 0.91 (0.78-1.05) | -0.10 (0.07) | 0.197 |
|  | ex-smoker × depression symptoms × pre-pandemic | 0.89 (0.60-1.33) | -0.11 (0.20) | 0.584 |
|  | ex-smoker × depression symptoms × pandemic | 0.91 (0.54-1.54) | -0.09 (0.27) | 0.729 |
|  | ex-smoker × no/minimal depression × pandemic | 1.02 (0.73-1.43) | 0.02 (0.17) | 0.911 |
|  | high active × depression symptoms × pre-pandemic | 0.97 (0.63-1.48) | -0.03 (0.22) | 0.879 |
|  | high active × depression symptoms × pandemic | 0.74 (0.36-1.51) | -0.31 (0.37) | 0.412 |
|  | high active × no/minimal depression × pandemic | 1.16 (0.79-1.71) | 0.15 (0.20) | 0.456 |
|  | low active × depression symptoms × pandemic | 0.89 (0.65-1.23) | -0.11 (0.17) | 0.501 |
|  | moderate active × depression symptoms × pre-pandemic | 0.73 (0.39-1.36) | -0.32 (0.32) | 0.329 |
|  | moderate active × depression symptoms × pandemic | 1.45 (0.67-3.13) | 0.37 (0.39) | 0.358 |
|  | moderate active × no/minimal depression × pandemic | 0.93 (0.61-1.41) | -0.07 (0.21) | 0.739 |
|  | never-smoker × depression symptoms × pandemic | 1.00 (0.70-1.42) | -0.00 (0.18) | 0.998 |
|  | sleep hours (weekdays) <6h × depression symptoms × pandemic | 0.72 (0.39-1.33) | -0.33 (0.31) | 0.304 |
|  | sleep hours (weekends): 6-8h × depression symptoms × pre-pandemic | 0.85 (0.49-1.48) | -0.17 (0.28) | 0.565 |
|  | sleep hours (weekends): 6-8h × depression symptoms × pandemic | 1.58 (0.75-3.32) | 0.46 (0.38) | 0.243 |
|  | sleep hours (weekends): 6-8h × no/minimal depression × pandemic | 0.72 (0.44-1.16) | -0.33 (0.25) | 0.187 |
|  | sleep hours (weekends)≥8h × depression symptoms × pre-pandemic | 0.75 (0.47-1.21) | -0.28 (0.24) | 0.252 |
|  | sleep hours (weekends)≥8h × depression symptoms × pandemic | 1.13 (0.58-2.21) | 0.13 (0.34) | 0.716 |
|  | sleep hours (weekends)≥8h × no/minimal depression × pandemic | 1.00 (0.66-1.50) | -0.00 (0.21) | 0.993 |
|  | college graduate or above × divorced, separated or widowed × pre-pandemic | 0.65 (0.35-1.19) | -0.44 (0.31) | 0.181 |
|  | college graduate or above × divorced, separated or widowed × pandemic | 2.04 (0.84-4.94) | 0.71 (0.45) | 0.134 |
|  | college graduate or above × married or living with partner × pre-pandemic | 1.05 (0.58-1.90) | 0.05 (0.30) | 0.863 |
|  | college graduate or above × married or living with partner × pandemic | 1.67 (0.77-3.64) | 0.52 (0.40) | 0.211 |
|  | college graduate or above × no/minimal depression × pandemic | 0.47 (0.21-1.07) | -0.75 (0.41) | 0.089 |
|  | less than college × divorced, separated or widowed × pandemic | 0.64 (0.30-1.39) | -0.44 (0.39) | 0.276 |
|  | less than college × married or living with partner × pandemic | 0.90 (0.41-2.00) | -0.11 (0.41) | 0.799 |
|  | some college × divorced, separated or widowed × pre-pandemic | 0.81 (0.43-1.54) | -0.20 (0.32) | 0.536 |
|  | some college × divorced, separated or widowed × pandemic | 1.15 (0.46-2.87) | 0.14 (0.47) | 0.763 |
|  | some college × married or living with partner × pre-pandemic | 1.46 (0.72-2.94) | 0.38 (0.36) | 0.308 |
|  | some college × married or living with partner × pandemic | 0.63 (0.24-1.67) | -0.46 (0.50) | 0.367 |
|  | some college × no/minimal depression × pandemic | 1.22 (0.53-2.84) | 0.20 (0.43) | 0.646 |
|  | alcohol consumption × men × pre-pandemic | 1.03 (0.83-1.29) | 0.03 (0.11) | 0.766 |
|  | alcohol consumption × men × pandemic | 0.99 (0.69-1.42) | -0.01 (0.18) | 0.974 |
|  | alcohol consumption × women × pandemic | 1.14 (0.86-1.51) | 0.13 (0.15) | 0.379 |
|  | born outside the US × men × pre-pandemic | 0.72 (0.51-1.01) | -0.34 (0.17) | 0.065 |
|  | born outside the US × men × pandemic | 1.81 (1.00-3.27) | 0.59 (0.30) | 0.06 |
|  | born outside the US × women × pandemic | 0.73 (0.52-1.04) | -0.31 (0.18) | 0.092 |
|  | CDAI × men × pre-pandemic | 1.02 (0.83-1.25) | 0.02 (0.11) | 0.864 |
|  | CDAI × men × pandemic | 0.83 (0.59-1.16) | -0.19 (0.17) | 0.291 |
|  | CDAI × women × pandemic | 1.07 (0.84-1.35) | 0.07 (0.12) | 0.595 |
|  | college graduate or above × men × pre-pandemic | 1.56 (1.11-2.18) | 0.44 (0.17) | 0.017 |
|  | college graduate or above × men × pandemic | 0.62 (0.37-1.06) | -0.47 (0.27) | 0.095 |
|  | college graduate or above × women × pandemic | 0.97 (0.68-1.36) | -0.04 (0.18) | 0.844 |
|  | current smoker × men × pre-pandemic | 0.90 (0.62-1.30) | -0.11 (0.19) | 0.578 |
|  | current smoker × men × pandemic | 1.91 (0.89-4.11) | 0.65 (0.39) | 0.111 |
|  | current smoker × women × pandemic | 0.95 (0.54-1.67) | -0.05 (0.29) | 0.858 |
|  | depression symptoms × men × pre-pandemic | 0.77 (0.52-1.14) | -0.26 (0.20) | 0.2 |
|  | depression symptoms × men × pandemic | 1.40 (0.81-2.43) | 0.34 (0.28) | 0.244 |
|  | depression symptoms × women × pandemic | 0.71 (0.49-1.05) | -0.34 (0.20) | 0.097 |
|  | DII × men × pre-pandemic | 0.92 (0.72-1.18) | -0.08 (0.13) | 0.512 |
|  | DII × men × pandemic | 1.16 (0.84-1.60) | 0.15 (0.17) | 0.385 |
|  | DII × women × pandemic | 0.97 (0.77-1.23) | -0.03 (0.12) | 0.826 |
|  | divorced, separated or widowed × men × pre-pandemic | 0.65 (0.31-1.34) | -0.44 (0.37) | 0.251 |
|  | divorced, separated or widowed × men × pandemic | 1.22 (0.46-3.21) | 0.20 (0.49) | 0.69 |
|  | divorced, separated or widowed × women × pandemic | 0.75 (0.41-1.37) | -0.29 (0.31) | 0.362 |
|  | ex-smoker × men × pre-pandemic | 1.10 (0.69-1.74) | 0.09 (0.24) | 0.699 |
|  | ex-smoker × men × pandemic | 0.86 (0.49-1.51) | -0.15 (0.29) | 0.606 |
|  | ex-smoker × women × pandemic | 1.11 (0.77-1.59) | 0.10 (0.18) | 0.579 |
|  | full-time employee × men × pre-pandemic | 1.23 (0.78-1.92) | 0.20 (0.23) | 0.383 |
|  | full-time employee × men × pandemic | 0.93 (0.52-1.66) | -0.07 (0.30) | 0.803 |
|  | full-time employee × women × pandemic | 0.87 (0.57-1.32) | -0.14 (0.21) | 0.524 |
|  | health insurance × men × pre-pandemic | 1.47 (0.96-2.25) | 0.38 (0.22) | 0.088 |
|  | health insurance × men × pandemic | 1.03 (0.42-2.50) | 0.03 (0.45) | 0.95 |
|  | health insurance × women × pandemic | 1.05 (0.59-1.86) | 0.05 (0.29) | 0.874 |
|  | high active × men × pre-pandemic | 1.49 (1.00-2.23) | 0.40 (0.20) | 0.062 |
|  | high active × men × pandemic | 0.60 (0.35-1.04) | -0.50 (0.28) | 0.083 |
|  | high active × women × pandemic | 1.50 (0.98-2.29) | 0.40 (0.22) | 0.075 |
|  | high income × men × pre-pandemic | 1.19 (0.82-1.72) | 0.18 (0.19) | 0.359 |
|  | high income × men × pandemic | 1.10 (0.64-1.89) | 0.10 (0.27) | 0.73 |
|  | high income × women × pandemic | 1.14 (0.76-1.73) | 0.14 (0.21) | 0.528 |
|  | hypertension × men × pre-pandemic | 0.82 (0.62-1.09) | -0.20 (0.15) | 0.187 |
|  | hypertension × men × pandemic | 1.02 (0.67-1.56) | 0.02 (0.22) | 0.934 |
|  | hypertension × women × pandemic | 1.06 (0.77-1.47) | 0.06 (0.17) | 0.723 |
|  | low active × men × pandemic | 0.96 (0.67-1.39) | -0.04 (0.19) | 0.848 |
|  | low income × men × pandemic | 0.74 (0.45-1.22) | -0.30 (0.25) | 0.249 |
|  | men × less than college × pandemic | 1.21 (0.74-1.98) | 0.19 (0.25) | 0.447 |
|  | men × never married × pandemic | 0.65 (0.31-1.35) | -0.43 (0.37) | 0.257 |
|  | men × never-smoker × pandemic | 0.83 (0.62-1.11) | -0.19 (0.15) | 0.219 |
|  | men × no health insurance × pandemic | 0.84 (0.36-1.96) | -0.18 (0.43) | 0.685 |
|  | men × not-employed × pandemic | 0.93 (0.62-1.41) | -0.07 (0.21) | 0.743 |
|  | men × sleep hours (weekdays) <6h × pandemic | 1.18 (0.61-2.28) | 0.17 (0.34) | 0.627 |
|  | married or living with partner × men × pre-pandemic | 0.74 (0.40-1.37) | -0.31 (0.32) | 0.346 |
|  | married or living with partner × men × pandemic | 1.46 (0.61-3.50) | 0.38 (0.45) | 0.406 |
|  | married or living with partner × women × pandemic | 0.76 (0.43-1.34) | -0.28 (0.29) | 0.347 |
|  | middle income × men × pre-pandemic | 0.92 (0.52-1.61) | -0.09 (0.29) | 0.762 |
|  | middle income × men × pandemic | 1.34 (0.62-2.88) | 0.29 (0.39) | 0.459 |
|  | middle income × women × pandemic | 0.78 (0.53-1.15) | -0.25 (0.20) | 0.218 |
|  | moderate active × men × pre-pandemic | 1.01 (0.60-1.69) | 0.01 (0.26) | 0.977 |
|  | moderate active × men × pandemic | 1.20 (0.57-2.53) | 0.18 (0.38) | 0.636 |
|  | moderate active × women × pandemic | 0.92 (0.55-1.55) | -0.08 (0.26) | 0.764 |
|  | non-hypertension × men × pandemic | 0.87 (0.64-1.18) | -0.14 (0.16) | 0.381 |
|  | non-obese × men × pandemic | 0.88 (0.65-1.19) | -0.13 (0.15) | 0.403 |
|  | obesity × men × pre-pandemic | 0.85 (0.56-1.28) | -0.17 (0.21) | 0.437 |
|  | obesity × men × pandemic | 0.99 (0.54-1.81) | -0.01 (0.31) | 0.979 |
|  | obesity × women × pandemic | 1.02 (0.69-1.50) | 0.02 (0.20) | 0.93 |
|  | part-time employee × men × pre-pandemic | 1.35 (0.73-2.49) | 0.30 (0.31) | 0.354 |
|  | part-time employee × men × pandemic | 1.16 (0.43-3.10) | 0.15 (0.50) | 0.773 |
|  | part-time employee × women × pandemic | 0.97 (0.51-1.83) | -0.03 (0.32) | 0.925 |
|  | sleep hours (weekdays) <6h × men × pandemic | 0.63 (0.29-1.35) | -0.46 (0.39) | 0.246 |
|  | sleep hours (weekdays): 6-8h × men × pre-pandemic | 0.50 (0.30-0.85) | -0.69 (0.27) | 0.018 |
|  | sleep hours (weekdays): 6-8h × men × pandemic | 2.03 (0.82-5.04) | 0.71 (0.46) | 0.141 |
|  | sleep hours (weekdays): 6-8h × women × pandemic | 0.84 (0.43-1.65) | -0.17 (0.34) | 0.626 |
|  | sleep hours (weekdays)≥8h × men × pre-pandemic | 0.75 (0.46-1.25) | -0.28 (0.26) | 0.281 |
|  | sleep hours (weekdays)≥8h × men × pandemic | 1.09 (0.45-2.60) | 0.08 (0.45) | 0.855 |
|  | sleep hours (weekdays)≥8h × women × pandemic | 1.36 (0.72-2.55) | 0.30 (0.32) | 0.355 |
|  | sleep hours (weekends): 6-8h × men × pre-pandemic | 1.21 (0.71-2.08) | 0.19 (0.27) | 0.487 |
|  | sleep hours (weekends): 6-8h × men × pandemic | 0.57 (0.23-1.43) | -0.57 (0.47) | 0.242 |
|  | sleep hours (weekends): 6-8h × women × pandemic | 1.13 (0.68-1.87) | 0.12 (0.26) | 0.651 |
|  | sleep hours (weekends)≥8h × men × pre-pandemic | 1.27 (0.86-1.87) | 0.24 (0.20) | 0.24 |
|  | sleep hours (weekends)≥8h × men × pandemic | 0.78 (0.38-1.62) | -0.25 (0.37) | 0.513 |
|  | sleep hours (weekends)≥8h × women × pandemic | 1.20 (0.76-1.88) | 0.18 (0.23) | 0.445 |
|  | some college × men × pre-pandemic | 1.40 (0.91-2.16) | 0.34 (0.22) | 0.136 |
|  | some college × men × pandemic | 0.63 (0.30-1.29) | -0.47 (0.37) | 0.219 |
|  | some college × women × pandemic | 1.19 (0.81-1.75) | 0.18 (0.20) | 0.379 |
|  | US-born × men × pandemic | 0.79 (0.62-1.02) | -0.23 (0.13) | 0.085 |
|  | alcohol consumption × pandemic | 1.11 (0.98-1.26) | 0.11 (0.06) | 0.101 |
|  | born outside the US × pandemic | 0.99 (0.74-1.32) | -0.01 (0.15) | 0.928 |
|  | CDAI × pandemic | 0.97 (0.84-1.12) | -0.03 (0.07) | 0.697 |
|  | college graduate or above × pandemic | 0.76 (0.54-1.08) | -0.27 (0.18) | 0.135 |
|  | current smoker × pandemic | 1.36 (0.97-1.89) | 0.30 (0.17) | 0.082 |
|  | depression symptoms × pandemic | 0.84 (0.61-1.18) | -0.17 (0.17) | 0.326 |
|  | DII × pandemic | 1.06 (0.91-1.23) | 0.06 (0.07) | 0.445 |
|  | divorced, separated or widowed × pandemic | 0.89 (0.59-1.33) | -0.12 (0.21) | 0.561 |
|  | ex-smoker × pandemic | 0.99 (0.71-1.38) | -0.01 (0.17) | 0.946 |
|  | full-time employee × pandemic | 0.82 (0.62-1.08) | -0.20 (0.14) | 0.166 |
|  | health insurance × pandemic | 1.07 (0.69-1.65) | 0.07 (0.22) | 0.766 |
|  | high active × pandemic | 1.10 (0.79-1.54) | 0.10 (0.17) | 0.578 |
|  | high income × pandemic | 1.17 (0.85-1.61) | 0.16 (0.16) | 0.342 |
|  | hypertension × pandemic | 1.07 (0.79-1.44) | 0.06 (0.15) | 0.682 |
|  | married or living with partner × pandemic | 0.93 (0.63-1.39) | -0.07 (0.20) | 0.736 |
|  | middle income × pandemic | 0.88 (0.65-1.20) | -0.13 (0.16) | 0.427 |
|  | moderate active × pandemic | 1.01 (0.69-1.47) | 0.01 (0.19) | 0.967 |
|  | obesity × pandemic | 1.02 (0.77-1.34) | 0.02 (0.14) | 0.911 |
|  | part-time employee × pandemic | 1.04 (0.69-1.55) | 0.03 (0.21) | 0.866 |
|  | sleep hours (weekdays): 6-8h × pandemic | 1.26 (0.82-1.93) | 0.23 (0.22) | 0.302 |
|  | sleep hours (weekdays)≥8h × pandemic | 1.50 (0.85-2.65) | 0.41 (0.29) | 0.173 |
|  | sleep hours (weekends): 6-8h × pandemic | 0.84 (0.57-1.24) | -0.17 (0.20) | 0.397 |
|  | sleep hours (weekends)≥8h × pandemic | 1.07 (0.75-1.54) | 0.07 (0.18) | 0.712 |
|  | some college × pandemic | 0.95 (0.70-1.28) | -0.05 (0.15) | 0.735 |
|  | age × full-time employee | 1.00 (0.98-1.02) | 0.00 (0.01) | 0.895 |
|  | age × full-time employee × pandemic | 1.02 (1.00-1.05) | 0.02 (0.01) | 0.131 |
|  | age × not-employed × pandemic | 1.00 (0.99-1.02) | 0.00 (0.01) | 0.841 |
|  | age × part-time employee | 1.03 (1.01-1.05) | 0.03 (0.01) | 0.018 |
|  | age × part-time employee × pandemic | 1.00 (0.97-1.04) | 0.00 (0.02) | 0.983 |
|  | full-time employee × pandemic | 0.33 (0.08-1.31) | -1.10 (0.70) | 0.144 |
|  | men × age × full-time employee | 1.00 (0.97-1.03) | 0.00 (0.01) | 0.931 |
|  | men × age × full-time employee × pandemic | 0.99 (0.95-1.03) | -0.01 (0.02) | 0.689 |
|  | men × age × not-employed × pandemic | 1.00 (0.98-1.03) | 0.00 (0.01) | 0.877 |
|  | men × age × part-time employee | 0.97 (0.94-0.99) | -0.03 (0.01) | 0.034 |
|  | men × age × part-time employee × pandemic | 1.00 (0.96-1.05) | 0.00 (0.02) | 0.851 |
|  | men × full-time employee | 1.18 (0.22-6.20) | 0.16 (0.85) | 0.851 |
|  | men × full-time employee × pandemic | 1.43 (0.12-16.92) | 0.36 (1.26) | 0.781 |
|  | men × not-employed × age | 1.00 (0.98-1.01) | -0.00 (0.01) | 0.711 |
|  | men × not-employed × pandemic | 0.83 (0.14-4.87) | -0.19 (0.91) | 0.837 |
|  | men × part-time employee | 6.45 (1.55-26.77) | 1.86 (0.73) | 0.026 |
|  | men × part-time employee × pandemic | 0.97 (0.06-15.25) | -0.03 (1.40) | 0.984 |
|  | part-time employee × pandemic | 0.85 (0.11-6.90) | -0.16 (1.07) | 0.884 |
|  | age × alcohol consumption | 1.00 (0.98-1.02) | -0.00 (0.01) | 0.998 |
|  | age × alcohol consumption × pandemic | 1.01 (0.99-1.03) | 0.01 (0.01) | 0.477 |
|  | alcohol consumption × pandemic | 0.69 (0.21-2.20) | -0.38 (0.59) | 0.535 |
|  | men × age × alcohol consumption | 1.00 (0.99-1.02) | 0.00 (0.01) | 0.608 |
|  | men × age × alcohol consumption × pandemic | 1.00 (0.97-1.02) | -0.00 (0.01) | 0.68 |
|  | men × alcohol consumption | 0.82 (0.34-1.96) | -0.20 (0.44) | 0.66 |
|  | men × alcohol consumption × pandemic | 1.39 (0.41-4.73) | 0.33 (0.62) | 0.599 |
|  | age × born outside the US | 0.99 (0.97-1.01) | -0.01 (0.01) | 0.269 |
|  | age × born outside the US × pandemic | 1.02 (0.99-1.05) | 0.02 (0.02) | 0.219 |
|  | age × US-born × pandemic | 1.00 (0.99-1.02) | 0.00 (0.01) | 0.55 |
|  | born outside the US × pandemic | 0.29 (0.06-1.50) | -1.23 (0.83) | 0.157 |
|  | men × age × born outside the US | 1.03 (1.01-1.05) | 0.03 (0.01) | 0.024 |
|  | men × age × born outside the US × pandemic | 0.97 (0.92-1.01) | -0.03 (0.02) | 0.175 |
|  | men × born outside the US | 0.18 (0.06-0.60) | -1.69 (0.60) | 0.011 |
|  | men × born outside the US × pandemic | 9.19 (0.65-129.39) | 2.22 (1.35) | 0.117 |
|  | US-born × men × age | 0.99 (0.97-1.00) | -0.01 (0.01) | 0.033 |
|  | US-born × men × age × pandemic | 1.01 (0.99-1.03) | 0.01 (0.01) | 0.363 |
|  | US-born × men × pandemic | 0.52 (0.18-1.51) | -0.66 (0.55) | 0.243 |
|  | age × CDAI | 1.01 (1.00-1.02) | 0.01 (0.00) | 0.153 |
|  | age × CDAI × pandemic | 0.99 (0.98-1.00) | -0.01 (0.01) | 0.097 |
|  | CDAI × pandemic | 1.88 (0.90-3.93) | 0.63 (0.38) | 0.109 |
|  | men × age × CDAI | 1.00 (0.99-1.01) | -0.00 (0.01) | 0.646 |
|  | men × age × CDAI × pandemic | 1.01 (0.99-1.03) | 0.01 (0.01) | 0.253 |
|  | men × CDAI | 1.17 (0.60-2.29) | 0.16 (0.34) | 0.653 |
|  | men × CDAI × pandemic | 0.45 (0.15-1.39) | -0.80 (0.57) | 0.181 |
|  | age × depression symptoms | 0.98 (0.97-1.00) | -0.02 (0.01) | 0.047 |
|  | age × depression symptoms × pandemic | 1.01 (0.99-1.03) | 0.01 (0.01) | 0.241 |
|  | depression symptoms × pandemic | 0.43 (0.13-1.41) | -0.85 (0.61) | 0.179 |
|  | men × age × depression symptoms | 1.01 (0.98-1.03) | 0.01 (0.01) | 0.653 |
|  | men × age × depression symptoms × pandemic | 1.01 (0.97-1.04) | 0.01 (0.02) | 0.671 |
|  | men × age × pandemic | 1.00 (0.99-1.02) | 0.00 (0.01) | 0.708 |
|  | men × depression symptoms | 0.56 (0.15-2.09) | -0.58 (0.68) | 0.397 |
|  | men × depression symptoms × pandemic | 0.98 (0.19-5.14) | -0.02 (0.85) | 0.981 |
|  | age × DII | 0.99 (0.98-1.00) | -0.01 (0.00) | 0.027 |
|  | age × DII × pandemic | 1.01 (1.00-1.03) | 0.01 (0.01) | 0.073 |
|  | DII × pandemic | 0.48 (0.21-1.07) | -0.74 (0.41) | 0.088 |
|  | men × age × DII | 1.00 (0.99-1.01) | 0.00 (0.01) | 0.691 |
|  | men × age × DII × pandemic | 0.99 (0.97-1.01) | -0.01 (0.01) | 0.382 |
|  | men × DII | 0.79 (0.40-1.59) | -0.23 (0.36) | 0.522 |
|  | men × DII × pandemic | 2.00 (0.58-6.87) | 0.69 (0.63) | 0.286 |
|  | age × college graduate or above | 1.01 (0.99-1.03) | 0.01 (0.01) | 0.286 |
|  | age × college graduate or above × pandemic | 1.00 (0.97-1.04) | 0.00 (0.02) | 0.895 |
|  | age × less than college × pandemic | 1.01 (0.99-1.03) | 0.01 (0.01) | 0.467 |
|  | age × some college | 1.00 (0.98-1.01) | -0.00 (0.01) | 0.773 |
|  | age × some college × pandemic | 0.99 (0.96-1.02) | -0.01 (0.02) | 0.631 |
|  | college graduate or above × pandemic | 0.87 (0.12-6.19) | -0.14 (1.00) | 0.888 |
|  | less than college × men × age | 1.00 (0.98-1.01) | -0.00 (0.01) | 0.454 |
|  | men × age × college graduate or above | 0.98 (0.96-1.01) | -0.02 (0.01) | 0.179 |
|  | men × age × college graduate or above × pandemic | 1.02 (0.98-1.06) | 0.02 (0.02) | 0.353 |
|  | men × age × less than college × pandemic | 0.99 (0.97-1.02) | -0.01 (0.01) | 0.68 |
|  | men × age × some college | 1.00 (0.98-1.03) | 0.00 (0.01) | 0.911 |
|  | men × age × some college × pandemic | 1.01 (0.97-1.05) | 0.01 (0.02) | 0.614 |
|  | men × college graduate or above | 3.65 (0.82-16.32) | 1.29 (0.76) | 0.119 |
|  | men × college graduate or above × pandemic | 0.25 (0.02-2.46) | -1.40 (1.17) | 0.258 |
|  | men × less than college × pandemic | 1.67 (0.28-10.12) | 0.51 (0.92) | 0.588 |
|  | men × some college | 1.25 (0.32-4.87) | 0.22 (0.70) | 0.756 |
|  | men × some college × pandemic | 0.35 (0.03-4.25) | -1.06 (1.28) | 0.425 |
|  | some college × pandemic | 1.89 (0.28-12.83) | 0.64 (0.98) | 0.529 |
|  | age × health insurance | 1.01 (0.99-1.04) | 0.01 (0.01) | 0.425 |
|  | age × health insurance × pandemic | 0.99 (0.96-1.03) | -0.01 (0.02) | 0.746 |
|  | age × no health insurance × pandemic | 1.01 (0.98-1.05) | 0.01 (0.02) | 0.512 |
|  | health insurance × pandemic | 1.36 (0.24-7.77) | 0.31 (0.89) | 0.733 |
|  | men × age × health insurance | 0.96 (0.92-1.00) | -0.04 (0.02) | 0.071 |
|  | men × age × health insurance × pandemic | 1.00 (0.93-1.08) | 0.00 (0.04) | 0.936 |
|  | men × age × no health insurance × pandemic | 1.00 (0.94-1.08) | 0.00 (0.04) | 0.915 |
|  | men × health insurance | 9.88 (1.74-56.03) | 2.29 (0.89) | 0.018 |
|  | men × health insurance × pandemic | 1.09 (0.04-32.54) | 0.08 (1.73) | 0.962 |
|  | men × no health insurance × age | 1.03 (0.99-1.07) | 0.03 (0.02) | 0.197 |
|  | men × no health insurance × pandemic | 0.56 (0.02-12.88) | -0.57 (1.60) | 0.724 |
|  | age × divorced, separated or widowed | 0.97 (0.95-1.00) | -0.03 (0.01) | 0.068 |
|  | age × divorced, separated or widowed × pandemic | 1.02 (0.97-1.06) | 0.02 (0.02) | 0.458 |
|  | age × married or living with partner | 0.98 (0.96-1.00) | -0.02 (0.01) | 0.056 |
|  | age × married or living with partner × pandemic | 1.03 (1.00-1.06) | 0.03 (0.02) | 0.063 |
|  | age × never married × pandemic | 0.99 (0.96-1.01) | -0.01 (0.01) | 0.318 |
|  | divorced, separated or widowed × pandemic | 0.39 (0.04-4.25) | -0.94 (1.22) | 0.458 |
|  | men × age × divorced, separated or widowed | 0.98 (0.95-1.02) | -0.02 (0.02) | 0.374 |
|  | men × age × divorced, separated or widowed × pandemic | 1.00 (0.95-1.06) | 0.00 (0.03) | 0.91 |
|  | men × age × married or living with partner | 1.00 (0.96-1.04) | -0.00 (0.02) | 0.839 |
|  | men × age × married or living with partner × pandemic | 0.99 (0.94-1.04) | -0.01 (0.02) | 0.614 |
|  | men × age × never married × pandemic | 1.01 (0.97-1.05) | 0.01 (0.02) | 0.702 |
|  | men × divorced, separated or widowed | 1.59 (0.27-9.27) | 0.46 (0.90) | 0.619 |
|  | men × divorced, separated or widowed × pandemic | 0.99 (0.05-18.85) | -0.01 (1.51) | 0.993 |
|  | men × married or living with partner | 0.82 (0.12-5.46) | -0.20 (0.97) | 0.842 |
|  | men × married or living with partner × pandemic | 2.88 (0.27-30.65) | 1.06 (1.21) | 0.4 |
|  | men × never married × age | 1.00 (0.97-1.03) | -0.00 (0.02) | 0.866 |
|  | men × never married × pandemic | 0.42 (0.06-2.88) | -0.87 (0.98) | 0.395 |
|  | married or living with partner × pandemic | 0.18 (0.04-0.80) | -1.70 (0.76) | 0.046 |
|  | age × high active | 1.01 (1.00-1.03) | 0.01 (0.01) | 0.187 |
|  | age × high active × pandemic | 0.99 (0.97-1.02) | -0.01 (0.01) | 0.494 |
|  | age × low active × pandemic | 1.01 (0.99-1.02) | 0.01 (0.01) | 0.27 |
|  | age × moderate active | 1.01 (0.99-1.04) | 0.01 (0.01) | 0.4 |
|  | age × moderate active × pandemic | 1.00 (0.97-1.03) | 0.00 (0.02) | 0.811 |
|  | high active × pandemic | 2.06 (0.50-8.49) | 0.72 (0.72) | 0.338 |
|  | low active × men × age | 0.99 (0.97-1.01) | -0.01 (0.01) | 0.219 |
|  | low active × men × age × pandemic | 1.00 (0.98-1.03) | 0.00 (0.01) | 0.802 |
|  | low active × men × pandemic | 0.81 (0.19-3.37) | -0.21 (0.73) | 0.775 |
|  | men × age × high active | 1.00 (0.97-1.04) | 0.00 (0.02) | 0.906 |
|  | men × age × high active × pandemic | 1.00 (0.96-1.05) | 0.00 (0.02) | 0.884 |
|  | men × age × moderate active | 1.00 (0.97-1.03) | 0.00 (0.01) | 0.963 |
|  | men × age × moderate active × pandemic | 0.99 (0.95-1.03) | -0.01 (0.02) | 0.551 |
|  | men × high active | 1.22 (0.19-7.95) | 0.20 (0.96) | 0.842 |
|  | men × high active × pandemic | 0.58 (0.04-9.10) | -0.55 (1.40) | 0.705 |
|  | men × moderate active | 0.92 (0.19-4.49) | -0.08 (0.81) | 0.92 |
|  | men × moderate active × pandemic | 2.25 (0.27-18.54) | 0.81 (1.08) | 0.467 |
|  | moderate active × pandemic | 0.75 (0.14-4.13) | -0.28 (0.87) | 0.75 |
|  | age × high income | 1.00 (0.97-1.03) | -0.00 (0.02) | 0.995 |
|  | age × high income × pandemic | 1.02 (0.98-1.06) | 0.02 (0.02) | 0.307 |
|  | age × low income × pandemic | 0.99 (0.96-1.01) | -0.01 (0.01) | 0.305 |
|  | age × middle income | 1.00 (0.98-1.02) | 0.00 (0.01) | 0.998 |
|  | age × middle income × pandemic | 1.03 (1.00-1.06) | 0.03 (0.01) | 0.081 |
|  | high income × pandemic | 0.43 (0.07-2.51) | -0.85 (0.90) | 0.366 |
|  | low income × men × age | 0.99 (0.98-1.01) | -0.01 (0.01) | 0.356 |
|  | low income × men × pandemic | 0.25 (0.06-1.11) | -1.38 (0.76) | 0.095 |
|  | men × age × high income | 1.00 (0.97-1.03) | 0.00 (0.02) | 0.927 |
|  | men × age × high income × pandemic | 0.98 (0.94-1.02) | -0.02 (0.02) | 0.279 |
|  | men × age × low income × pandemic | 1.02 (0.99-1.05) | 0.02 (0.01) | 0.152 |
|  | men × age × middle income | 0.99 (0.97-1.01) | -0.01 (0.01) | 0.453 |
|  | men × age × middle income × pandemic | 0.98 (0.95-1.01) | -0.02 (0.02) | 0.253 |
|  | men × high income | 1.20 (0.25-5.74) | 0.18 (0.80) | 0.822 |
|  | men × high income × pandemic | 3.28 (0.41-26.29) | 1.19 (1.06) | 0.288 |
|  | men × middle income | 1.51 (0.46-4.89) | 0.41 (0.60) | 0.51 |
|  | men × middle income × pandemic | 3.88 (0.62-24.18) | 1.36 (0.93) | 0.174 |
|  | middle income × pandemic | 0.19 (0.05-0.68) | -1.68 (0.66) | 0.028 |
|  | age × sleep hours (weekdays) <6h × pandemic | 1.02 (0.98-1.06) | 0.02 (0.02) | 0.303 |
|  | age × sleep hours (weekdays): 6-8h | 0.99 (0.96-1.02) | -0.01 (0.01) | 0.412 |
|  | age × sleep hours (weekdays): 6-8h × pandemic | 0.99 (0.96-1.03) | -0.01 (0.02) | 0.682 |
|  | age × sleep hours (weekdays)≥8h | 0.98 (0.96-1.01) | -0.02 (0.01) | 0.273 |
|  | age × sleep hours (weekdays)≥8h × pandemic | 0.98 (0.95-1.02) | -0.02 (0.02) | 0.32 |
|  | men × age × sleep hours (weekdays): 6-8h | 1.02 (0.99-1.06) | 0.02 (0.02) | 0.229 |
|  | men × age × sleep hours (weekdays): 6-8h × pandemic | 1.01 (0.95-1.07) | 0.01 (0.03) | 0.804 |
|  | men × age × sleep hours (weekdays)≥8h | 1.03 (0.99-1.08) | 0.03 (0.02) | 0.181 |
|  | men × age × sleep hours (weekdays)≥8h × pandemic | 1.01 (0.94-1.07) | 0.01 (0.03) | 0.876 |
|  | men × sleep hours (weekdays): 6-8h | 0.18 (0.03-1.13) | -1.72 (0.94) | 0.094 |
|  | men × sleep hours (weekdays): 6-8h × pandemic | 1.27 (0.05-32.39) | 0.24 (1.65) | 0.889 |
|  | men × sleep hours (weekdays)≥8h | 0.15 (0.01-1.66) | -1.93 (1.24) | 0.149 |
|  | men × sleep hours (weekdays)≥8h × pandemic | 0.71 (0.02-28.32) | -0.34 (1.88) | 0.858 |
|  | sleep hours (weekdays) <6h × men × age | 0.96 (0.93-1.00) | -0.04 (0.02) | 0.096 |
|  | sleep hours (weekdays) <6h × men × age × pandemic | 1.00 (0.94-1.06) | -0.00 (0.03) | 0.919 |
|  | sleep hours (weekdays) <6h × men × pandemic | 0.86 (0.03-27.27) | -0.15 (1.77) | 0.932 |
|  | sleep hours (weekdays): 6-8h × pandemic | 1.40 (0.18-10.82) | 0.33 (1.04) | 0.755 |
|  | sleep hours (weekdays)≥8h × pandemic | 4.02 (0.62-26.21) | 1.39 (0.96) | 0.174 |
|  | age × sleep hours (weekdays) <6h × pandemic | 1.01 (0.99-1.03) | 0.01 (0.01) | 0.235 |
|  | age × sleep hours (weekends): 6-8h | 1.00 (0.97-1.03) | -0.00 (0.02) | 0.905 |
|  | age × sleep hours (weekends): 6-8h × pandemic | 1.00 (0.96-1.03) | -0.00 (0.02) | 0.858 |
|  | age × sleep hours (weekends)≥8h | 1.00 (0.98-1.02) | -0.00 (0.01) | 0.919 |
|  | age × sleep hours (weekends)≥8h × pandemic | 0.99 (0.97-1.02) | -0.01 (0.01) | 0.526 |
|  | men × age × sleep hours (weekends): 6-8h | 0.99 (0.96-1.03) | -0.01 (0.02) | 0.631 |
|  | men × age × sleep hours (weekends): 6-8h × pandemic | 1.03 (0.98-1.08) | 0.03 (0.02) | 0.23 |
|  | men × age × sleep hours (weekends)≥8h | 1.01 (0.99-1.02) | 0.01 (0.01) | 0.229 |
|  | men × age × sleep hours (weekends)≥8h × pandemic | 1.01 (0.97-1.04) | 0.01 (0.02) | 0.773 |
|  | men × sleep hours (weekdays) <6h × pandemic | 1.71 (0.31-9.56) | 0.54 (0.88) | 0.554 |
|  | men × sleep hours (weekends): 6-8h | 2.12 (0.30-14.89) | 0.75 (0.99) | 0.464 |
|  | men × sleep hours (weekends): 6-8h × pandemic | 0.13 (0.01-1.90) | -2.08 (1.39) | 0.162 |
|  | men × sleep hours (weekends)≥8h | 0.85 (0.38-1.86) | -0.17 (0.40) | 0.686 |
|  | men × sleep hours (weekends)≥8h × pandemic | 0.57 (0.08-3.99) | -0.56 (0.99) | 0.584 |
|  | sleep hours (weekdays) <6h × men × age | 0.99 (0.97-1.00) | -0.01 (0.01) | 0.104 |
|  | sleep hours (weekdays) <6h × men × age × pandemic | 0.99 (0.96-1.02) | -0.01 (0.02) | 0.663 |
|  | sleep hours (weekends): 6-8h × pandemic | 1.25 (0.22-7.09) | 0.22 (0.89) | 0.808 |
|  | sleep hours (weekends)≥8h × pandemic | 1.90 (0.58-6.25) | 0.64 (0.61) | 0.313 |
|  | age × current smoker | 1.02 (0.99-1.05) | 0.02 (0.01) | 0.158 |
|  | age × current smoker × pandemic | 0.99 (0.95-1.04) | -0.01 (0.02) | 0.752 |
|  | age × ex-smoker | 0.99 (0.98-1.01) | -0.01 (0.01) | 0.228 |
|  | age × ex-smoker × pandemic | 1.01 (0.99-1.03) | 0.01 (0.01) | 0.446 |
|  | age × never-smoker × pandemic | 1.01 (0.99-1.02) | 0.01 (0.01) | 0.364 |
|  | current smoker × pandemic | 1.01 (0.07-15.46) | 0.01 (1.39) | 0.993 |
|  | ex-smoker × pandemic | 0.59 (0.15-2.31) | -0.53 (0.70) | 0.465 |
|  | men × age × current smoker | 0.97 (0.94-1.01) | -0.03 (0.02) | 0.147 |
|  | men × age × current smoker × pandemic | 1.01 (0.95-1.07) | 0.01 (0.03) | 0.793 |
|  | men × age × ex-smoker | 0.98 (0.96-1.01) | -0.02 (0.01) | 0.163 |
|  | men × age × ex-smoker × pandemic | 1.03 (0.99-1.06) | 0.03 (0.02) | 0.18 |
|  | men × age × never-smoker × pandemic | 1.00 (0.98-1.01) | -0.00 (0.01) | 0.618 |
|  | men × current smoker | 3.40 (0.56-20.77) | 1.22 (0.92) | 0.212 |
|  | men × current smoker × pandemic | 1.82 (0.06-51.83) | 0.60 (1.71) | 0.732 |
|  | men × ex-smoker | 2.45 (0.63-9.56) | 0.90 (0.69) | 0.222 |
|  | men × ex-smoker × pandemic | 0.24 (0.03-1.96) | -1.43 (1.07) | 0.209 |
|  | men × never-smoker × age | 1.00 (0.99-1.01) | -0.00 (0.01) | 0.961 |
|  | men × never-smoker × pandemic | 0.98 (0.39-2.44) | -0.02 (0.47) | 0.965 |
|  | men × ≥60 years | 0.74 (0.45-1.21) | -0.30 (0.25) | 0.243 |
|  | men × ≥60 years × pandemic | 1.15 (0.56-2.37) | 0.14 (0.37) | 0.706 |
|  | men × 40-59 years | 0.78 (0.48-1.28) | -0.24 (0.25) | 0.342 |
|  | men × 40-59 years × pandemic | 1.75 (0.87-3.53) | 0.56 (0.36) | 0.13 |
|  | men × pandemic | 0.69 (0.40-1.21) | -0.37 (0.28) | 0.21 |
|  | sex × ≥60 years × pandemic | 1.44 (0.84-2.48) | 0.36 (0.28) | 0.202 |
|  | sex × 40-59 years × pandemic | 1.07 (0.71-1.61) | 0.07 (0.21) | 0.74 |
|  | DII × pandemic | 0.94 (0.64-1.39) | -0.06 (0.20) | 0.763 |
|  | DII × sleep hours (weekdays): 6-8h | 0.98 (0.66-1.44) | -0.02 (0.20) | 0.905 |
|  | DII × sleep hours (weekdays): 6-8h × pandemic | 1.14 (0.69-1.86) | 0.13 (0.25) | 0.616 |
|  | DII × sleep hours (weekdays)≥8h | 1.04 (0.72-1.50) | 0.04 (0.19) | 0.842 |
|  | DII × sleep hours (weekdays)≥8h × pandemic | 1.13 (0.74-1.73) | 0.12 (0.22) | 0.575 |
|  | DII × pandemic | 0.88 (0.63-1.22) | -0.13 (0.17) | 0.453 |
|  | DII × sleep hours (weekends): 6-8h | 0.88 (0.63-1.22) | -0.13 (0.17) | 0.444 |
|  | DII × sleep hours (weekends): 6-8h × pandemic | 1.22 (0.82-1.82) | 0.20 (0.20) | 0.345 |
|  | DII × sleep hours (weekends)≥8h | 0.85 (0.63-1.15) | -0.16 (0.15) | 0.315 |
|  | DII × sleep hours (weekends)≥8h × pandemic | 1.27 (0.87-1.85) | 0.24 (0.19) | 0.225 |
|  | hypertension × full-time employee | 1.02 (0.73-1.42) | 0.02 (0.17) | 0.92 |
|  | hypertension × full-time employee × pandemic | 0.99 (0.62-1.56) | -0.01 (0.23) | 0.956 |
|  | hypertension × pandemic | 1.04 (0.73-1.50) | 0.04 (0.18) | 0.826 |
|  | hypertension × part-time employee | 1.25 (0.69-2.25) | 0.22 (0.30) | 0.467 |
|  | hypertension × part-time employee × pandemic | 1.06 (0.52-2.17) | 0.06 (0.36) | 0.873 |
|  | non-hypertension × full-time employee × pandemic | 0.83 (0.59-1.16) | -0.19 (0.17) | 0.29 |
|  | non-hypertension × part-time employee × pandemic | 1.03 (0.65-1.65) | 0.03 (0.24) | 0.894 |
|  | hypertension × alcohol consumption | 1.00 (0.86-1.17) | 0.00 (0.08) | 0.97 |
|  | hypertension × alcohol consumption × pandemic | 0.91 (0.72-1.15) | -0.09 (0.12) | 0.447 |
|  | hypertension × pandemic | 1.10 (0.79-1.51) | 0.09 (0.16) | 0.586 |
|  | non-hypertension × alcohol consumption × pandemic | 1.15 (1.00-1.33) | 0.14 (0.07) | 0.056 |
|  | hypertension × born outside the US | 0.97 (0.61-1.55) | -0.03 (0.24) | 0.904 |
|  | hypertension × born outside the US × pandemic | 1.01 (0.55-1.88) | 0.01 (0.32) | 0.966 |
|  | hypertension × pandemic | 1.06 (0.75-1.50) | 0.06 (0.18) | 0.74 |
|  | non-hypertension × born outside the US × pandemic | 0.99 (0.65-1.51) | -0.01 (0.22) | 0.951 |
|  | hypertension × CDAI | 1.02 (0.89-1.17) | 0.02 (0.07) | 0.779 |
|  | hypertension × CDAI × pandemic | 1.14 (0.93-1.38) | 0.13 (0.10) | 0.211 |
|  | hypertension × pandemic | 1.08 (0.80-1.47) | 0.08 (0.16) | 0.627 |
|  | non-hypertension × CDAI × pandemic | 0.94 (0.79-1.11) | -0.07 (0.09) | 0.455 |
|  | hypertension × depression symptoms | 1.22 (0.88-1.69) | 0.20 (0.17) | 0.241 |
|  | hypertension × depression symptoms × pandemic | 0.57 (0.35-0.93) | -0.56 (0.25) | 0.034 |
|  | hypertension × pandemic | 1.24 (0.90-1.71) | 0.21 (0.16) | 0.203 |
|  | non-hypertension × depression symptoms × pandemic | 1.07 (0.71-1.60) | 0.06 (0.21) | 0.759 |
|  | hypertension × DII | 0.98 (0.84-1.15) | -0.02 (0.08) | 0.813 |
|  | hypertension × DII × pandemic | 0.90 (0.71-1.14) | -0.10 (0.12) | 0.401 |
|  | hypertension × pandemic | 1.08 (0.80-1.47) | 0.08 (0.16) | 0.628 |
|  | non-hypertension × DII × pandemic | 1.10 (0.92-1.31) | 0.09 (0.09) | 0.314 |
|  | hypertension × college graduate or above | 1.20 (0.75-1.90) | 0.18 (0.24) | 0.456 |
|  | hypertension × college graduate or above × pandemic | 1.45 (0.76-2.78) | 0.37 (0.33) | 0.27 |
|  | hypertension × pandemic | 0.92 (0.56-1.52) | -0.08 (0.25) | 0.758 |
|  | hypertension × some college | 0.84 (0.55-1.27) | -0.18 (0.21) | 0.411 |
|  | hypertension × some college × pandemic | 0.87 (0.47-1.58) | -0.14 (0.31) | 0.642 |
|  | non-hypertension × college graduate or above × pandemic | 0.66 (0.40-1.09) | -0.41 (0.25) | 0.121 |
|  | non-hypertension × some college × pandemic | 1.00 (0.63-1.57) | -0.00 (0.23) | 0.988 |
|  | hypertension × health insurance | 0.72 (0.44-1.18) | -0.33 (0.25) | 0.205 |
|  | hypertension × health insurance × pandemic | 2.50 (1.30-4.81) | 0.92 (0.33) | 0.01 |
|  | hypertension × pandemic | 0.46 (0.24-0.86) | -0.78 (0.32) | 0.022 |
|  | non-hypertension × health insurance × pandemic | 0.79 (0.51-1.23) | -0.23 (0.22) | 0.303 |
|  | hypertension × divorced, separated or widowed | 0.50 (0.29-0.88) | -0.68 (0.28) | 0.024 |
|  | hypertension × divorced, separated or widowed × pandemic | 0.99 (0.53-1.87) | -0.01 (0.32) | 0.984 |
|  | hypertension × married or living with partner | 0.86 (0.53-1.39) | -0.15 (0.25) | 0.549 |
|  | hypertension × married or living with partner × pandemic | 0.93 (0.49-1.77) | -0.07 (0.33) | 0.824 |
|  | hypertension × pandemic | 1.15 (0.66-1.99) | 0.14 (0.28) | 0.631 |
|  | non-hypertension × divorced, separated or widowed × pandemic | 0.86 (0.52-1.44) | -0.15 (0.26) | 0.578 |
|  | non-hypertension × married or living with partner × pandemic | 0.95 (0.59-1.54) | -0.05 (0.24) | 0.848 |
|  | hypertension × high active | 1.39 (0.81-2.39) | 0.33 (0.28) | 0.248 |
|  | hypertension × high active × pandemic | 0.98 (0.52-1.85) | -0.02 (0.32) | 0.958 |
|  | hypertension × moderate active | 0.78 (0.45-1.36) | -0.24 (0.28) | 0.391 |
|  | hypertension × moderate active × pandemic | 1.92 (0.92-4.01) | 0.65 (0.38) | 0.096 |
|  | hypertension × pandemic | 0.94 (0.63-1.42) | -0.06 (0.21) | 0.787 |
|  | non-hypertension × high active × pandemic | 1.09 (0.72-1.65) | 0.09 (0.21) | 0.677 |
|  | non-hypertension × moderate active × pandemic | 0.77 (0.46-1.30) | -0.26 (0.27) | 0.341 |
|  | hypertension × high income | 0.78 (0.53-1.16) | -0.25 (0.20) | 0.234 |
|  | hypertension × high income × pandemic | 2.73 (1.17-6.41) | 1.01 (0.44) | 0.03 |
|  | hypertension × middle income | 0.84 (0.51-1.38) | -0.17 (0.25) | 0.5 |
|  | hypertension × middle income × pandemic | 1.93 (0.85-4.39) | 0.66 (0.42) | 0.128 |
|  | hypertension × pandemic | 0.52 (0.26-1.04) | -0.66 (0.36) | 0.075 |
|  | non-hypertension × high income × pandemic | 0.79 (0.47-1.33) | -0.23 (0.27) | 0.386 |
|  | non-hypertension × middle income × pandemic | 0.68 (0.46-1.00) | -0.39 (0.20) | 0.064 |
|  | hypertension × pandemic | 0.75 (0.30-1.87) | -0.29 (0.46) | 0.544 |
|  | hypertension × sleep hours (weekdays): 6-8h | 0.69 (0.33-1.44) | -0.38 (0.38) | 0.33 |
|  | hypertension × sleep hours (weekdays): 6-8h × pandemic | 1.69 (0.61-4.70) | 0.53 (0.52) | 0.322 |
|  | hypertension × sleep hours (weekdays)≥8h | 1.08 (0.51-2.28) | 0.08 (0.38) | 0.843 |
|  | hypertension × sleep hours (weekdays)≥8h × pandemic | 1.24 (0.42-3.68) | 0.22 (0.55) | 0.701 |
|  | non-hypertension × sleep hours (weekdays): 6-8h × pandemic | 1.00 (0.55-1.81) | -0.00 (0.31) | 0.991 |
|  | non-hypertension × sleep hours (weekdays)≥8h × pandemic | 1.35 (0.65-2.80) | 0.30 (0.37) | 0.426 |
|  | hypertension × pandemic | 1.03 (0.61-1.76) | 0.03 (0.27) | 0.904 |
|  | hypertension × sleep hours (weekends): 6-8h | 0.73 (0.44-1.21) | -0.31 (0.26) | 0.232 |
|  | hypertension × sleep hours (weekends): 6-8h × pandemic | 1.47 (0.72-3.00) | 0.39 (0.36) | 0.296 |
|  | hypertension × sleep hours (weekends)≥8h | 0.90 (0.52-1.54) | -0.11 (0.28) | 0.692 |
|  | hypertension × sleep hours (weekends)≥8h × pandemic | 0.89 (0.44-1.82) | -0.11 (0.37) | 0.756 |
|  | non-hypertension × sleep hours (weekends): 6-8h × pandemic | 0.71 (0.43-1.17) | -0.34 (0.26) | 0.194 |
|  | non-hypertension × sleep hours (weekends)≥8h × pandemic | 1.12 (0.76-1.65) | 0.11 (0.20) | 0.585 |
|  | hypertension × current smoker | 1.16 (0.73-1.83) | 0.14 (0.23) | 0.544 |
|  | hypertension × current smoker × pandemic | 0.40 (0.21-0.77) | -0.90 (0.33) | 0.011 |
|  | hypertension × ex-smoker | 0.64 (0.44-0.94) | -0.44 (0.19) | 0.033 |
|  | hypertension × ex-smoker × pandemic | 1.36 (0.83-2.24) | 0.31 (0.25) | 0.231 |
|  | hypertension × pandemic | 1.10 (0.77-1.56) | 0.10 (0.18) | 0.6 |
|  | non-hypertension × current smoker × pandemic | 1.96 (1.32-2.91) | 0.67 (0.20) | 0.003 |
|  | non-hypertension × ex-smoker × pandemic | 0.84 (0.56-1.25) | -0.18 (0.20) | 0.397 |
|  | non-obese × full-time employee × pandemic | 0.86 (0.59-1.25) | -0.15 (0.19) | 0.447 |
|  | non-obese × part-time employee × pandemic | 1.18 (0.70-1.99) | 0.17 (0.27) | 0.537 |
|  | obesity × full-time employee | 1.29 (0.87-1.90) | 0.25 (0.20) | 0.217 |
|  | obesity × full-time employee × pandemic | 0.91 (0.52-1.59) | -0.10 (0.29) | 0.735 |
|  | obesity × pandemic | 1.13 (0.74-1.72) | 0.12 (0.22) | 0.573 |
|  | obesity × part-time employee | 1.53 (0.94-2.50) | 0.43 (0.25) | 0.104 |
|  | obesity × part-time employee × pandemic | 0.76 (0.36-1.61) | -0.28 (0.39) | 0.481 |
|  | non-obese × alcohol consumption × pandemic | 1.17 (1.00-1.38) | 0.16 (0.08) | 0.059 |
|  | obesity × alcohol consumption | 1.03 (0.91-1.18) | 0.03 (0.07) | 0.633 |
|  | obesity × alcohol consumption × pandemic | 0.89 (0.66-1.20) | -0.12 (0.15) | 0.452 |
|  | obesity × pandemic | 0.98 (0.71-1.35) | -0.02 (0.16) | 0.897 |
|  | non-obese × born outside the US × pandemic | 1.01 (0.67-1.53) | 0.01 (0.21) | 0.964 |
|  | obesity × born outside the US | 1.04 (0.74-1.47) | 0.04 (0.18) | 0.825 |
|  | obesity × born outside the US × pandemic | 0.95 (0.47-1.94) | -0.05 (0.36) | 0.896 |
|  | obesity × pandemic | 1.01 (0.73-1.40) | 0.01 (0.17) | 0.943 |
|  | non-obese × CDAI × pandemic | 1.03 (0.84-1.25) | 0.03 (0.10) | 0.787 |
|  | obesity × CDAI | 1.09 (0.92-1.30) | 0.09 (0.09) | 0.328 |
|  | obesity × CDAI × pandemic | 0.85 (0.64-1.14) | -0.16 (0.15) | 0.297 |
|  | obesity × pandemic | 1.02 (0.76-1.35) | 0.02 (0.14) | 0.912 |
|  | non-obese × depression symptoms × pandemic | 0.70 (0.43-1.14) | -0.36 (0.25) | 0.164 |
|  | obesity × depression symptoms | 0.69 (0.45-1.06) | -0.37 (0.22) | 0.104 |
|  | obesity × depression symptoms × pandemic | 1.45 (0.73-2.87) | 0.37 (0.35) | 0.298 |
|  | obesity × pandemic | 0.95 (0.70-1.28) | -0.05 (0.15) | 0.722 |
|  | non-obese × DII × pandemic | 0.97 (0.79-1.20) | -0.03 (0.10) | 0.81 |
|  | obesity × DII | 0.89 (0.73-1.08) | -0.12 (0.10) | 0.236 |
|  | obesity × DII × pandemic | 1.25 (0.93-1.67) | 0.22 (0.15) | 0.148 |
|  | obesity × pandemic | 1.00 (0.76-1.33) | 0.00 (0.14) | 0.976 |
|  | non-obese × college graduate or above × pandemic | 0.72 (0.44-1.18) | -0.32 (0.25) | 0.205 |
|  | non-obese × some college × pandemic | 0.97 (0.62-1.52) | -0.03 (0.23) | 0.893 |
|  | obesity × college graduate or above | 1.98 (1.03-3.79) | 0.68 (0.33) | 0.051 |
|  | obesity × college graduate or above × pandemic | 1.19 (0.55-2.61) | 0.18 (0.40) | 0.662 |
|  | obesity × pandemic | 0.90 (0.54-1.49) | -0.11 (0.26) | 0.676 |
|  | obesity × some college | 1.31 (0.76-2.27) | 0.27 (0.28) | 0.344 |
|  | obesity × some college × pandemic | 0.99 (0.47-2.07) | -0.01 (0.38) | 0.97 |
|  | non-obese × health insurance × pandemic | 0.84 (0.49-1.45) | -0.17 (0.28) | 0.536 |
|  | obesity × health insurance | 0.96 (0.59-1.56) | -0.05 (0.25) | 0.856 |
|  | obesity × health insurance × pandemic | 1.65 (0.67-4.06) | 0.50 (0.46) | 0.282 |
|  | obesity × pandemic | 0.64 (0.28-1.43) | -0.45 (0.41) | 0.285 |
|  | non-obese × divorced, separated or widowed × pandemic | 0.98 (0.53-1.81) | -0.02 (0.31) | 0.943 |
|  | non-obese × married or living with partner × pandemic | 1.08 (0.64-1.81) | 0.08 (0.26) | 0.776 |
|  | obesity × divorced, separated or widowed | 0.61 (0.31-1.23) | -0.49 (0.36) | 0.184 |
|  | obesity × divorced, separated or widowed × pandemic | 0.78 (0.34-1.79) | -0.25 (0.42) | 0.559 |
|  | obesity × married or living with partner | 0.84 (0.41-1.72) | -0.18 (0.37) | 0.635 |
|  | obesity × married or living with partner × pandemic | 0.69 (0.29-1.61) | -0.38 (0.44) | 0.395 |
|  | obesity × pandemic | 1.34 (0.60-3.01) | 0.30 (0.41) | 0.48 |
|  | non-obese × high active × pandemic | 1.30 (0.84-2.01) | 0.26 (0.22) | 0.252 |
|  | non-obese × moderate active × pandemic | 1.20 (0.77-1.88) | 0.18 (0.23) | 0.434 |
|  | obesity × high active | 1.63 (1.06-2.50) | 0.49 (0.22) | 0.035 |
|  | obesity × high active × pandemic | 0.67 (0.37-1.21) | -0.40 (0.30) | 0.195 |
|  | obesity × moderate active | 1.70 (1.01-2.87) | 0.53 (0.27) | 0.056 |
|  | obesity × moderate active × pandemic | 0.67 (0.33-1.38) | -0.40 (0.37) | 0.29 |
|  | obesity × pandemic | 1.18 (0.84-1.65) | 0.16 (0.17) | 0.353 |
|  | non-obese × high income × pandemic | 1.00 (0.58-1.71) | -0.00 (0.28) | 0.993 |
|  | non-obese × middle income × pandemic | 0.77 (0.46-1.31) | -0.26 (0.27) | 0.351 |
|  | obesity × high income | 1.01 (0.55-1.84) | 0.01 (0.31) | 0.983 |
|  | obesity × high income × pandemic | 1.46 (0.58-3.70) | 0.38 (0.47) | 0.431 |
|  | obesity × middle income | 0.76 (0.44-1.30) | -0.27 (0.27) | 0.328 |
|  | obesity × middle income × pandemic | 1.32 (0.53-3.30) | 0.28 (0.47) | 0.56 |
|  | obesity × pandemic | 0.78 (0.35-1.76) | -0.25 (0.41) | 0.556 |
|  | non-obese × sleep hours (weekdays): 6-8h × pandemic | 1.70 (0.98-2.92) | 0.53 (0.28) | 0.07 |
|  | non-obese × sleep hours (weekdays)≥8h × pandemic | 2.13 (1.08-4.19) | 0.76 (0.35) | 0.039 |
|  | obesity × pandemic | 1.90 (0.89-4.04) | 0.64 (0.39) | 0.11 |
|  | obesity × sleep hours (weekdays): 6-8h | 1.42 (0.87-2.32) | 0.35 (0.25) | 0.173 |
|  | obesity × sleep hours (weekdays): 6-8h × pandemic | 0.54 (0.20-1.46) | -0.62 (0.51) | 0.237 |
|  | obesity × sleep hours (weekdays)≥8h | 1.74 (0.92-3.27) | 0.55 (0.32) | 0.1 |
|  | obesity × sleep hours (weekdays)≥8h × pandemic | 0.48 (0.19-1.17) | -0.74 (0.46) | 0.121 |
|  | non-obese × sleep hours (weekends): 6-8h × pandemic | 0.98 (0.56-1.72) | -0.02 (0.29) | 0.938 |
|  | non-obese × sleep hours (weekends)≥8h × pandemic | 1.21 (0.79-1.85) | 0.19 (0.22) | 0.392 |
|  | obesity × pandemic | 1.24 (0.75-2.04) | 0.21 (0.26) | 0.416 |
|  | obesity × sleep hours (weekends): 6-8h | 0.85 (0.44-1.64) | -0.16 (0.33) | 0.642 |
|  | obesity × sleep hours (weekends): 6-8h × pandemic | 0.73 (0.35-1.54) | -0.31 (0.38) | 0.421 |
|  | obesity × sleep hours (weekends)≥8h | 1.33 (0.84-2.09) | 0.28 (0.23) | 0.232 |
|  | obesity × sleep hours (weekends)≥8h × pandemic | 0.79 (0.45-1.38) | -0.24 (0.29) | 0.414 |
|  | non-obese × current smoker × pandemic | 1.57 (1.00-2.46) | 0.45 (0.23) | 0.062 |
|  | non-obese × ex-smoker × pandemic | 1.02 (0.63-1.63) | 0.02 (0.24) | 0.947 |
|  | obesity × current smoker | 0.77 (0.47-1.28) | -0.26 (0.26) | 0.33 |
|  | obesity × current smoker × pandemic | 0.72 (0.33-1.60) | -0.32 (0.41) | 0.434 |
|  | obesity × ex-smoker | 0.83 (0.51-1.36) | -0.18 (0.25) | 0.471 |
|  | obesity × ex-smoker × pandemic | 0.92 (0.49-1.72) | -0.08 (0.32) | 0.796 |
|  | obesity × pandemic | 1.07 (0.74-1.54) | 0.06 (0.19) | 0.736 |
|  | depression symptoms ×sleep hours (weekdays) <6h × pandemic | 1.18 (0.45-3.10) | 0.17 (0.49) | 0.739 |
|  | sleep hours (weekdays): 6-8h × depression symptoms | 1.19 (0.51-2.79) | 0.18 (0.43) | 0.686 |
|  | sleep hours (weekdays): 6-8h × depression symptoms × pandemic | 0.85 (0.32-2.28) | -0.17 (0.50) | 0.746 |
|  | sleep hours (weekdays): 6-8h × pandemic | 1.33 (0.77-2.29) | 0.28 (0.28) | 0.319 |
|  | sleep hours (weekdays)≥8h × depression symptoms | 1.29 (0.59-2.82) | 0.26 (0.40) | 0.528 |
|  | sleep hours (weekdays)≥8h × depression symptoms × pandemic | 0.58 (0.22-1.53) | -0.54 (0.49) | 0.282 |
|  | sleep hours (weekdays)≥8h × pandemic | 1.74 (0.85-3.56) | 0.55 (0.37) | 0.145 |
|  | multimorbidity × men × pre-pandemic | 0.72 (0.55-0.94) | -0.33 (0.14) | 0.025 |
|  | multimorbidity × men × pandemic | 1.31 (0.85-2.02) | 0.27 (0.22) | 0.235 |
|  | multimorbidity × women × pandemic | 0.88 (0.66-1.18) | -0.13 (0.15) | 0.405 |
|  | non-multimorbidity × men × pandemic | 0.79 (0.61-1.01) | -0.24 (0.13) | 0.074 |
|  | multimorbidity × pandemic | 1.02 (0.79-1.31) | 0.02 (0.13) | 0.901 |
|  | multimorbidity × full-time employee × pre-pandemic | 1.17 (0.81-1.67) | 0.15 (0.18) | 0.409 |
|  | multimorbidity × full-time employee × pandemic | 0.84 (0.51-1.38) | -0.18 (0.25) | 0.494 |
|  | multimorbidity × not-employed × pandemic | 1.12 (0.79-1.60) | 0.12 (0.18) | 0.533 |
|  | multimorbidity × part-time employee × pre-pandemic | 1.38 (0.77-2.49) | 0.32 (0.30) | 0.294 |
|  | multimorbidity × part-time employee × pandemic | 0.65 (0.29-1.44) | -0.44 (0.41) | 0.297 |
|  | non-multimorbidity × full-time employee × pandemic | 0.89 (0.63-1.25) | -0.12 (0.17) | 0.491 |
|  | part-time employee × pandemic | 1.22 (0.74-2.02) | 0.20 (0.26) | 0.437 |
|  | multimorbidity × alcohol consumption × pre-pandemic | 1.09 (0.91-1.31) | 0.09 (0.09) | 0.345 |
|  | multimorbidity × alcohol consumption × pandemic | 0.81 (0.62-1.05) | -0.22 (0.13) | 0.118 |
|  | non-multimorbidity × alcohol consumption × pandemic | 1.21 (1.05-1.41) | 0.19 (0.07) | 0.015 |
|  | multimorbidity × born outside the US × pre-pandemic | 0.79 (0.48-1.29) | -0.23 (0.25) | 0.357 |
|  | multimorbidity × born outside the US × pandemic | 1.45 (0.73-2.85) | 0.37 (0.35) | 0.298 |
|  | multimorbidity × US-born × pandemic | 0.96 (0.72-1.28) | -0.04 (0.15) | 0.798 |
|  | non-multimorbidity × born outside the US × pandemic | 0.89 (0.61-1.31) | -0.12 (0.20) | 0.557 |
|  | multimorbidity × CDAI × pre-pandemic | 1.08 (0.93-1.27) | 0.08 (0.08) | 0.33 |
|  | multimorbidity × CDAI × pandemic | 1.03 (0.82-1.30) | 0.03 (0.12) | 0.789 |
|  | non-multimorbidity × CDAI × pandemic | 0.97 (0.83-1.13) | -0.03 (0.08) | 0.673 |
|  | multimorbidity × depression symptoms × pre-pandemic | 1.38 (0.91-2.11) | 0.32 (0.22) | 0.143 |
|  | multimorbidity × depression symptoms × pandemic | 0.61 (0.33-1.12) | -0.50 (0.31) | 0.124 |
|  | multimorbidity × no/minimal depression × pandemic | 1.25 (0.93-1.68) | 0.22 (0.15) | 0.158 |
|  | non-multimorbidity × depression symptoms × pandemic | 1.09 (0.72-1.65) | 0.08 (0.21) | 0.698 |
|  | multimorbidity × DII × pre-pandemic | 0.95 (0.74-1.21) | -0.05 (0.12) | 0.667 |
|  | multimorbidity × DII × pandemic | 0.90 (0.68-1.19) | -0.11 (0.14) | 0.463 |
|  | non-multimorbidity × DII × pandemic | 1.10 (0.94-1.28) | 0.09 (0.08) | 0.255 |
|  | multimorbidity × college graduate or above × pre-pandemic | 1.18 (0.68-2.06) | 0.17 (0.28) | 0.56 |
|  | multimorbidity × college graduate or above × pandemic | 1.43 (0.64-3.17) | 0.35 (0.41) | 0.394 |
|  | multimorbidity × less than college × pandemic | 0.81 (0.49-1.34) | -0.22 (0.26) | 0.412 |
|  | multimorbidity × some college × pre-pandemic | 0.76 (0.46-1.25) | -0.28 (0.25) | 0.29 |
|  | multimorbidity × some college × pandemic | 1.15 (0.61-2.16) | 0.14 (0.32) | 0.663 |
|  | non-multimorbidity × college graduate or above × pandemic | 0.66 (0.40-1.08) | -0.42 (0.25) | 0.108 |
|  | non-multimorbidity × some college × pandemic | 0.88 (0.56-1.37) | -0.13 (0.23) | 0.579 |
|  | multimorbidity × health insurance × pre-pandemic | 1.00 (0.61-1.65) | 0.00 (0.25) | 0.986 |
|  | multimorbidity × health insurance × pandemic | 0.53 (0.21-1.33) | -0.64 (0.47) | 0.188 |
|  | multimorbidity × no health insurance × pandemic | 1.86 (0.74-4.66) | 0.62 (0.47) | 0.198 |
|  | non-multimorbidity × health insurance × pandemic | 1.29 (0.74-2.25) | 0.25 (0.28) | 0.378 |
|  | multimorbidity × divorced, separated or widowed × pre-pandemic | 0.54 (0.28-1.04) | -0.61 (0.33) | 0.077 |
|  | multimorbidity × divorced, separated or widowed × pandemic | 0.94 (0.43-2.08) | -0.06 (0.40) | 0.885 |
|  | multimorbidity × married or living with partner × pre-pandemic | 0.91 (0.52-1.58) | -0.10 (0.28) | 0.734 |
|  | multimorbidity × married or living with partner × pandemic | 0.84 (0.43-1.65) | -0.18 (0.35) | 0.61 |
|  | multimorbidity × never married × pandemic | 1.20 (0.64-2.25) | 0.18 (0.32) | 0.585 |
|  | non-multimorbidity × divorced, separated or widowed × pandemic | 0.88 (0.49-1.57) | -0.13 (0.30) | 0.658 |
|  | non-multimorbidity × married or living with partner × pandemic | 1.00 (0.65-1.53) | -0.00 (0.22) | 1 |
|  | multimorbidity × high active × pre-pandemic | 1.35 (0.79-2.29) | 0.30 (0.27) | 0.283 |
|  | multimorbidity × high active × pandemic | 0.70 (0.36-1.37) | -0.35 (0.34) | 0.311 |
|  | multimorbidity × moderate active × pre-pandemic | 0.84 (0.50-1.41) | -0.17 (0.26) | 0.513 |
|  | multimorbidity × moderate active × pandemic | 1.51 (0.71-3.21) | 0.41 (0.38) | 0.291 |
|  | multimorbidity × low active × pandemic | 1.05 (0.77-1.43) | 0.05 (0.16) | 0.772 |
|  | non-multimorbidity × high active × pandemic | 1.22 (0.84-1.79) | 0.20 (0.19) | 0.309 |
|  | non-multimorbidity × moderate active × pandemic | 0.87 (0.52-1.47) | -0.14 (0.27) | 0.614 |
|  | multimorbidity × high income × pre-pandemic | 0.77 (0.41-1.43) | -0.26 (0.32) | 0.419 |
|  | multimorbidity × high income × pandemic | 2.59 (1.17-5.71) | 0.95 (0.40) | 0.028 |
|  | multimorbidity × middle income × pre-pandemic | 0.82 (0.40-1.69) | -0.20 (0.37) | 0.6 |
|  | multimorbidity × middle income × pandemic | 2.24 (1.00-5.00) | 0.81 (0.41) | 0.061 |
|  | multimorbidity × low income × pandemic | 0.48 (0.23-0.99) | -0.73 (0.37) | 0.06 |
|  | non-multimorbidity × high income × pandemic | 0.80 (0.52-1.23) | -0.23 (0.22) | 0.312 |
|  | non-multimorbidity × middle income × pandemic | 0.63 (0.42-0.94) | -0.47 (0.21) | 0.036 |
|  | multimorbidity × sleep hours (weekdays) <6h × pandemic | 1.17 (0.40-3.43) | 0.16 (0.55) | 0.775 |
|  | multimorbidity × sleep hours (weekdays): 6-8h × pre-pandemic | 1.03 (0.38-2.81) | 0.03 (0.51) | 0.959 |
|  | multimorbidity × sleep hours (weekdays): 6-8h × pre-pandemic | 1.21 (0.44-3.32) | 0.19 (0.52) | 0.716 |
|  | multimorbidity × sleep hours (weekdays): 6-8h × pandemic | 1.04 (0.33-3.28) | 0.04 (0.59) | 0.951 |
|  | multimorbidity × sleep hours (weekdays): 6-8h × pandemic | 0.73 (0.22-2.38) | -0.32 (0.61) | 0.604 |
|  | sleep hours (weekdays): 6-8h × pandemic | 1.28 (0.63-2.58) | 0.24 (0.36) | 0.504 |
|  | sleep hours (weekdays): 6-8h × pandemic | 1.75 (0.75-4.08) | 0.56 (0.43) | 0.206 |
|  | multimorbidity × sleep hours (weekends) <6h × pandemic | 1.08 (0.55-2.13) | 0.08 (0.35) | 0.828 |
|  | multimorbidity × sleep hours (weekends) ≥ 8h × pre-pandemic | 0.88 (0.46-1.68) | -0.13 (0.33) | 0.706 |
|  | multimorbidity × sleep hours (weekends) ≥ 8h × pandemic | 0.85 (0.37-1.92) | -0.17 (0.42) | 0.694 |
|  | multimorbidity × sleep hours (weekends): 6-8h × pre-pandemic | 0.83 (0.47-1.49) | -0.18 (0.30) | 0.545 |
|  | multimorbidity × sleep hours (weekends): 6-8h × pandemic | 1.14 (0.53-2.49) | 0.14 (0.40) | 0.737 |
|  | sleep hours (weekends) ≥ 8h × pandemic | 1.16 (0.70-1.93) | 0.15 (0.26) | 0.578 |
|  | sleep hours (weekends): 6-8h × pandemic | 0.81 (0.47-1.39) | -0.21 (0.27) | 0.454 |
|  | multimorbidity × current smoker × pre-pandemic | 1.58 (0.90-2.77) | 0.46 (0.29) | 0.125 |
|  | multimorbidity × current smoker × pandemic | 0.31 (0.14-0.66) | -1.18 (0.39) | 0.006 |
|  | multimorbidity × never-smoker × pandemic | 1.06 (0.77-1.46) | 0.06 (0.16) | 0.715 |
|  | multimorbidity × ex-smoker × pre-pandemic | 0.75 (0.51-1.11) | -0.29 (0.20) | 0.164 |
|  | multimorbidity × ex-smoker × pandemic | 1.54 (0.95-2.50) | 0.43 (0.25) | 0.091 |
|  | non-multimorbidity × current smoker × pandemic | 2.31 (1.41-3.78) | 0.84 (0.25) | 0.003 |
|  | non-multimorbidity × ex-smoker × pandemic | 0.80 (0.51-1.24) | -0.23 (0.23) | 0.321 |
|  | men × age × multimorbidity × pandemic | 1.00 (0.96-1.04) | -0.00 (0.02) | 0.879 |
| T2D | ≥60 years × less than college × pandemic | 1.02 (0.34-3.12) | 0.02 (0.57) | 0.967 |
|  | ≥60 years × never married × pandemic | 1.32 (0.41-4.22) | 0.27 (0.59) | 0.65 |
|  | ≥60 years × no health insurance × pandemic | 0.58 (0.13-2.68) | -0.54 (0.78) | 0.496 |
|  | ≥60 years × sleep hours (weekdays) <6h × pandemic | 0.32 (0.06-1.73) | -1.14 (0.86) | 0.203 |
|  | ≥60 years × US-born × pandemic | 0.60 (0.29-1.21) | -0.52 (0.36) | 0.166 |
|  | 40-59 years × less than college × pandemic | 0.87 (0.28-2.73) | -0.14 (0.58) | 0.813 |
|  | 40-59 years × never married × pandemic | 1.12 (0.28-4.44) | 0.12 (0.70) | 0.87 |
|  | 40-59 years × no health insurance × pandemic | 0.70 (0.16-3.00) | -0.36 (0.75) | 0.631 |
|  | 40-59 years × sleep hours (weekdays) <6h × pandemic | 0.72 (0.13-3.98) | -0.33 (0.87) | 0.709 |
|  | 40-59 years × US-born × pandemic | 0.70 (0.28-1.78) | -0.36 (0.48) | 0.459 |
|  | alcohol consumption × ≥60 years | 2.07 (0.68-6.36) | 0.73 (0.57) | 0.216 |
|  | alcohol consumption × ≥60 years × pandemic | 0.31 (0.08-1.28) | -1.17 (0.72) | 0.119 |
|  | alcohol consumption × 40-59 years | 1.57 (0.52-4.75) | 0.45 (0.57) | 0.434 |
|  | alcohol consumption × 40-59 years × pandemic | 0.83 (0.21-3.20) | -0.19 (0.69) | 0.785 |
|  | alcohol consumption × pandemic | 1.71 (0.43-6.74) | 0.54 (0.70) | 0.452 |
|  | born outside the US × ≥60 years | 0.36 (0.17-0.77) | -1.02 (0.38) | 0.014 |
|  | born outside the US × ≥60 years × pandemic | 8.41 (1.41-50.31) | 2.13 (0.91) | 0.029 |
|  | born outside the US × 40-59 years | 0.27 (0.12-0.60) | -1.31 (0.40) | 0.004 |
|  | born outside the US × 40-59 years × pandemic | 8.86 (1.47-53.43) | 2.18 (0.92) | 0.026 |
|  | born outside the US × pandemic | 0.11 (0.02-0.61) | -2.17 (0.85) | 0.018 |
|  | CDAI × ≥60 years | 0.76 (0.56-1.03) | -0.28 (0.15) | 0.086 |
|  | CDAI × ≥60 years × pandemic | 2.38 (1.42-3.97) | 0.87 (0.26) | 0.003 |
|  | CDAI × 40-59 years | 0.77 (0.59-1.01) | -0.26 (0.14) | 0.072 |
|  | CDAI × 40-59 years × pandemic | 2.80 (1.58-4.94) | 1.03 (0.29) | 0.002 |
|  | CDAI × pandemic | 0.46 (0.28-0.76) | -0.78 (0.25) | 0.005 |
|  | college graduate or above × ≥60 years | 0.95 (0.36-2.46) | -0.06 (0.49) | 0.909 |
|  | college graduate or above × ≥60 years × pandemic | 1.48 (0.24-9.02) | 0.39 (0.92) | 0.674 |
|  | college graduate or above × 40-59 years | 0.74 (0.24-2.28) | -0.30 (0.57) | 0.607 |
|  | college graduate or above × 40-59 years × pandemic | 1.68 (0.32-8.95) | 0.52 (0.85) | 0.551 |
|  | college graduate or above × pandemic | 0.59 (0.12-2.84) | -0.53 (0.80) | 0.516 |
|  | current smoker × ≥60 years | 1.43 (0.58-3.51) | 0.36 (0.46) | 0.448 |
|  | current smoker × ≥60 years × pandemic | 0.35 (0.08-1.62) | -1.04 (0.78) | 0.199 |
|  | current smoker × 40-59 years | 1.22 (0.63-2.39) | 0.20 (0.34) | 0.564 |
|  | current smoker × 40-59 years × pandemic | 0.57 (0.12-2.81) | -0.56 (0.81) | 0.498 |
|  | current smoker × pandemic | 2.90 (0.62-13.63) | 1.06 (0.79) | 0.196 |
|  | depression symptoms × ≥60 years | 1.21 (0.58-2.55) | 0.19 (0.38) | 0.619 |
|  | depression symptoms × ≥60 years × pandemic | 0.77 (0.27-2.20) | -0.26 (0.53) | 0.632 |
|  | depression symptoms × 40-59 years | 0.94 (0.42-2.10) | -0.06 (0.41) | 0.886 |
|  | depression symptoms × 40-59 years × pandemic | 1.37 (0.41-4.58) | 0.32 (0.61) | 0.61 |
|  | depression symptoms × pandemic | 1.21 (0.43-3.36) | 0.19 (0.52) | 0.721 |
|  | DII × ≥60 years | 1.45 (0.97-2.17) | 0.37 (0.21) | 0.084 |
|  | DII × ≥60 years × pandemic | 0.38 (0.22-0.66) | -0.97 (0.28) | 0.002 |
|  | DII × 40-59 years | 1.61 (1.11-2.35) | 0.48 (0.19) | 0.02 |
|  | DII × pandemic | 2.47 (1.47-4.16) | 0.91 (0.27) | 0.002 |
|  | divorced, separated or widowed × ≥60 years | 2.39 (0.24-24.21) | 0.87 (1.18) | 0.47 |
|  | divorced, separated or widowed × 40-59 years | 1.63 (0.16-16.87) | 0.49 (1.19) | 0.689 |
|  | ex-smoker × ≥60 years | 0.99 (0.25-3.94) | -0.01 (0.70) | 0.993 |
|  | ex-smoker × ≥60 years × pandemic | 0.34 (0.06-1.90) | -1.08 (0.88) | 0.235 |
|  | ex-smoker × 40-59 years | 0.76 (0.21-2.81) | -0.27 (0.67) | 0.687 |
|  | ex-smoker × 40-59 years × pandemic | 0.41 (0.06-2.74) | -0.89 (0.97) | 0.371 |
|  | ex-smoker × pandemic | 2.64 (0.52-13.52) | 0.97 (0.83) | 0.26 |
|  | full-time employee × ≥60 years | 2.59 (1.00-6.72) | 0.95 (0.49) | 0.067 |
|  | full-time employee × ≥60 years × pandemic | 0.37 (0.06-2.45) | -0.98 (0.96) | 0.32 |
|  | full-time employee × 40-59 years | 2.50 (0.97-6.47) | 0.92 (0.48) | 0.076 |
|  | full-time employee × 40-59 years × pandemic | 0.27 (0.04-1.76) | -1.31 (0.96) | 0.189 |
|  | full-time employee × pandemic | 2.33 (0.40-13.64) | 0.84 (0.90) | 0.362 |
|  | health insurance × ≥60 years | 0.83 (0.32-2.17) | -0.19 (0.49) | 0.706 |
|  | health insurance × ≥60 years × pandemic | 1.56 (0.24-10.18) | 0.45 (0.96) | 0.644 |
|  | health insurance × 40-59 years | 1.34 (0.47-3.84) | 0.29 (0.54) | 0.595 |
|  | health insurance × 40-59 years × pandemic | 1.56 (0.20-11.99) | 0.45 (1.04) | 0.673 |
|  | health insurance × pandemic | 0.81 (0.18-3.72) | -0.21 (0.78) | 0.788 |
|  | high active × ≥60 years | 1.03 (0.36-2.96) | 0.03 (0.54) | 0.961 |
|  | high active × ≥60 years × pandemic | 0.65 (0.12-3.43) | -0.43 (0.85) | 0.618 |
|  | high active × 40-59 years | 1.07 (0.40-2.86) | 0.07 (0.50) | 0.89 |
|  | high active × 40-59 years × pandemic | 0.78 (0.17-3.51) | -0.25 (0.77) | 0.752 |
|  | high active × pandemic | 1.52 (0.28-8.14) | 0.42 (0.86) | 0.631 |
|  | high income × ≥60 years | 1.23 (0.39-3.91) | 0.21 (0.59) | 0.731 |
|  | high income × ≥60 years × pandemic | 2.43 (0.45-13.18) | 0.89 (0.86) | 0.319 |
|  | high income × 40-59 years | 1.27 (0.36-4.49) | 0.24 (0.64) | 0.71 |
|  | high income × 40-59 years × pandemic | 1.76 (0.28-11.16) | 0.57 (0.94) | 0.556 |
|  | high income × pandemic | 0.35 (0.06-2.18) | -1.04 (0.93) | 0.278 |
|  | low active × ≥60 years × pandemic | 0.91 (0.39-2.14) | -0.09 (0.43) | 0.84 |
|  | low active × 40-59 years × pandemic | 0.98 (0.42-2.29) | -0.02 (0.43) | 0.957 |
|  | low income × ≥60 years × pandemic | 0.84 (0.29-2.44) | -0.18 (0.55) | 0.752 |
|  | low income × 40-59 years × pandemic | 1.01 (0.33-3.08) | 0.01 (0.57) | 0.987 |
|  | married or living with partner × ≥60 years | 1.70 (0.66-4.36) | 0.53 (0.48) | 0.284 |
|  | married or living with partner × ≥60 years × pandemic | 0.63 (0.14-2.88) | -0.46 (0.77) | 0.561 |
|  | married or living with partner × 40-59 years | 1.26 (0.64-2.48) | 0.23 (0.34) | 0.506 |
|  | married or living with partner × 40-59 years × pandemic | 0.79 (0.11-5.50) | -0.24 (0.99) | 0.815 |
|  | married or living with partner × pandemic | 1.05 (0.29-3.78) | 0.05 (0.65) | 0.94 |
|  | middle income × ≥60 years | 1.38 (0.73-2.62) | 0.32 (0.33) | 0.338 |
|  | middle income × ≥60 years × pandemic | 0.75 (0.26-2.15) | -0.29 (0.54) | 0.598 |
|  | middle income × 40-59 years | 1.13 (0.60-2.13) | 0.12 (0.32) | 0.714 |
|  | middle income × 40-59 years × pandemic | 1.02 (0.28-3.67) | 0.02 (0.65) | 0.973 |
|  | middle income × pandemic | 1.37 (0.54-3.47) | 0.31 (0.47) | 0.516 |
|  | moderate active × ≥60 years | 0.69 (0.14-3.57) | -0.37 (0.83) | 0.667 |
|  | moderate active × ≥60 years × pandemic | 3.28 (0.28-37.95) | 1.19 (1.25) | 0.355 |
|  | moderate active × 40-59 years | 0.64 (0.13-3.19) | -0.44 (0.82) | 0.597 |
|  | moderate active × 40-59 years × pandemic | 3.84 (0.39-37.57) | 1.35 (1.16) | 0.264 |
|  | moderate active × pandemic | 0.23 (0.03-2.16) | -1.45 (1.13) | 0.218 |
|  | never-smoker × ≥60 years × pandemic | 1.35 (0.67-2.72) | 0.30 (0.36) | 0.411 |
|  | never-smoker × 40-59 years × pandemic | 1.37 (0.44-4.24) | 0.32 (0.58) | 0.59 |
|  | not-employed × ≥60 years × pandemic | 1.52 (0.47-4.92) | 0.42 (0.60) | 0.497 |
|  | not-employed × 40-59 years × pandemic | 2.32 (0.73-7.45) | 0.84 (0.59) | 0.174 |
|  | part-time employee × ≥60 years | 0.94 (0.33-2.63) | -0.06 (0.53) | 0.906 |
|  | part-time employee × ≥60 years × pandemic | 0.83 (0.13-5.13) | -0.19 (0.93) | 0.84 |
|  | part-time employee × 40-59 years | 1.09 (0.36-3.33) | 0.09 (0.57) | 0.881 |
|  | part-time employee × 40-59 years × pandemic | 0.67 (0.09-5.14) | -0.40 (1.04) | 0.705 |
|  | part-time employee × pandemic | 1.48 (0.30-7.44) | 0.39 (0.82) | 0.637 |
|  | sleep hours (weekdays) <6h × ≥60 years × pandemic | 0.53 (0.19-1.53) | -0.63 (0.54) | 0.256 |
|  | sleep hours (weekdays) <6h × 40-59 years × pandemic | 0.45 (0.13-1.57) | -0.81 (0.64) | 0.226 |
|  | sleep hours (weekdays): 6-8h × ≥60 years | 0.98 (0.34-2.84) | -0.02 (0.54) | 0.978 |
|  | sleep hours (weekdays): 6-8h × ≥60 years × pandemic | 5.82 (0.82-41.37) | 1.76 (1.00) | 0.096 |
|  | sleep hours (weekdays): 6-8h × 40-59 years | 0.51 (0.14-1.92) | -0.67 (0.68) | 0.334 |
|  | sleep hours (weekdays): 6-8h × 40-59 years × pandemic | 3.13 (0.34-29.05) | 1.14 (1.14) | 0.33 |
|  | sleep hours (weekdays): 6-8h × pandemic | 0.16 (0.03-0.97) | -1.82 (0.91) | 0.063 |
|  | sleep hours (weekdays)≥8h × ≥60 years | 1.37 (0.48-3.89) | 0.31 (0.53) | 0.564 |
|  | sleep hours (weekdays)≥8h × ≥60 years × pandemic | 1.94 (0.27-13.75) | 0.66 (1.00) | 0.518 |
|  | sleep hours (weekdays)≥8h × 40-59 years | 0.79 (0.23-2.70) | -0.23 (0.63) | 0.714 |
|  | sleep hours (weekdays)≥8h × 40-59 years × pandemic | 0.89 (0.14-5.50) | -0.12 (0.93) | 0.899 |
|  | sleep hours (weekdays)≥8h × pandemic | 0.67 (0.14-3.35) | -0.39 (0.82) | 0.636 |
|  | sleep hours (weekends): 6-8h × ≥60 years | 0.76 (0.36-1.61) | -0.27 (0.38) | 0.483 |
|  | sleep hours (weekends): 6-8h × ≥60 years × pandemic | 0.84 (0.18-4.04) | -0.17 (0.80) | 0.835 |
|  | sleep hours (weekends): 6-8h × 40-59 years | 0.56 (0.20-1.56) | -0.57 (0.52) | 0.285 |
|  | sleep hours (weekends): 6-8h × 40-59 years × pandemic | 1.86 (0.31-11.21) | 0.62 (0.92) | 0.508 |
|  | sleep hours (weekends): 6-8h × pandemic | 0.91 (0.20-4.13) | -0.10 (0.77) | 0.902 |
|  | sleep hours (weekends)≥8h × ≥60 years | 0.86 (0.41-1.79) | -0.15 (0.37) | 0.69 |
|  | sleep hours (weekends)≥8h × ≥60 years × pandemic | 4.46 (1.26-15.86) | 1.50 (0.65) | 0.034 |
|  | sleep hours (weekends)≥8h × 40-59 years | 0.64 (0.24-1.69) | -0.45 (0.50) | 0.38 |
|  | sleep hours (weekends)≥8h × 40-59 years × pandemic | 5.64 (1.25-25.51) | 1.73 (0.77) | 0.038 |
|  | sleep hours (weekends)≥8h × pandemic | 0.21 (0.06-0.70) | -1.58 (0.63) | 0.022 |
|  | some college × ≥60 years | 1.79 (0.77-4.16) | 0.58 (0.43) | 0.192 |
|  | some college × ≥60 years × pandemic | 0.50 (0.14-1.79) | -0.69 (0.65) | 0.301 |
|  | some college × 40-59 years | 0.87 (0.40-1.87) | -0.14 (0.39) | 0.725 |
|  | some college × 40-59 years × pandemic | 1.18 (0.38-3.64) | 0.16 (0.57) | 0.778 |
|  | some college × pandemic | 1.78 (0.66-4.81) | 0.58 (0.51) | 0.27 |
|  | alcohol consumption × depression symptoms | 0.37 (0.17-0.83) | -0.99 (0.41) | 0.022 |
|  | alcohol consumption × depression symptoms × pandemic | 4.79 (1.96-11.71) | 1.57 (0.46) | 0.002 |
|  | alcohol consumption × pandemic | 0.65 (0.42-1.02) | -0.43 (0.23) | 0.074 |
|  | CDAI × depression symptoms | 0.94 (0.78-1.13) | -0.06 (0.09) | 0.511 |
|  | CDAI × depression symptoms × pandemic | 1.12 (0.85-1.47) | 0.11 (0.14) | 0.419 |
|  | CDAI × alcohol consumption × pre-pandemic | 1.01 (0.87-1.16) | 0.01 (0.07) | 0.923 |
|  | CDAI × alcohol consumption × pandemic | 1.06 (0.86-1.31) | 0.06 (0.11) | 0.589 |
|  | CDAI × current smoker × pre-pandemic | 1.12 (0.77-1.63) | 0.11 (0.19) | 0.563 |
|  | CDAI × current smoker × pandemic | 0.95 (0.60-1.51) | -0.05 (0.23) | 0.843 |
|  | CDAI × ex-smoker × pre-pandemic | 0.81 (0.61-1.07) | -0.21 (0.14) | 0.155 |
|  | CDAI × ex-smoker × pandemic | 1.14 (0.69-1.89) | 0.13 (0.26) | 0.604 |
|  | CDAI × high active × pre-pandemic | 1.05 (0.80-1.36) | 0.05 (0.14) | 0.741 |
|  | CDAI × high active × pandemic | 0.75 (0.49-1.14) | -0.29 (0.21) | 0.185 |
|  | CDAI × moderate active × pre-pandemic | 0.93 (0.66-1.29) | -0.08 (0.17) | 0.658 |
|  | CDAI × moderate active × pandemic | 0.82 (0.51-1.34) | -0.19 (0.25) | 0.443 |
|  | CDAI × pandemic | 1.03 (0.82-1.30) | 0.03 (0.12) | 0.797 |
|  | current smoker × depression symptoms | 0.63 (0.35-1.15) | -0.46 (0.30) | 0.149 |
|  | current smoker × depression symptoms × pandemic | 3.17 (1.32-7.61) | 1.15 (0.45) | 0.017 |
|  | current smoker × pandemic | 1.14 (0.59-2.19) | 0.13 (0.33) | 0.699 |
|  | DII × depression symptoms | 1.02 (0.86-1.21) | 0.02 (0.09) | 0.803 |
|  | DII × depression symptoms × pandemic | 0.94 (0.72-1.23) | -0.06 (0.14) | 0.647 |
|  | DII × pandemic | 0.95 (0.78-1.16) | -0.05 (0.10) | 0.605 |
|  | DII × alcohol consumption × pandemic | 0.98 (0.76-1.25) | -0.02 (0.13) | 0.853 |
|  | DII × alcohol consumption × pre-pandemic | 1.02 (0.85-1.23) | 0.02 (0.09) | 0.84 |
|  | DII × current smoker × pandemic | 0.96 (0.57-1.63) | -0.04 (0.27) | 0.88 |
|  | DII × current smoker × pre-pandemic | 1.07 (0.70-1.64) | 0.07 (0.22) | 0.744 |
|  | DII × ex-smoker × pandemic | 1.06 (0.70-1.61) | 0.06 (0.21) | 0.795 |
|  | DII × ex-smoker × pre-pandemic | 0.96 (0.75-1.23) | -0.04 (0.13) | 0.742 |
|  | DII × high active × pandemic | 1.13 (0.78-1.63) | 0.12 (0.19) | 0.523 |
|  | DII × high active × pre-pandemic | 1.03 (0.80-1.31) | 0.03 (0.12) | 0.834 |
|  | DII × moderate active × pandemic | 1.04 (0.59-1.86) | 0.04 (0.29) | 0.889 |
|  | DII × moderate active × pre-pandemic | 1.06 (0.68-1.67) | 0.06 (0.23) | 0.787 |
|  | energy intake kcal × depression symptoms | 0.96 (0.77-1.19) | -0.04 (0.11) | 0.689 |
|  | energy intake kcal × depression symptoms × pandemic | 1.06 (0.79-1.43) | 0.06 (0.15) | 0.69 |
|  | energy intake kcal × pandemic | 1.20 (0.92-1.56) | 0.18 (0.13) | 0.189 |
|  | ex-smoker × depression symptoms | 0.94 (0.50-1.76) | -0.06 (0.32) | 0.844 |
|  | ex-smoker × depression symptoms × pandemic | 1.68 (0.73-3.90) | 0.52 (0.43) | 0.237 |
|  | ex-smoker × pandemic | 0.89 (0.54-1.48) | -0.12 (0.26) | 0.656 |
|  | high active × depression symptoms | 0.51 (0.26-1.01) | -0.67 (0.35) | 0.066 |
|  | high active × depression symptoms × pandemic | 1.73 (0.57-5.23) | 0.55 (0.57) | 0.345 |
|  | high active × pandemic | 1.10 (0.54-2.22) | 0.09 (0.36) | 0.795 |
|  | low active × depression symptoms × pandemic | 0.88 (0.57-1.36) | -0.12 (0.22) | 0.581 |
|  | moderate active × depression symptoms | 0.87 (0.43-1.73) | -0.14 (0.35) | 0.685 |
|  | moderate active × depression symptoms × pandemic | 1.73 (0.63-4.79) | 0.55 (0.52) | 0.301 |
|  | moderate active × pandemic | 0.64 (0.37-1.12) | -0.44 (0.28) | 0.13 |
|  | never-smoker × depression symptoms × pandemic | 0.69 (0.48-1.00) | -0.37 (0.19) | 0.064 |
|  | sleep hours (weekdays) <6h × depression symptoms × pandemic | 1.22 (0.56-2.69) | 0.20 (0.40) | 0.622 |
|  | sleep hours (weekends): 6-8h × depression symptoms | 1.05 (0.46-2.38) | 0.05 (0.42) | 0.909 |
|  | sleep hours (weekends): 6-8h × depression symptoms × pandemic | 0.78 (0.25-2.40) | -0.25 (0.57) | 0.667 |
|  | sleep hours (weekends): 6-8h × pandemic | 1.10 (0.64-1.88) | 0.09 (0.27) | 0.733 |
|  | sleep hours (weekends)≥8h × depression symptoms | 1.34 (0.71-2.52) | 0.29 (0.32) | 0.381 |
|  | sleep hours (weekends)≥8h × depression symptoms × pandemic | 0.79 (0.27-2.30) | -0.24 (0.55) | 0.668 |
|  | sleep hours (weekends)≥8h × pandemic | 0.92 (0.55-1.53) | -0.09 (0.26) | 0.745 |
|  | college graduate or above × divorced, separated or widowed | 0.86 (0.28-2.65) | -0.15 (0.57) | 0.797 |
|  | college graduate or above × divorced, separated or widowed × pandemic | 1.34 (0.31-5.73) | 0.29 (0.74) | 0.696 |
|  | college graduate or above × married or living with partner | 0.57 (0.19-1.72) | -0.57 (0.57) | 0.329 |
|  | college graduate or above × married or living with partner × pandemic | 1.36 (0.35-5.18) | 0.30 (0.68) | 0.662 |
|  | college graduate or above × pandemic | 0.63 (0.20-1.95) | -0.46 (0.57) | 0.435 |
|  | less than college × divorced, separated or widowed × pandemic | 0.62 (0.26-1.49) | -0.48 (0.45) | 0.296 |
|  | less than college × married or living with partner × pandemic | 0.59 (0.25-1.44) | -0.52 (0.45) | 0.265 |
|  | some college × divorced, separated or widowed | 0.89 (0.27-2.95) | -0.11 (0.61) | 0.853 |
|  | some college × divorced, separated or widowed × pandemic | 1.22 (0.22-6.64) | 0.20 (0.86) | 0.82 |
|  | some college × married or living with partner | 0.69 (0.29-1.64) | -0.37 (0.44) | 0.416 |
|  | some college × married or living with partner × pandemic | 1.54 (0.50-4.73) | 0.43 (0.57) | 0.462 |
|  | some college × pandemic | 0.91 (0.30-2.74) | -0.09 (0.56) | 0.87 |
|  | alcohol consumption × men | 2.65 (1.20-5.84) | 0.97 (0.40) | 0.023 |
|  | alcohol consumption × men × pandemic | 0.37 (0.11-1.27) | -0.98 (0.62) | 0.127 |
|  | alcohol consumption × pandemic | 2.58 (0.95-7.02) | 0.95 (0.51) | 0.075 |
|  | born outside the US × men | 0.69 (0.46-1.05) | -0.37 (0.21) | 0.094 |
|  | born outside the US × men × pandemic | 1.66 (0.83-3.33) | 0.51 (0.35) | 0.161 |
|  | born outside the US × pandemic | 0.59 (0.35-1.01) | -0.52 (0.27) | 0.065 |
|  | CDAI × men | 1.16 (0.97-1.40) | 0.15 (0.09) | 0.122 |
|  | CDAI × men × pandemic | 1.07 (0.78-1.47) | 0.07 (0.16) | 0.687 |
|  | CDAI × pandemic | 1.02 (0.79-1.33) | 0.02 (0.13) | 0.864 |
|  | college graduate or above × men | 1.44 (0.80-2.60) | 0.37 (0.30) | 0.237 |
|  | college graduate or above × men × pandemic | 1.24 (0.52-2.94) | 0.22 (0.44) | 0.628 |
|  | college graduate or above × pandemic | 0.75 (0.35-1.58) | -0.29 (0.38) | 0.454 |
|  | current smoker × men | 0.78 (0.43-1.40) | -0.25 (0.30) | 0.409 |
|  | current smoker × men × pandemic | 0.74 (0.28-1.96) | -0.31 (0.50) | 0.546 |
|  | current smoker × pandemic | 2.03 (0.92-4.50) | 0.71 (0.40) | 0.093 |
|  | depression symptoms × men | 0.58 (0.35-0.97) | -0.55 (0.26) | 0.046 |
|  | depression symptoms × men × pandemic | 1.44 (0.71-2.95) | 0.37 (0.36) | 0.324 |
|  | depression symptoms × pandemic | 0.88 (0.58-1.34) | -0.13 (0.22) | 0.561 |
|  | DII × men | 0.99 (0.82-1.18) | -0.01 (0.09) | 0.877 |
|  | DII × men × pandemic | 0.88 (0.65-1.18) | -0.13 (0.15) | 0.395 |
|  | DII × pandemic | 1.01 (0.79-1.30) | 0.01 (0.13) | 0.917 |
|  | divorced, separated or widowed × men | 1.09 (0.55-2.15) | 0.08 (0.35) | 0.812 |
|  | divorced, separated or widowed × men × pandemic | 1.86 (0.78-4.47) | 0.62 (0.45) | 0.177 |
|  | divorced, separated or widowed × pandemic | 0.57 (0.33-0.98) | -0.56 (0.28) | 0.055 |
|  | ex-smoker × men | 1.55 (0.92-2.61) | 0.44 (0.27) | 0.111 |
|  | ex-smoker × men × pandemic | 0.81 (0.36-1.85) | -0.21 (0.42) | 0.622 |
|  | ex-smoker × pandemic | 1.15 (0.60-2.21) | 0.14 (0.33) | 0.67 |
|  | full-time employee × men | 0.53 (0.35-0.83) | -0.63 (0.22) | 0.01 |
|  | full-time employee × men × pandemic | 1.73 (0.86-3.47) | 0.55 (0.35) | 0.135 |
|  | full-time employee × pandemic | 0.74 (0.51-1.07) | -0.31 (0.19) | 0.123 |
|  | health insurance × men | 3.04 (1.54-5.97) | 1.11 (0.35) | 0.003 |
|  | health insurance × men × pandemic | 0.49 (0.13-1.84) | -0.71 (0.67) | 0.301 |
|  | health insurance × pandemic | 1.56 (0.60-4.03) | 0.44 (0.48) | 0.368 |
|  | high active × men | 1.50 (0.84-2.66) | 0.40 (0.29) | 0.184 |
|  | high active × men × pandemic | 0.55 (0.22-1.38) | -0.61 (0.47) | 0.214 |
|  | high active × pandemic | 1.73 (0.95-3.16) | 0.55 (0.31) | 0.088 |
|  | high income × men | 1.21 (0.65-2.25) | 0.19 (0.32) | 0.551 |
|  | high income × men × pandemic | 1.23 (0.45-3.35) | 0.21 (0.51) | 0.687 |
|  | high income × pandemic | 0.65 (0.36-1.16) | -0.43 (0.30) | 0.158 |
|  | hypertension × men | 0.74 (0.50-1.10) | -0.30 (0.20) | 0.148 |
|  | hypertension × men × pandemic | 1.03 (0.51-2.06) | 0.03 (0.36) | 0.943 |
|  | hypertension × pandemic | 0.78 (0.50-1.23) | -0.25 (0.23) | 0.298 |
|  | low active × men × pandemic | 1.35 (0.67-2.73) | 0.30 (0.36) | 0.408 |
|  | low income × men × pandemic | 1.02 (0.42-2.50) | 0.02 (0.46) | 0.966 |
|  | men × less than college × pandemic | 1.02 (0.59-1.76) | 0.02 (0.28) | 0.934 |
|  | men × never married × pandemic | 0.69 (0.30-1.56) | -0.37 (0.42) | 0.382 |
|  | men × never-smoker × pandemic | 1.28 (0.74-2.19) | 0.24 (0.28) | 0.384 |
|  | men × no health insurance × pandemic | 2.17 (0.58-8.15) | 0.77 (0.68) | 0.262 |
|  | men × not-employed × pandemic | 0.98 (0.59-1.62) | -0.02 (0.26) | 0.933 |
|  | men × sleep hours (weekdays) <6h × pandemic | 1.17 (0.50-2.75) | 0.16 (0.44) | 0.72 |
|  | married or living with partner × men | 1.36 (0.74-2.51) | 0.31 (0.31) | 0.329 |
|  | married or living with partner × men × pandemic | 1.77 (0.78-4.01) | 0.57 (0.42) | 0.182 |
|  | married or living with partner × pandemic | 0.55 (0.35-0.87) | -0.59 (0.23) | 0.017 |
|  | middle income × men | 1.17 (0.67-2.04) | 0.15 (0.28) | 0.593 |
|  | middle income × men × pandemic | 1.21 (0.50-2.92) | 0.19 (0.45) | 0.679 |
|  | middle income × pandemic | 1.09 (0.71-1.69) | 0.09 (0.22) | 0.692 |
|  | moderate active × men | 1.83 (0.88-3.81) | 0.61 (0.37) | 0.118 |
|  | moderate active × men × pandemic | 0.66 (0.20-2.20) | -0.41 (0.61) | 0.507 |
|  | moderate active × pandemic | 0.94 (0.44-2.02) | -0.06 (0.39) | 0.882 |
|  | non-hypertension × men × pandemic | 1.12 (0.58-2.15) | 0.11 (0.33) | 0.739 |
|  | non-obese × men × pandemic | 0.79 (0.47-1.35) | -0.23 (0.27) | 0.402 |
|  | obesity × men × pandemic | 1.83 (1.04-3.24) | 0.61 (0.29) | 0.046 |
|  | obesity × pandemic | 0.70 (0.47-1.06) | -0.35 (0.21) | 0.105 |
|  | part-time employee × men | 1.07 (0.44-2.63) | 0.07 (0.46) | 0.882 |
|  | part-time employee × men × pandemic | 0.37 (0.07-1.84) | -1.01 (0.83) | 0.235 |
|  | part-time employee × pandemic | 2.10 (0.87-5.07) | 0.74 (0.45) | 0.114 |
|  | sleep hours (weekdays) <6h × men × pandemic | 1.72 (0.60-4.87) | 0.54 (0.53) | 0.321 |
|  | sleep hours (weekdays): 6-8h × men | 1.24 (0.58-2.64) | 0.21 (0.39) | 0.592 |
|  | sleep hours (weekdays): 6-8h × men × pandemic | 0.62 (0.19-2.06) | -0.48 (0.61) | 0.443 |
|  | sleep hours (weekdays): 6-8h × pandemic | 0.70 (0.29-1.66) | -0.36 (0.44) | 0.426 |
|  | sleep hours (weekdays)≥8h × men | 1.37 (0.66-2.85) | 0.31 (0.37) | 0.41 |
|  | sleep hours (weekdays)≥8h × men × pandemic | 0.66 (0.21-2.01) | -0.42 (0.57) | 0.469 |
|  | sleep hours (weekdays)≥8h × pandemic | 0.91 (0.36-2.32) | -0.09 (0.48) | 0.847 |
|  | sleep hours (weekends): 6-8h × men | 0.80 (0.48-1.35) | -0.22 (0.27) | 0.417 |
|  | sleep hours (weekends): 6-8h × men × pandemic | 1.47 (0.60-3.58) | 0.38 (0.46) | 0.409 |
|  | sleep hours (weekends): 6-8h × pandemic | 0.80 (0.44-1.44) | -0.23 (0.30) | 0.458 |
|  | sleep hours (weekends)≥8h × men | 0.93 (0.44-1.98) | -0.07 (0.38) | 0.86 |
|  | sleep hours (weekends)≥8h × men × pandemic | 0.78 (0.27-2.28) | -0.24 (0.55) | 0.657 |
|  | sleep hours (weekends)≥8h × pandemic | 0.95 (0.57-1.59) | -0.05 (0.26) | 0.857 |
|  | some college × men | 1.16 (0.69-1.95) | 0.15 (0.26) | 0.582 |
|  | some college × men × pandemic | 1.14 (0.62-2.13) | 0.13 (0.32) | 0.674 |
|  | some college × pandemic | 1.21 (0.74-1.98) | 0.19 (0.25) | 0.446 |
|  | US-born × men × pandemic | 1.06 (0.70-1.60) | 0.06 (0.21) | 0.794 |
|  | alcohol consumption × pandemic | 1.05 (0.68-1.61) | 0.05 (0.22) | 0.835 |
|  | born outside the US × pandemic | 0.79 (0.51-1.22) | -0.24 (0.22) | 0.295 |
|  | CDAI × pandemic | 1.07 (0.87-1.31) | 0.06 (0.11) | 0.542 |
|  | college graduate or above × pandemic | 0.84 (0.50-1.39) | -0.18 (0.26) | 0.493 |
|  | current smoker × pandemic | 1.76 (1.01-3.06) | 0.56 (0.28) | 0.055 |
|  | depression symptoms × pandemic | 1.04 (0.75-1.44) | 0.04 (0.17) | 0.827 |
|  | DII × pandemic | 0.93 (0.79-1.11) | -0.07 (0.09) | 0.436 |
|  | divorced, separated or widowed × pandemic | 0.74 (0.43-1.27) | -0.30 (0.28) | 0.285 |
|  | ex-smoker × pandemic | 1.03 (0.65-1.64) | 0.03 (0.23) | 0.886 |
|  | full-time employee × pandemic | 1.00 (0.74-1.35) | 0.00 (0.15) | 0.991 |
|  | health insurance × pandemic | 1.08 (0.54-2.14) | 0.08 (0.35) | 0.829 |
|  | high active × pandemic | 1.22 (0.70-2.13) | 0.20 (0.28) | 0.484 |
|  | high income × pandemic | 0.73 (0.48-1.13) | -0.31 (0.22) | 0.171 |
|  | hypertension × pandemic | 0.78 (0.57-1.06) | -0.25 (0.16) | 0.127 |
|  | married or living with partner × pandemic | 0.72 (0.45-1.18) | -0.32 (0.25) | 0.202 |
|  | middle income × pandemic | 1.19 (0.89-1.60) | 0.18 (0.15) | 0.255 |
|  | moderate active × pandemic | 0.74 (0.46-1.20) | -0.30 (0.24) | 0.232 |
|  | obesity × pandemic | 0.98 (0.73-1.33) | -0.02 (0.15) | 0.912 |
|  | part-time employee × pandemic | 1.38 (0.83-2.29) | 0.32 (0.26) | 0.218 |
|  | sleep hours (weekdays): 6-8h × pandemic | 0.53 (0.31-0.92) | -0.63 (0.28) | 0.03 |
|  | sleep hours (weekdays)≥8h × pandemic | 0.72 (0.38-1.36) | -0.32 (0.32) | 0.324 |
|  | sleep hours (weekends): 6-8h × pandemic | 1.00 (0.63-1.59) | -0.00 (0.24) | 0.987 |
|  | sleep hours (weekends)≥8h × pandemic | 0.85 (0.62-1.17) | -0.17 (0.16) | 0.316 |
|  | some college × pandemic | 1.30 (0.92-1.83) | 0.26 (0.17) | 0.143 |
|  | age × full-time employee | 1.03 (1.00-1.06) | 0.03 (0.02) | 0.052 |
|  | age × full-time employee × pandemic | 0.98 (0.94-1.03) | -0.02 (0.02) | 0.424 |
|  | age × not-employed × pandemic | 1.00 (0.99-1.02) | 0.00 (0.01) | 0.658 |
|  | age × part-time employee | 1.00 (0.97-1.03) | 0.00 (0.01) | 0.854 |
|  | age × part-time employee × pandemic | 0.98 (0.93-1.04) | -0.02 (0.03) | 0.523 |
|  | full-time employee × pandemic | 1.91 (0.17-21.00) | 0.65 (1.22) | 0.609 |
|  | men × age × full-time employee | 1.00 (0.96-1.04) | -0.00 (0.02) | 0.972 |
|  | men × age × full-time employee × pandemic | 1.03 (0.98-1.09) | 0.03 (0.03) | 0.278 |
|  | men × age × not-employed × pandemic | 0.98 (0.95-1.02) | -0.02 (0.02) | 0.319 |
|  | men × age × part-time employee | 1.00 (0.96-1.05) | 0.00 (0.02) | 0.909 |
|  | men × age × part-time employee × pandemic | 1.04 (0.97-1.11) | 0.04 (0.03) | 0.309 |
|  | men × full-time employee | 0.75 (0.07-7.83) | -0.29 (1.20) | 0.812 |
|  | men × full-time employee × pandemic | 0.25 (0.01-6.40) | -1.38 (1.65) | 0.422 |
|  | men × not-employed × age | 1.01 (0.99-1.04) | 0.01 (0.01) | 0.337 |
|  | men × not-employed × pandemic | 3.27 (0.27-40.16) | 1.19 (1.28) | 0.374 |
|  | men × part-time employee | 1.13 (0.08-15.96) | 0.12 (1.35) | 0.929 |
|  | men × part-time employee × pandemic | 0.03 (0.00-1.21) | -3.53 (1.90) | 0.09 |
|  | part-time employee × pandemic | 5.71 (0.26-127.59) | 1.74 (1.58) | 0.295 |
|  | age × alcohol consumption | 1.00 (0.97-1.02) | -0.00 (0.01) | 0.83 |
|  | age × alcohol consumption × pandemic | 0.99 (0.96-1.02) | -0.01 (0.02) | 0.338 |
|  | alcohol consumption × pandemic | 5.00 (1.00-24.99) | 1.61 (0.82) | 0.065 |
|  | men × age × alcohol consumption | 1.02 (0.99-1.06) | 0.02 (0.02) | 0.276 |
|  | men × age × alcohol consumption × pandemic | 0.99 (0.95-1.03) | -0.01 (0.02) | 0.615 |
|  | men × alcohol consumption | 0.76 (0.12-4.68) | -0.28 (0.93) | 0.767 |
|  | men × alcohol consumption × pandemic | 0.78 (0.10-6.08) | -0.25 (1.05) | 0.811 |
|  | age × born outside the US | 0.99 (0.98-1.01) | -0.01 (0.01) | 0.497 |
|  | age × born outside the US × pandemic | 1.01 (0.98-1.04) | 0.01 (0.02) | 0.481 |
|  | age × US-born × pandemic | 1.00 (0.98-1.01) | -0.00 (0.01) | 0.474 |
|  | born outside the US × pandemic | 0.33 (0.04-2.55) | -1.11 (1.04) | 0.301 |
|  | men × age × born outside the US | 0.99 (0.95-1.02) | -0.01 (0.02) | 0.464 |
|  | men × age × born outside the US × pandemic | 1.04 (0.99-1.09) | 0.04 (0.03) | 0.174 |
|  | men × born outside the US | 1.42 (0.17-11.87) | 0.35 (1.08) | 0.748 |
|  | men × born outside the US × pandemic | 0.18 (0.01-4.58) | -1.69 (1.64) | 0.315 |
|  | US-born × men × age | 1.02 (1.01-1.04) | 0.02 (0.01) | 0.013 |
|  | US-born × men × age × pandemic | 0.99 (0.96-1.01) | -0.01 (0.01) | 0.212 |
|  | US-born × men × pandemic | 2.24 (0.49-10.18) | 0.81 (0.77) | 0.309 |
|  | age × CDAI | 0.99 (0.98-1.00) | -0.01 (0.01) | 0.087 |
|  | age × CDAI × pandemic | 1.02 (1.00-1.03) | 0.02 (0.01) | 0.026 |
|  | CDAI × pandemic | 0.37 (0.15-0.91) | -0.98 (0.45) | 0.043 |
|  | men × age × CDAI | 1.01 (0.99-1.02) | 0.01 (0.01) | 0.327 |
|  | men × age × CDAI × pandemic | 0.98 (0.97-1.00) | -0.02 (0.01) | 0.105 |
|  | men × CDAI | 0.79 (0.40-1.56) | -0.23 (0.34) | 0.51 |
|  | men × CDAI × pandemic | 2.91 (0.95-8.88) | 1.07 (0.57) | 0.076 |
|  | age × depression symptoms | 1.00 (0.98-1.02) | 0.00 (0.01) | 0.773 |
|  | age × depression symptoms × pandemic | 0.99 (0.95-1.03) | -0.01 (0.02) | 0.65 |
|  | depression symptoms × pandemic | 1.61 (0.17-15.05) | 0.47 (1.14) | 0.682 |
|  | men × age × depression symptoms | 1.00 (0.96-1.05) | 0.00 (0.02) | 0.838 |
|  | men × age × depression symptoms × pandemic | 1.00 (0.94-1.07) | 0.00 (0.03) | 0.924 |
|  | men × age × pandemic | 0.99 (0.96-1.02) | -0.01 (0.02) | 0.405 |
|  | men × depression symptoms | 0.50 (0.04-6.01) | -0.69 (1.27) | 0.591 |
|  | men × depression symptoms × pandemic | 1.21 (0.02-60.62) | 0.19 (2.00) | 0.924 |
|  | age × DII | 1.01 (1.00-1.02) | 0.01 (0.00) | 0.011 |
|  | age × DII × pandemic | 0.98 (0.97-1.00) | -0.02 (0.01) | 0.068 |
|  | DII × pandemic | 2.72 (0.89-8.31) | 1.00 (0.57) | 0.095 |
|  | men × age × DII | 0.99 (0.98-1.00) | -0.01 (0.01) | 0.09 |
|  | men × age × DII × pandemic | 1.02 (0.99-1.04) | 0.02 (0.01) | 0.169 |
|  | men × DII | 1.84 (0.93-3.65) | 0.61 (0.35) | 0.094 |
|  | men × DII × pandemic | 0.30 (0.07-1.30) | -1.20 (0.74) | 0.124 |
|  | age × college graduate or above | 1.00 (0.97-1.02) | -0.00 (0.01) | 0.845 |
|  | age × college graduate or above × pandemic | 1.04 (0.98-1.09) | 0.04 (0.03) | 0.196 |
|  | age × less than college × pandemic | 0.99 (0.97-1.01) | -0.01 (0.01) | 0.453 |
|  | age × some college | 1.02 (0.99-1.04) | 0.02 (0.01) | 0.18 |
|  | age × some college × pandemic | 1.00 (0.96-1.03) | -0.00 (0.02) | 0.902 |
|  | college graduate or above × pandemic | 0.09 (0.01-1.35) | -2.43 (1.39) | 0.109 |
|  | less than college × men × age | 1.02 (1.00-1.05) | 0.02 (0.01) | 0.126 |
|  | men × age × college graduate or above | 1.00 (0.97-1.04) | 0.00 (0.02) | 0.822 |
|  | men × age × college graduate or above × pandemic | 0.95 (0.90-1.01) | -0.05 (0.03) | 0.116 |
|  | men × age × less than college × pandemic | 1.01 (0.97-1.05) | 0.01 (0.02) | 0.654 |
|  | men × age × some college | 1.00 (0.96-1.03) | -0.00 (0.02) | 0.801 |
|  | men × age × some college × pandemic | 0.98 (0.92-1.04) | -0.02 (0.03) | 0.525 |
|  | men × college graduate or above | 0.90 (0.10-8.05) | -0.11 (1.12) | 0.925 |
|  | men × college graduate or above × pandemic | 27.02 (0.93-786.29) | 3.30 (1.72) | 0.082 |
|  | men × less than college × pandemic | 0.47 (0.03-7.18) | -0.76 (1.39) | 0.597 |
|  | men × some college | 1.48 (0.17-12.61) | 0.39 (1.09) | 0.729 |
|  | men × some college × pandemic | 4.02 (0.11-151.04) | 1.39 (1.85) | 0.468 |
|  | some college × pandemic | 1.37 (0.14-12.89) | 0.31 (1.15) | 0.791 |
|  | age × health insurance | 1.00 (0.96-1.03) | -0.00 (0.02) | 0.836 |
|  | age × health insurance × pandemic | 1.03 (0.97-1.10) | 0.03 (0.03) | 0.327 |
|  | age × no health insurance × pandemic | 0.97 (0.91-1.02) | -0.03 (0.03) | 0.252 |
|  | health insurance × pandemic | 0.37 (0.02-6.38) | -1.00 (1.45) | 0.501 |
|  | men × age × health insurance | 0.98 (0.92-1.03) | -0.03 (0.03) | 0.412 |
|  | men × age × health insurance × pandemic | 0.96 (0.86-1.07) | -0.04 (0.06) | 0.489 |
|  | men × age × no health insurance × pandemic | 1.03 (0.93-1.15) | 0.03 (0.05) | 0.561 |
|  | men × health insurance | 9.34 (0.52-168.13) | 2.23 (1.47) | 0.146 |
|  | men × health insurance × pandemic | 3.95 (0.02-879.93) | 1.37 (2.76) | 0.624 |
|  | men × no health insurance × age | 1.04 (0.98-1.10) | 0.04 (0.03) | 0.184 |
|  | men × no health insurance × pandemic | 0.40 (0.00-71.73) | -0.92 (2.65) | 0.732 |
|  | age × divorced, separated or widowed | 0.99 (0.95-1.03) | -0.01 (0.02) | 0.609 |
|  | age × divorced, separated or widowed × pandemic | 0.99 (0.95-1.04) | -0.01 (0.02) | 0.702 |
|  | age × married or living with partner | 0.99 (0.96-1.02) | -0.01 (0.01) | 0.392 |
|  | age × married or living with partner × pandemic | 1.01 (0.97-1.06) | 0.01 (0.02) | 0.648 |
|  | age × never married × pandemic | 1.00 (0.96-1.03) | -0.00 (0.02) | 0.872 |
|  | divorced, separated or widowed × pandemic | 1.06 (0.08-14.85) | 0.06 (1.35) | 0.965 |
|  | men × age × divorced, separated or widowed | 1.02 (0.97-1.06) | 0.02 (0.02) | 0.464 |
|  | men × age × divorced, separated or widowed × pandemic | 0.97 (0.89-1.06) | -0.03 (0.04) | 0.524 |
|  | men × age × married or living with partner | 1.02 (0.98-1.07) | 0.02 (0.02) | 0.332 |
|  | men × age × married or living with partner × pandemic | 0.95 (0.89-1.02) | -0.05 (0.03) | 0.204 |
|  | men × age × never married × pandemic | 1.02 (0.97-1.07) | 0.02 (0.03) | 0.454 |
|  | men × divorced, separated or widowed | 0.27 (0.02-3.27) | -1.30 (1.27) | 0.327 |
|  | men × divorced, separated or widowed × pandemic | 11.76 (0.09-1604.18) | 2.47 (2.51) | 0.347 |
|  | men × married or living with partner | 0.23 (0.03-1.99) | -1.45 (1.09) | 0.211 |
|  | men × married or living with partner × pandemic | 33.82 (0.97-1184.52) | 3.52 (1.81) | 0.078 |
|  | men × never married × age | 1.00 (0.97-1.04) | 0.00 (0.02) | 0.843 |
|  | men × never married × pandemic | 0.16 (0.01-1.98) | -1.84 (1.29) | 0.181 |
|  | married or living with partner × pandemic | 0.30 (0.03-3.13) | -1.21 (1.20) | 0.335 |
|  | age × high active | 0.98 (0.95-1.01) | -0.02 (0.01) | 0.152 |
|  | age × high active × pandemic | 1.01 (0.97-1.05) | 0.01 (0.02) | 0.659 |
|  | age × low active × pandemic | 1.00 (0.98-1.02) | -0.00 (0.01) | 0.995 |
|  | age × moderate active | 1.02 (0.98-1.06) | 0.02 (0.02) | 0.304 |
|  | age × moderate active × pandemic | 0.99 (0.94-1.04) | -0.01 (0.03) | 0.706 |
|  | high active × pandemic | 1.01 (0.08-12.28) | 0.01 (1.27) | 0.991 |
|  | low active × men × age | 1.02 (1.00-1.04) | 0.02 (0.01) | 0.106 |
|  | low active × men × age × pandemic | 0.99 (0.96-1.02) | -0.01 (0.01) | 0.369 |
|  | low active × men × pandemic | 2.78 (0.38-20.54) | 1.02 (1.02) | 0.337 |
|  | men × age × high active | 1.03 (1.00-1.07) | 0.03 (0.02) | 0.114 |
|  | men × age × high active × pandemic | 0.99 (0.93-1.04) | -0.01 (0.03) | 0.612 |
|  | men × age × moderate active | 0.97 (0.92-1.02) | -0.03 (0.03) | 0.244 |
|  | men × age × moderate active × pandemic | 1.03 (0.96-1.10) | 0.03 (0.03) | 0.442 |
|  | men × high active | 0.24 (0.03-1.88) | -1.43 (1.05) | 0.201 |
|  | men × high active × pandemic | 1.20 (0.05-29.62) | 0.18 (1.64) | 0.915 |
|  | men × moderate active | 11.80 (0.39-356.92) | 2.47 (1.74) | 0.184 |
|  | men × moderate active × pandemic | 0.13 (0.00-8.44) | -2.07 (2.15) | 0.355 |
|  | moderate active × pandemic | 1.64 (0.08-35.79) | 0.49 (1.57) | 0.759 |
|  | age × high income | 1.00 (0.97-1.02) | -0.00 (0.01) | 0.738 |
|  | age × high income × pandemic | 1.02 (0.97-1.06) | 0.02 (0.02) | 0.452 |
|  | age × low income × pandemic | 1.00 (0.97-1.02) | -0.00 (0.01) | 0.776 |
|  | age × middle income | 1.00 (0.97-1.02) | -0.00 (0.01) | 0.676 |
|  | age × middle income × pandemic | 1.00 (0.96-1.03) | -0.00 (0.02) | 0.891 |
|  | high income × pandemic | 0.23 (0.02-2.41) | -1.46 (1.19) | 0.247 |
|  | low income × men × age | 1.01 (0.98-1.04) | 0.01 (0.02) | 0.444 |
|  | low income × men × pandemic | 0.75 (0.03-20.90) | -0.29 (1.70) | 0.866 |
|  | men × age × high income | 1.00 (0.97-1.03) | 0.00 (0.02) | 0.861 |
|  | men × age × high income × pandemic | 0.98 (0.92-1.04) | -0.02 (0.03) | 0.457 |
|  | men × age × low income × pandemic | 1.00 (0.96-1.06) | 0.00 (0.03) | 0.871 |
|  | men × age × middle income | 1.02 (0.98-1.06) | 0.02 (0.02) | 0.41 |
|  | men × age × middle income × pandemic | 0.98 (0.92-1.04) | -0.02 (0.03) | 0.507 |
|  | men × high income | 1.00 (0.15-6.68) | -0.00 (0.97) | 0.998 |
|  | men × high income × pandemic | 4.80 (0.13-180.21) | 1.57 (1.85) | 0.415 |
|  | men × middle income | 0.45 (0.04-4.50) | -0.81 (1.18) | 0.507 |
|  | men × middle income × pandemic | 3.86 (0.08-181.13) | 1.35 (1.96) | 0.506 |
|  | middle income × pandemic | 1.31 (0.16-10.92) | 0.27 (1.08) | 0.807 |
|  | age × sleep hours (weekdays) <6h × pandemic | 0.98 (0.95-1.01) | -0.02 (0.02) | 0.323 |
|  | age × sleep hours (weekdays): 6-8h | 1.02 (1.00-1.05) | 0.02 (0.01) | 0.128 |
|  | age × sleep hours (weekdays): 6-8h × pandemic | 1.00 (0.97-1.04) | 0.00 (0.02) | 0.878 |
|  | age × sleep hours (weekdays)≥8h | 1.01 (0.99-1.04) | 0.01 (0.01) | 0.279 |
|  | age × sleep hours (weekdays)≥8h × pandemic | 1.02 (0.98-1.06) | 0.02 (0.02) | 0.282 |
|  | men × age × sleep hours (weekdays): 6-8h | 0.99 (0.96-1.02) | -0.01 (0.02) | 0.401 |
|  | men × age × sleep hours (weekdays): 6-8h × pandemic | 1.03 (0.96-1.11) | 0.03 (0.04) | 0.376 |
|  | men × age × sleep hours (weekdays)≥8h | 1.00 (0.97-1.04) | 0.00 (0.02) | 0.837 |
|  | men × age × sleep hours (weekdays)≥8h × pandemic | 0.98 (0.92-1.05) | -0.02 (0.04) | 0.611 |
|  | men × sleep hours (weekdays): 6-8h | 2.86 (0.38-21.33) | 1.05 (1.02) | 0.327 |
|  | men × sleep hours (weekdays): 6-8h × pandemic | 0.10 (0.00-5.11) | -2.34 (2.03) | 0.272 |
|  | men × sleep hours (weekdays)≥8h | 0.97 (0.11-8.59) | -0.03 (1.11) | 0.979 |
|  | men × sleep hours (weekdays)≥8h × pandemic | 2.21 (0.04-121.50) | 0.79 (2.05) | 0.706 |
|  | sleep hours (weekdays) <6h × men × age | 1.02 (1.00-1.05) | 0.02 (0.01) | 0.124 |
|  | sleep hours (weekdays) <6h × men × age × pandemic | 0.99 (0.93-1.05) | -0.01 (0.03) | 0.682 |
|  | sleep hours (weekdays) <6h × men × pandemic | 3.30 (0.09-120.65) | 1.19 (1.84) | 0.529 |
|  | sleep hours (weekdays): 6-8h × pandemic | 0.61 (0.08-4.54) | -0.50 (1.03) | 0.636 |
|  | sleep hours (weekdays)≥8h × pandemic | 0.27 (0.03-2.20) | -1.31 (1.07) | 0.246 |
|  | age × sleep hours (weekdays) <6h × pandemic | 1.00 (0.97-1.02) | -0.00 (0.01) | 0.706 |
|  | age × sleep hours (weekends): 6-8h | 1.01 (0.99-1.03) | 0.01 (0.01) | 0.468 |
|  | age × sleep hours (weekends): 6-8h × pandemic | 0.99 (0.96-1.03) | -0.01 (0.02) | 0.731 |
|  | age × sleep hours (weekends)≥8h | 1.01 (0.99-1.03) | 0.01 (0.01) | 0.514 |
|  | age × sleep hours (weekends)≥8h × pandemic | 1.01 (0.98-1.04) | 0.01 (0.02) | 0.558 |
|  | men × age × sleep hours (weekends): 6-8h | 0.98 (0.94-1.01) | -0.02 (0.02) | 0.175 |
|  | men × age × sleep hours (weekends): 6-8h × pandemic | 1.00 (0.95-1.06) | 0.00 (0.03) | 0.926 |
|  | men × age × sleep hours (weekends)≥8h | 0.99 (0.96-1.02) | -0.01 (0.02) | 0.461 |
|  | men × age × sleep hours (weekends)≥8h × pandemic | 1.01 (0.96-1.05) | 0.01 (0.02) | 0.835 |
|  | men × sleep hours (weekdays) <6h × pandemic | 2.06 (0.11-36.92) | 0.72 (1.47) | 0.634 |
|  | men × sleep hours (weekends): 6-8h | 3.31 (0.40-27.52) | 1.20 (1.08) | 0.292 |
|  | men × sleep hours (weekends): 6-8h × pandemic | 1.35 (0.03-59.75) | 0.30 (1.94) | 0.881 |
|  | men × sleep hours (weekends)≥8h | 1.88 (0.25-14.09) | 0.63 (1.03) | 0.552 |
|  | men × sleep hours (weekends)≥8h × pandemic | 0.58 (0.03-12.98) | -0.54 (1.58) | 0.74 |
|  | sleep hours (weekdays) <6h × men × age | 1.03 (1.01-1.06) | 0.03 (0.01) | 0.037 |
|  | sleep hours (weekdays) <6h × men × age × pandemic | 0.99 (0.95-1.03) | -0.01 (0.02) | 0.582 |
|  | sleep hours (weekends): 6-8h × pandemic | 1.22 (0.12-12.88) | 0.20 (1.20) | 0.872 |
|  | sleep hours (weekends)≥8h × pandemic | 0.57 (0.09-3.67) | -0.55 (0.95) | 0.57 |
|  | age × current smoker | 0.98 (0.96-1.00) | -0.02 (0.01) | 0.06 |
|  | age × current smoker × pandemic | 1.00 (0.97-1.03) | -0.00 (0.02) | 0.897 |
|  | age × ex-smoker | 0.98 (0.96-0.99) | -0.02 (0.01) | 0.016 |
|  | age × ex-smoker × pandemic | 1.01 (0.99-1.04) | 0.01 (0.01) | 0.362 |
|  | age × never-smoker × pandemic | 1.00 (0.98-1.01) | -0.00 (0.01) | 0.494 |
|  | current smoker × pandemic | 1.88 (0.27-13.21) | 0.63 (0.99) | 0.538 |
|  | ex-smoker × pandemic | 0.50 (0.09-2.69) | -0.69 (0.86) | 0.437 |
|  | men × age × current smoker | 1.06 (1.02-1.10) | 0.06 (0.02) | 0.02 |
|  | men × age × current smoker × pandemic | 0.97 (0.91-1.03) | -0.03 (0.03) | 0.304 |
|  | men × age × ex-smoker | 1.01 (0.98-1.04) | 0.01 (0.01) | 0.456 |
|  | men × age × ex-smoker × pandemic | 0.98 (0.93-1.02) | -0.02 (0.02) | 0.297 |
|  | men × age × never-smoker × pandemic | 1.00 (0.97-1.03) | 0.00 (0.01) | 0.977 |
|  | men × current smoker | 0.03 (0.00-0.34) | -3.46 (1.22) | 0.016 |
|  | men × current smoker × pandemic | 5.89 (0.16-217.10) | 1.77 (1.84) | 0.356 |
|  | men × ex-smoker | 0.61 (0.09-4.02) | -0.49 (0.96) | 0.617 |
|  | men × ex-smoker × pandemic | 4.13 (0.23-74.29) | 1.42 (1.47) | 0.357 |
|  | men × never-smoker × age | 1.01 (0.99-1.03) | 0.01 (0.01) | 0.292 |
|  | men × never-smoker × pandemic | 1.02 (0.15-7.10) | 0.02 (0.99) | 0.982 |
|  | men × ≥60 years | 2.52 (1.08-5.88) | 0.92 (0.43) | 0.044 |
|  | men × ≥60 years × pandemic | 0.56 (0.19-1.69) | -0.58 (0.56) | 0.313 |
|  | men × 40-59 years | 1.92 (0.81-4.54) | 0.65 (0.44) | 0.151 |
|  | men × 40-59 years × pandemic | 0.67 (0.18-2.51) | -0.40 (0.67) | 0.558 |
|  | men × pandemic | 1.66 (0.60-4.58) | 0.50 (0.52) | 0.342 |
|  | sex × ≥60 years × pandemic | 1.18 (0.62-2.26) | 0.17 (0.33) | 0.616 |
|  | sex × 40-59 years × pandemic | 1.28 (0.56-2.93) | 0.25 (0.42) | 0.565 |
|  | DII × pandemic | 0.82 (0.47-1.42) | -0.20 (0.28) | 0.483 |
|  | DII × sleep hours (weekdays): 6-8h | 0.80 (0.61-1.07) | -0.22 (0.14) | 0.143 |
|  | DII × sleep hours (weekdays): 6-8h × pandemic | 1.11 (0.65-1.92) | 0.11 (0.28) | 0.705 |
|  | DII × sleep hours (weekdays)≥8h | 0.80 (0.56-1.13) | -0.23 (0.18) | 0.213 |
|  | DII × sleep hours (weekdays)≥8h × pandemic | 1.17 (0.65-2.09) | 0.16 (0.30) | 0.606 |
|  | DII × pandemic | 1.33 (0.97-1.83) | 0.28 (0.16) | 0.094 |
|  | DII × sleep hours (weekends): 6-8h | 1.14 (0.80-1.63) | 0.13 (0.18) | 0.481 |
|  | DII × sleep hours (weekends): 6-8h × pandemic | 0.57 (0.34-0.98) | -0.55 (0.27) | 0.053 |
|  | DII × sleep hours (weekends)≥8h | 1.32 (1.03-1.69) | 0.28 (0.13) | 0.036 |
|  | DII × sleep hours (weekends)≥8h × pandemic | 0.68 (0.47-0.98) | -0.39 (0.19) | 0.051 |
|  | hypertension × full-time employee | 1.05 (0.64-1.72) | 0.05 (0.25) | 0.84 |
|  | hypertension × full-time employee × pandemic | 0.82 (0.45-1.49) | -0.20 (0.31) | 0.511 |
|  | hypertension × pandemic | 0.82 (0.53-1.25) | -0.20 (0.22) | 0.358 |
|  | hypertension × part-time employee | 0.82 (0.34-1.98) | -0.20 (0.45) | 0.658 |
|  | hypertension × part-time employee × pandemic | 1.09 (0.29-4.12) | 0.08 (0.68) | 0.901 |
|  | non-hypertension × full-time employee × pandemic | 1.10 (0.72-1.66) | 0.09 (0.21) | 0.668 |
|  | non-hypertension × part-time employee × pandemic | 1.27 (0.49-3.27) | 0.24 (0.48) | 0.63 |
|  | hypertension × alcohol consumption | 1.51 (0.65-3.51) | 0.41 (0.43) | 0.348 |
|  | hypertension × alcohol consumption × pandemic | 0.61 (0.23-1.63) | -0.49 (0.50) | 0.334 |
|  | hypertension × pandemic | 0.57 (0.38-0.86) | -0.56 (0.21) | 0.013 |
|  | non-hypertension × alcohol consumption × pandemic | 1.58 (0.57-4.35) | 0.46 (0.52) | 0.386 |
|  | hypertension × born outside the US | 0.50 (0.34-0.72) | -0.70 (0.19) | 0.001 |
|  | hypertension × born outside the US × pandemic | 3.34 (1.76-6.32) | 1.20 (0.33) | 0.001 |
|  | hypertension × pandemic | 0.62 (0.44-0.87) | -0.48 (0.18) | 0.011 |
|  | non-hypertension × born outside the US × pandemic | 0.38 (0.23-0.63) | -0.96 (0.25) | 0.001 |
|  | hypertension × CDAI | 0.80 (0.63-1.02) | -0.22 (0.12) | 0.086 |
|  | hypertension × CDAI × pandemic | 1.04 (0.76-1.41) | 0.04 (0.16) | 0.806 |
|  | hypertension × pandemic | 0.77 (0.57-1.04) | -0.26 (0.15) | 0.098 |
|  | non-hypertension × CDAI × pandemic | 1.02 (0.78-1.34) | 0.02 (0.14) | 0.885 |
|  | hypertension × depression symptoms | 1.30 (0.80-2.12) | 0.26 (0.25) | 0.297 |
|  | hypertension × depression symptoms × pandemic | 0.88 (0.48-1.61) | -0.13 (0.31) | 0.683 |
|  | hypertension × pandemic | 0.80 (0.57-1.12) | -0.22 (0.17) | 0.203 |
|  | non-hypertension × depression symptoms × pandemic | 1.15 (0.76-1.74) | 0.14 (0.21) | 0.512 |
|  | hypertension × DII | 1.19 (0.91-1.57) | 0.18 (0.14) | 0.215 |
|  | hypertension × DII × pandemic | 0.94 (0.68-1.32) | -0.06 (0.17) | 0.738 |
|  | hypertension × pandemic | 0.78 (0.58-1.06) | -0.25 (0.16) | 0.119 |
|  | non-hypertension × DII × pandemic | 0.98 (0.75-1.28) | -0.02 (0.14) | 0.884 |
|  | hypertension × college graduate or above | 1.26 (0.61-2.63) | 0.23 (0.37) | 0.536 |
|  | hypertension × college graduate or above × pandemic | 0.63 (0.20-1.99) | -0.46 (0.59) | 0.439 |
|  | hypertension × pandemic | 0.84 (0.44-1.58) | -0.18 (0.32) | 0.587 |
|  | hypertension × some college | 0.97 (0.58-1.63) | -0.03 (0.26) | 0.922 |
|  | hypertension × some college × pandemic | 1.10 (0.43-2.83) | 0.10 (0.48) | 0.844 |
|  | non-hypertension × college graduate or above × pandemic | 1.08 (0.40-2.92) | 0.08 (0.51) | 0.883 |
|  | non-hypertension × some college × pandemic | 1.22 (0.54-2.75) | 0.20 (0.42) | 0.641 |
|  | hypertension × health insurance | 0.86 (0.46-1.61) | -0.15 (0.32) | 0.641 |
|  | hypertension × health insurance × pandemic | 1.38 (0.40-4.77) | 0.32 (0.63) | 0.614 |
|  | hypertension × pandemic | 0.58 (0.20-1.71) | -0.54 (0.55) | 0.334 |
|  | non-hypertension × health insurance × pandemic | 0.92 (0.31-2.72) | -0.08 (0.55) | 0.888 |
|  | hypertension × divorced, separated or widowed | 0.34 (0.15-0.78) | -1.09 (0.43) | 0.017 |
|  | hypertension × divorced, separated or widowed × pandemic | 0.49 (0.14-1.64) | -0.72 (0.62) | 0.257 |
|  | hypertension × married or living with partner | 0.57 (0.28-1.16) | -0.57 (0.36) | 0.131 |
|  | hypertension × married or living with partner × pandemic | 0.38 (0.13-1.10) | -0.98 (0.55) | 0.087 |
|  | hypertension × pandemic | 1.83 (0.67-5.00) | 0.60 (0.51) | 0.252 |
|  | non-hypertension × divorced, separated or widowed × pandemic | 1.26 (0.42-3.75) | 0.23 (0.56) | 0.684 |
|  | non-hypertension × married or living with partner × pandemic | 1.42 (0.52-3.87) | 0.35 (0.51) | 0.498 |
|  | hypertension × high active | 0.88 (0.45-1.72) | -0.12 (0.34) | 0.722 |
|  | hypertension × high active × pandemic | 1.59 (0.55-4.54) | 0.46 (0.54) | 0.398 |
|  | hypertension × moderate active | 2.21 (1.02-4.81) | 0.79 (0.40) | 0.057 |
|  | hypertension × moderate active × pandemic | 0.52 (0.17-1.57) | -0.65 (0.56) | 0.257 |
|  | hypertension × pandemic | 0.76 (0.46-1.26) | -0.28 (0.26) | 0.295 |
|  | non-hypertension × high active × pandemic | 0.92 (0.35-2.43) | -0.09 (0.50) | 0.864 |
|  | non-hypertension × moderate active × pandemic | 1.18 (0.50-2.78) | 0.16 (0.44) | 0.71 |
|  | hypertension × high income | 1.00 (0.57-1.75) | 0.00 (0.29) | 0.991 |
|  | hypertension × high income × pandemic | 0.54 (0.21-1.37) | -0.61 (0.47) | 0.209 |
|  | hypertension × middle income | 1.05 (0.52-2.12) | 0.05 (0.36) | 0.885 |
|  | hypertension × middle income × pandemic | 0.51 (0.18-1.47) | -0.68 (0.54) | 0.222 |
|  | hypertension × pandemic | 1.33 (0.58-3.05) | 0.29 (0.42) | 0.502 |
|  | non-hypertension × high income × pandemic | 1.10 (0.48-2.50) | 0.09 (0.42) | 0.826 |
|  | non-hypertension × middle income × pandemic | 1.87 (0.91-3.83) | 0.62 (0.37) | 0.102 |
|  | hypertension × pandemic | 1.38 (0.47-4.08) | 0.33 (0.55) | 0.56 |
|  | hypertension × sleep hours (weekdays): 6-8h | 1.61 (0.54-4.79) | 0.48 (0.56) | 0.397 |
|  | hypertension × sleep hours (weekdays): 6-8h × pandemic | 0.68 (0.19-2.47) | -0.38 (0.66) | 0.566 |
|  | hypertension × sleep hours (weekdays)≥8h | 1.68 (0.74-3.82) | 0.52 (0.42) | 0.231 |
|  | hypertension × sleep hours (weekdays)≥8h × pandemic | 0.43 (0.12-1.51) | -0.84 (0.64) | 0.201 |
|  | non-hypertension × sleep hours (weekdays): 6-8h × pandemic | 0.69 (0.28-1.67) | -0.37 (0.45) | 0.418 |
|  | non-hypertension × sleep hours (weekdays)≥8h × pandemic | 1.25 (0.55-2.84) | 0.23 (0.42) | 0.594 |
|  | hypertension × pandemic | 0.84 (0.40-1.76) | -0.18 (0.38) | 0.641 |
|  | hypertension × sleep hours (weekends): 6-8h | 0.66 (0.36-1.21) | -0.41 (0.31) | 0.195 |
|  | hypertension × sleep hours (weekends): 6-8h × pandemic | 1.13 (0.41-3.13) | 0.12 (0.52) | 0.816 |
|  | hypertension × sleep hours (weekends)≥8h | 0.98 (0.47-2.04) | -0.02 (0.37) | 0.967 |
|  | hypertension × sleep hours (weekends)≥8h × pandemic | 0.81 (0.34-1.93) | -0.20 (0.44) | 0.645 |
|  | non-hypertension × sleep hours (weekends): 6-8h × pandemic | 0.91 (0.35-2.34) | -0.09 (0.48) | 0.846 |
|  | non-hypertension × sleep hours (weekends)≥8h × pandemic | 0.96 (0.55-1.67) | -0.04 (0.28) | 0.884 |
|  | hypertension × current smoker | 0.59 (0.30-1.18) | -0.52 (0.35) | 0.152 |
|  | hypertension × current smoker × pandemic | 1.37 (0.52-3.59) | 0.31 (0.49) | 0.532 |
|  | hypertension × ex-smoker | 1.18 (0.68-2.04) | 0.17 (0.28) | 0.558 |
|  | hypertension × ex-smoker × pandemic | 0.78 (0.28-2.18) | -0.25 (0.52) | 0.642 |
|  | hypertension × pandemic | 0.81 (0.46-1.41) | -0.21 (0.29) | 0.459 |
|  | non-hypertension × current smoker × pandemic | 1.46 (0.69-3.08) | 0.38 (0.38) | 0.336 |
|  | non-hypertension × ex-smoker × pandemic | 1.26 (0.57-2.74) | 0.23 (0.40) | 0.573 |
|  | non-obese × full-time employee × pandemic | 1.01 (0.54-1.87) | 0.01 (0.32) | 0.976 |
|  | non-obese × part-time employee × pandemic | 1.29 (0.59-2.82) | 0.25 (0.40) | 0.536 |
|  | obesity × full-time employee | 1.09 (0.64-1.86) | 0.08 (0.27) | 0.761 |
|  | obesity × full-time employee × pandemic | 0.97 (0.41-2.28) | -0.03 (0.44) | 0.938 |
|  | obesity × pandemic | 1.02 (0.66-1.57) | 0.02 (0.22) | 0.932 |
|  | obesity × part-time employee | 1.74 (0.71-4.23) | 0.55 (0.45) | 0.235 |
|  | obesity × part-time employee × pandemic | 1.12 (0.30-4.15) | 0.12 (0.67) | 0.861 |
|  | non-obese × alcohol consumption × pandemic | 0.96 (0.49-1.85) | -0.04 (0.34) | 0.898 |
|  | obesity × alcohol consumption | 0.92 (0.54-1.57) | -0.08 (0.27) | 0.763 |
|  | obesity × alcohol consumption × pandemic | 1.14 (0.52-2.50) | 0.13 (0.40) | 0.751 |
|  | obesity × pandemic | 0.98 (0.67-1.44) | -0.02 (0.20) | 0.923 |
|  | non-obese × born outside the US × pandemic | 0.92 (0.55-1.53) | -0.09 (0.26) | 0.739 |
|  | obesity × born outside the US | 0.76 (0.47-1.23) | -0.28 (0.25) | 0.269 |
|  | obesity × born outside the US × pandemic | 0.68 (0.29-1.59) | -0.38 (0.43) | 0.384 |
|  | obesity × pandemic | 1.01 (0.70-1.46) | 0.01 (0.19) | 0.94 |
|  | non-obese × CDAI × pandemic | 1.05 (0.78-1.41) | 0.05 (0.15) | 0.768 |
|  | obesity × CDAI | 1.13 (0.90-1.42) | 0.13 (0.12) | 0.291 |
|  | obesity × CDAI × pandemic | 1.04 (0.75-1.46) | 0.04 (0.17) | 0.805 |
|  | obesity × pandemic | 1.00 (0.74-1.35) | -0.00 (0.15) | 0.997 |
|  | non-obese × depression symptoms × pandemic | 0.82 (0.45-1.50) | -0.20 (0.31) | 0.526 |
|  | obesity × depression symptoms | 0.95 (0.50-1.82) | -0.05 (0.33) | 0.889 |
|  | obesity × depression symptoms × pandemic | 1.40 (0.57-3.48) | 0.34 (0.46) | 0.47 |
|  | obesity × pandemic | 0.89 (0.61-1.29) | -0.12 (0.19) | 0.541 |
|  | non-obese × DII × pandemic | 1.04 (0.84-1.30) | 0.04 (0.11) | 0.708 |
|  | obesity × DII | 1.01 (0.83-1.24) | 0.01 (0.10) | 0.909 |
|  | obesity × DII × pandemic | 0.82 (0.62-1.09) | -0.20 (0.14) | 0.187 |
|  | obesity × pandemic | 1.01 (0.75-1.36) | 0.01 (0.15) | 0.933 |
|  | non-obese × college graduate or above × pandemic | 0.58 (0.27-1.21) | -0.55 (0.38) | 0.158 |
|  | non-obese × some college × pandemic | 1.16 (0.75-1.77) | 0.15 (0.22) | 0.511 |
|  | obesity × college graduate or above | 0.62 (0.34-1.14) | -0.48 (0.31) | 0.138 |
|  | obesity × college graduate or above × pandemic | 1.84 (0.71-4.77) | 0.61 (0.49) | 0.219 |
|  | obesity × pandemic | 0.75 (0.44-1.30) | -0.28 (0.28) | 0.322 |
|  | obesity × some college | 0.89 (0.57-1.39) | -0.12 (0.23) | 0.605 |
|  | obesity × some college × pandemic | 1.16 (0.56-2.38) | 0.15 (0.37) | 0.695 |
|  | non-obese × health insurance × pandemic | 0.97 (0.31-3.05) | -0.03 (0.59) | 0.955 |
|  | obesity × health insurance | 0.62 (0.26-1.47) | -0.47 (0.44) | 0.29 |
|  | obesity × health insurance × pandemic | 1.27 (0.32-5.09) | 0.24 (0.71) | 0.739 |
|  | obesity × pandemic | 0.80 (0.21-3.13) | -0.22 (0.70) | 0.753 |
|  | non-obese × divorced, separated or widowed × pandemic | 1.04 (0.40-2.74) | 0.04 (0.49) | 0.936 |
|  | non-obese × married or living with partner × pandemic | 0.68 (0.27-1.71) | -0.39 (0.47) | 0.418 |
|  | obesity × divorced, separated or widowed | 0.96 (0.39-2.37) | -0.04 (0.46) | 0.928 |
|  | obesity × divorced, separated or widowed × pandemic | 0.54 (0.15-1.99) | -0.62 (0.66) | 0.364 |
|  | obesity × married or living with partner | 0.89 (0.45-1.75) | -0.11 (0.34) | 0.743 |
|  | obesity × married or living with partner × pandemic | 1.06 (0.33-3.41) | 0.06 (0.60) | 0.92 |
|  | obesity × pandemic | 1.07 (0.37-3.10) | 0.06 (0.54) | 0.907 |
|  | non-obese × high active × pandemic | 1.84 (0.89-3.81) | 0.61 (0.37) | 0.116 |
|  | non-obese × moderate active × pandemic | 0.84 (0.41-1.71) | -0.18 (0.37) | 0.629 |
|  | obesity × high active | 2.68 (1.43-5.04) | 0.99 (0.32) | 0.005 |
|  | obesity × high active × pandemic | 0.52 (0.22-1.24) | -0.66 (0.44) | 0.153 |
|  | obesity × moderate active | 1.17 (0.49-2.78) | 0.15 (0.44) | 0.73 |
|  | obesity × moderate active × pandemic | 0.82 (0.27-2.47) | -0.20 (0.57) | 0.723 |
|  | obesity × pandemic | 1.08 (0.70-1.67) | 0.08 (0.22) | 0.719 |
|  | non-obese × high income × pandemic | 1.09 (0.60-1.98) | 0.09 (0.30) | 0.781 |
|  | non-obese × middle income × pandemic | 1.31 (0.72-2.38) | 0.27 (0.31) | 0.391 |
|  | obesity × high income | 1.45 (0.94-2.25) | 0.37 (0.22) | 0.108 |
|  | obesity × high income × pandemic | 0.52 (0.23-1.17) | -0.65 (0.41) | 0.129 |
|  | obesity × middle income | 1.21 (0.80-1.81) | 0.19 (0.21) | 0.374 |
|  | obesity × middle income × pandemic | 0.86 (0.39-1.93) | -0.15 (0.41) | 0.725 |
|  | obesity × pandemic | 1.35 (0.73-2.49) | 0.30 (0.31) | 0.353 |
|  | non-obese × sleep hours (weekdays): 6-8h × pandemic | 0.88 (0.34-2.23) | -0.13 (0.48) | 0.782 |
|  | non-obese × sleep hours (weekdays)≥8h × pandemic | 1.02 (0.36-2.86) | 0.02 (0.53) | 0.968 |
|  | obesity × pandemic | 1.63 (0.50-5.37) | 0.49 (0.61) | 0.427 |
|  | obesity × sleep hours (weekdays): 6-8h | 1.37 (0.68-2.76) | 0.32 (0.36) | 0.383 |
|  | obesity × sleep hours (weekdays): 6-8h × pandemic | 0.52 (0.14-1.88) | -0.66 (0.66) | 0.327 |
|  | obesity × sleep hours (weekdays)≥8h | 0.98 (0.44-2.18) | -0.02 (0.41) | 0.971 |
|  | obesity × sleep hours (weekdays)≥8h × pandemic | 0.67 (0.17-2.65) | -0.40 (0.70) | 0.575 |
|  | non-obese × sleep hours (weekends): 6-8h × pandemic | 0.85 (0.46-1.54) | -0.17 (0.31) | 0.59 |
|  | non-obese × sleep hours (weekends)≥8h × pandemic | 0.80 (0.44-1.44) | -0.22 (0.30) | 0.462 |
|  | obesity × pandemic | 0.90 (0.51-1.57) | -0.11 (0.29) | 0.707 |
|  | obesity × sleep hours (weekends): 6-8h | 1.19 (0.73-1.95) | 0.17 (0.25) | 0.497 |
|  | obesity × sleep hours (weekends): 6-8h × pandemic | 1.24 (0.56-2.73) | 0.21 (0.40) | 0.601 |
|  | obesity × sleep hours (weekends)≥8h | 0.75 (0.48-1.17) | -0.29 (0.23) | 0.218 |
|  | obesity × sleep hours (weekends)≥8h × pandemic | 1.06 (0.50-2.27) | 0.06 (0.39) | 0.881 |
|  | non-obese × current smoker × pandemic | 2.11 (0.84-5.32) | 0.75 (0.47) | 0.127 |
|  | non-obese × ex-smoker × pandemic | 1.58 (0.82-3.03) | 0.46 (0.33) | 0.183 |
|  | obesity × current smoker | 0.89 (0.44-1.83) | -0.11 (0.37) | 0.759 |
|  | obesity × current smoker × pandemic | 0.71 (0.23-2.20) | -0.35 (0.58) | 0.554 |
|  | obesity × ex-smoker | 1.27 (0.80-2.01) | 0.24 (0.24) | 0.329 |
|  | obesity × ex-smoker × pandemic | 0.50 (0.23-1.12) | -0.68 (0.41) | 0.108 |
|  | obesity × pandemic | 1.29 (0.84-1.98) | 0.26 (0.22) | 0.252 |
|  | depression symptoms × sleep hours (weekdays) <6h × pandemic | 0.94 (0.29-3.05) | -0.06 (0.60) | 0.923 |
|  | sleep hours (weekdays): 6-8h × depression symptoms | 0.63 (0.26-1.51) | -0.46 (0.45) | 0.313 |
|  | sleep hours (weekdays): 6-8h × depression symptoms × pandemic | 0.97 (0.27-3.52) | -0.03 (0.66) | 0.966 |
|  | sleep hours (weekdays): 6-8h × pandemic | 0.54 (0.26-1.13) | -0.62 (0.38) | 0.117 |
|  | sleep hours (weekdays)≥8h × depression symptoms | 0.64 (0.32-1.28) | -0.45 (0.36) | 0.218 |
|  | sleep hours (weekdays)≥8h × depression symptoms × pandemic | 1.18 (0.34-4.15) | 0.17 (0.64) | 0.799 |
|  | sleep hours (weekdays)≥8h × pandemic | 0.69 (0.29-1.67) | -0.36 (0.45) | 0.423 |
|  | multimorbidity × men × pre-pandemic | 1.07 (0.69-1.68) | 0.07 (0.23) | 0.756 |
|  | multimorbidity × men × pandemic | 1.14 (0.63-2.04) | 0.13 (0.30) | 0.669 |
|  | multimorbidity × women × pandemic | 0.51 (0.32-0.79) | -0.68 (0.23) | 0.006 |
|  | non-multimorbidity × men × pandemic | 1.00 (0.56-1.76) | -0.00 (0.29) | 0.991 |
|  | multimorbidity × pandemic | 0.54 (0.40-0.74) | -0.61 (0.16) | 0.001 |
|  | multimorbidity × full-time employee × pre-pandemic | 1.08 (0.69-1.67) | 0.07 (0.22) | 0.749 |
|  | multimorbidity × full-time employee × pandemic | 0.89 (0.44-1.82) | -0.12 (0.36) | 0.755 |
|  | multimorbidity × not-employed × pandemic | 0.52 (0.34-0.81) | -0.65 (0.22) | 0.008 |
|  | multimorbidity × part-time employee × pre-pandemic | 0.78 (0.38-1.61) | -0.25 (0.37) | 0.51 |
|  | multimorbidity × part-time employee × pandemic | 1.73 (0.54-5.62) | 0.55 (0.60) | 0.368 |
|  | non-multimorbidity × full-time employee × pandemic | 0.88 (0.54-1.44) | -0.13 (0.25) | 0.619 |
|  | part-time employee × pandemic | 0.95 (0.39-2.33) | -0.05 (0.46) | 0.914 |
|  | multimorbidity × alcohol consumption × pre-pandemic | 1.15 (0.72-1.84) | 0.14 (0.24) | 0.56 |
|  | multimorbidity × alcohol consumption × pandemic | 1.12 (0.63-2.01) | 0.12 (0.30) | 0.697 |
|  | non-multimorbidity × alcohol consumption × pandemic | 0.95 (0.50-1.77) | -0.06 (0.32) | 0.862 |
|  | multimorbidity × born outside the US × pre-pandemic | 0.70 (0.44-1.12) | -0.36 (0.24) | 0.152 |
|  | multimorbidity × born outside the US × pandemic | 2.03 (1.06-3.89) | 0.71 (0.33) | 0.041 |
|  | multimorbidity × US-born × pandemic | 0.46 (0.32-0.66) | -0.77 (0.18) | ＜0.001 |
|  | non-multimorbidity × born outside the US × pandemic | 0.51 (0.30-0.84) | -0.68 (0.26) | 0.014 |
|  | multimorbidity × CDAI × pre-pandemic | 0.71 (0.59-0.87) | -0.34 (0.10) | 0.002 |
|  | multimorbidity × CDAI × pandemic | 1.59 (1.16-2.17) | 0.46 (0.16) | 0.008 |
|  | non-multimorbidity × CDAI × pandemic | 0.81 (0.60-1.09) | -0.21 (0.15) | 0.17 |
|  | multimorbidity × depression symptoms × pre-pandemic | 1.30 (0.63-2.67) | 0.26 (0.37) | 0.485 |
|  | multimorbidity × depression symptoms × pandemic | 1.22 (0.46-3.24) | 0.20 (0.50) | 0.694 |
|  | multimorbidity × no/minimal depression × pandemic | 0.46 (0.31-0.68) | -0.77 (0.20) | 0.001 |
|  | non-multimorbidity × depression symptoms × pandemic | 1.17 (0.55-2.52) | 0.16 (0.39) | 0.683 |
|  | multimorbidity × DII × pre-pandemic | 1.24 (0.89-1.74) | 0.22 (0.17) | 0.221 |
|  | multimorbidity × DII × pandemic | 0.70 (0.47-1.03) | -0.36 (0.20) | 0.083 |
|  | non-multimorbidity × DII × pandemic | 1.18 (0.90-1.54) | 0.17 (0.14) | 0.238 |
|  | multimorbidity × college graduate or above × pre-pandemic | 1.13 (0.66-1.94) | 0.12 (0.28) | 0.664 |
|  | multimorbidity × college graduate or above × pandemic | 0.66 (0.27-1.61) | -0.42 (0.46) | 0.368 |
|  | multimorbidity × less than college × pandemic | 0.68 (0.40-1.15) | -0.38 (0.27) | 0.168 |
|  | multimorbidity × some college × pre-pandemic | 1.28 (0.67-2.42) | 0.24 (0.33) | 0.466 |
|  | multimorbidity × some college × pandemic | 0.68 (0.29-1.62) | -0.39 (0.44) | 0.392 |
|  | non-multimorbidity × college graduate or above × pandemic | 0.95 (0.46-1.97) | -0.05 (0.37) | 0.89 |
|  | non-multimorbidity × some college × pandemic | 1.62 (0.81-3.26) | 0.48 (0.36) | 0.186 |
|  | multimorbidity × health insurance × pre-pandemic | 0.95 (0.44-2.07) | -0.05 (0.40) | 0.908 |
|  | multimorbidity × health insurance × pandemic | 1.41 (0.41-4.84) | 0.34 (0.63) | 0.593 |
|  | multimorbidity × no health insurance × pandemic | 0.39 (0.13-1.22) | -0.93 (0.58) | 0.117 |
|  | non-multimorbidity × health insurance × pandemic | 0.97 (0.38-2.50) | -0.03 (0.48) | 0.954 |
|  | multimorbidity × divorced, separated or widowed × pre-pandemic | 0.73 (0.33-1.61) | -0.32 (0.41) | 0.441 |
|  | multimorbidity × divorced, separated or widowed × pandemic | 0.52 (0.17-1.63) | -0.65 (0.58) | 0.273 |
|  | multimorbidity × married or living with partner × pre-pandemic | 0.85 (0.46-1.59) | -0.16 (0.32) | 0.621 |
|  | multimorbidity × married or living with partner × pandemic | 0.53 (0.19-1.46) | -0.64 (0.52) | 0.23 |
|  | multimorbidity × never married × pandemic | 0.97 (0.44-2.16) | -0.03 (0.41) | 0.943 |
|  | non-multimorbidity × divorced, separated or widowed × pandemic | 1.20 (0.53-2.73) | 0.18 (0.42) | 0.667 |
|  | non-multimorbidity × married or living with partner × pandemic | 1.05 (0.48-2.31) | 0.05 (0.40) | 0.903 |
|  | multimorbidity × high active × pre-pandemic | 1.00 (0.53-1.87) | -0.00 (0.32) | 0.993 |
|  | multimorbidity × high active × pandemic | 0.93 (0.41-2.09) | -0.07 (0.41) | 0.863 |
|  | multimorbidity × moderate active × pre-pandemic | 1.62 (0.81-3.27) | 0.48 (0.36) | 0.187 |
|  | multimorbidity × moderate active × pandemic | 0.65 (0.24-1.72) | -0.44 (0.50) | 0.391 |
|  | multimorbidity × low active × pandemic | 0.59 (0.39-0.87) | -0.53 (0.20) | 0.015 |
|  | non-multimorbidity × high active × pandemic | 1.18 (0.55-2.51) | 0.16 (0.39) | 0.678 |
|  | non-multimorbidity × moderate active × pandemic | 0.98 (0.43-2.24) | -0.02 (0.42) | 0.965 |
|  | multimorbidity × high income × pre-pandemic | 1.72 (0.71-4.18) | 0.54 (0.45) | 0.243 |
|  | multimorbidity × high income × pandemic | 0.32 (0.11-0.91) | -1.14 (0.53) | 0.044 |
|  | multimorbidity × middle income × pre-pandemic | 1.49 (0.78-2.85) | 0.40 (0.33) | 0.24 |
|  | multimorbidity × middle income × pandemic | 0.39 (0.15-1.05) | -0.93 (0.50) | 0.075 |
|  | multimorbidity × low income × pandemic | 1.29 (0.64-2.59) | 0.25 (0.36) | 0.485 |
|  | non-multimorbidity × high income × pandemic | 1.34 (0.61-2.92) | 0.29 (0.40) | 0.47 |
|  | non-multimorbidity × middle income × pandemic | 2.10 (1.06-4.14) | 0.74 (0.35) | 0.044 |
|  | multimorbidity × sleep hours (weekdays) <6h × pandemic | 0.59 (0.17-2.03) | -0.52 (0.63) | 0.416 |
|  | multimorbidity × sleep hours (weekdays): 6-8h × pre-pandemic | 1.00 (0.29-3.45) | -0.00 (0.63) | 0.997 |
|  | multimorbidity × sleep hours (weekdays): 6-8h × pre-pandemic | 1.18 (0.44-3.21) | 0.17 (0.51) | 0.744 |
|  | multimorbidity × sleep hours (weekdays): 6-8h × pandemic | 1.12 (0.24-5.28) | 0.11 (0.79) | 0.892 |
|  | multimorbidity × sleep hours (weekdays): 6-8h × pandemic | 0.69 (0.19-2.54) | -0.37 (0.66) | 0.585 |
|  | sleep hours (weekdays): 6-8h × pandemic | 0.46 (0.15-1.40) | -0.79 (0.57) | 0.184 |
|  | sleep hours (weekdays): 6-8h × pandemic | 0.88 (0.30-2.54) | -0.13 (0.54) | 0.809 |
|  | multimorbidity × sleep hours (weekends) <6h × pandemic | 0.43 (0.19-0.94) | -0.85 (0.40) | 0.045 |
|  | multimorbidity × sleep hours (weekends) ≥ 8h × pre-pandemic | 1.06 (0.72-1.56) | 0.06 (0.20) | 0.772 |
|  | multimorbidity × sleep hours (weekends) ≥ 8h × pandemic | 1.12 (0.50-2.50) | 0.11 (0.41) | 0.789 |
|  | multimorbidity × sleep hours (weekends): 6-8h × pre-pandemic | 0.76 (0.41-1.40) | -0.28 (0.31) | 0.383 |
|  | multimorbidity × sleep hours (weekends): 6-8h × pandemic | 1.77 (0.56-5.58) | 0.57 (0.59) | 0.34 |
|  | sleep hours (weekends) ≥ 8h × pandemic | 0.78 (0.45-1.33) | -0.25 (0.28) | 0.365 |
|  | sleep hours (weekends): 6-8h × pandemic | 0.69 (0.28-1.70) | -0.37 (0.46) | 0.428 |
|  | multimorbidity × current smoker × pre-pandemic | 0.98 (0.50-1.93) | -0.02 (0.35) | 0.96 |
|  | multimorbidity × current smoker × pandemic | 1.46 (0.57-3.78) | 0.38 (0.48) | 0.441 |
|  | multimorbidity × never-smoker × pandemic | 0.55 (0.36-0.85) | -0.59 (0.22) | 0.012 |
|  | multimorbidity × ex-smoker × pre-pandemic | 1.45 (0.79-2.66) | 0.37 (0.31) | 0.242 |
|  | multimorbidity × ex-smoker × pandemic | 0.68 (0.28-1.64) | -0.38 (0.45) | 0.404 |
|  | non-multimorbidity × current smoker × pandemic | 1.50 (0.70-3.21) | 0.40 (0.39) | 0.31 |
|  | non-multimorbidity × ex-smoker × pandemic | 1.49 (0.76-2.92) | 0.40 (0.34) | 0.262 |
|  | men × age × multimorbidity × pandemic | 1.02 (0.97-1.07) | 0.02 (0.03) | 0.453 |

Model was adjusted for age, sex, and race. Reference groups: pre-pandemic period, less than college, never married, men, never-smoker, US-born, low income, not employed, no/minimal depression, no health insurance, low active, 20-39 years old, non-obese, non-hypertension, and <6 hours sleep. CDAI: composite dietary antioxidant index; DII: dietary inflammatory index; ROR, ratio of odds ratios; T2D, type 2 diabetes.

**Table S5.** Regression analyses from the multiple imputation sensitivity analysis.

| Group | Variables |  | Prediabetes | | | | T2D | | | |
| --- | --- | --- | --- | --- | --- | --- | --- | --- | --- | --- |
|  |  |  | 2017-2020 | | 2021-2023 | | 2017-2020 | | 2021-2023 | |
|  |  |  | OR （95%CI） | P-value | OR （95%CI） | P-value | OR （95%CI） | P-value | OR （95%CI） | P-value |
| Total | Educational levels | Less than college | Reference |  | Reference |  | Reference |  | Reference |  |
|  |  | Some college | 0.97 (0.76-1.24) | 0.783 | 0.96 (0.73-1.27) | 0.747 | 0.74 (0.55-1.01) | 0.057 | 0.98 (0.75-1.28) | 0.853 |
|  |  | College graduate or above | 1.16 (0.88-1.52) | 0.284 | 0.92 (0.66-1.28) | 0.550 | 0.55 (0.36-0.83) | 0.008 | 0.49 (0.31-0.77) | 0.009 |
|  | Married status | Never married | Reference |  | Reference |  | Reference |  | Reference |  |
|  |  | Divorced, separated or widowed | 1.59 (1.10-2.29) | 0.017 | 1.12 (0.78-1.61) | 0.481 | 1.11 (0.67-1.85) | 0.663 | 1.06 (0.55-2.01) | 0.840 |
|  |  | Married or living with partner | 1.26 (0.91-1.74) | 0.148 | 1.03 (0.73-1.46) | 0.828 | 1.37 (0.88-2.15) | 0.150 | 1.21 (0.70-2.08) | 0.420 |
|  | Place of birth | US-born | Reference |  | Reference |  | Reference |  | Reference |  |
|  |  | Born outside the US | 1.33 (1.05-1.67) | 0.019 | 1.25 (0.92-1.69) | 0.129 | 1.09 (0.65-1.83) | 0.726 | 0.81 (0.47-1.41) | 0.391 |
|  | PIR levels | Low income | Reference |  | Reference |  | Reference |  | Reference |  |
|  |  | Middle income | 1.17 (0.92-1.47) | 0.180 | 1.00 (0.77-1.32) | 0.965 | 0.81 (0.68-0.97) | 0.025 | 1.02 (0.71-1.48) | 0.894 |
|  |  | High income | 1.09 (0.86-1.38) | 0.463 | 1.27 (0.95-1.68) | 0.087 | 0.77 (0.57-1.02) | 0.069 | 0.58 (0.34-0.99) | 0.048 |
|  | Work | Not-employed | Reference |  | Reference |  | Reference |  | Reference |  |
|  |  | Part-time employee | 1.09 (0.80-1.50) | 0.551 | 1.13 (0.76-1.68) | 0.483 | 0.69 (0.45-1.06) | 0.088 | 0.71 (0.46-1.08) | 0.093 |
|  |  | Full-time employee | 1.32 (1.03-1.70) | 0.033 | 1.18 (0.89-1.58) | 0.201 | 1.02 (0.73-1.41) | 0.917 | 0.79 (0.62-1.02) | 0.062 |
|  | Health insurance | No | Reference |  | Reference |  | Reference |  | Reference |  |
|  |  | Yes | 1.04 (0.79-1.38) | 0.759 | 1.13 (0.74-1.72) | 0.521 | 0.99 (0.72-1.37) | 0.958 | 1.26 (0.54-2.95) | 0.529 |
|  | Depression levels | No/minimal depression | Reference |  | Reference |  | Reference |  | Reference |  |
|  |  | Depression-symptoms | 1.01 (0.78-1.31) | 0.937 | 0.97 (0.74-1.26) | 0.768 | 1.22 (0.98-1.52) | 0.068 | 1.39 (0.99-1.95) | 0.058 |
|  | Smoking status | Never | Reference |  | Reference |  | Reference |  | Reference |  |
|  |  | Ex-smoker | 1.08 (0.86-1.37) | 0.474 | 0.98 (0.73-1.34) | 0.904 | 1.23 (0.78-1.94) | 0.343 | 1.26 (0.85-1.87) | 0.191 |
|  |  | Current smoker | 1.29 (1.07-1.56) | 0.012 | 1.66 (1.12-2.47) | 0.020 | 1.10 (0.69-1.75) | 0.670 | 1.65 (1.12-2.44) | 0.020 |
|  | PA_levels | Low active | Reference |  | Reference |  | Reference |  | Reference |  |
|  |  | Moderate active | 0.90 (0.63-1.28) | 0.538 | 0.93 (0.70-1.24) | 0.573 | 1.10 (0.70-1.72) | 0.658 | 0.77 (0.56-1.06) | 0.088 |
|  |  | High active | 0.84 (0.63-1.13) | 0.235 | 0.93 (0.68-1.26) | 0.571 | 0.68 (0.47-0.98) | 0.041 | 0.77 (0.43-1.37) | 0.299 |
|  | Sleep hours weekdays | <6h | Reference |  | Reference |  | Reference |  | Reference |  |
|  |  | 6-8h | 0.71 (0.53-0.95) | 0.024 | 0.95 (0.62-1.45) | 0.774 | 1.17 (0.78-1.75) | 0.424 | 0.64 (0.33-1.25) | 0.150 |
|  |  | ≥8h | 0.59 (0.39-0.89) | 0.016 | 0.95 (0.56-1.63) | 0.822 | 0.96 (0.58-1.58) | 0.872 | 0.79 (0.36-1.76) | 0.496 |
|  | Sleep hours weekends | <6h | Reference |  | Reference |  | Reference |  | Reference |  |
|  |  | 6-8h | 1.15 (0.86-1.52) | 0.319 | 0.92 (0.63-1.33) | 0.588 | 0.90 (0.61-1.32) | 0.561 | 0.98 (0.59-1.64) | 0.935 |
|  |  | ≥8h | 0.87 (0.66-1.14) | 0.288 | 0.95 (0.70-1.28) | 0.672 | 0.81 (0.60-1.08) | 0.136 | 0.80 (0.56-1.13) | 0.163 |
|  | Energy intake, kcal |  | 1.12 (1.03-1.22) | 0.014 | 1.08 (0.94-1.24) | 0.230 | 0.91 (0.79-1.06) | 0.219 | 1.14 (0.90-1.45) | 0.228 |
|  | Alcohol_consumption_g_week |  | 1.02 (0.93-1.12) | 0.611 | 1.10 (0.96-1.25) | 0.125 | 0.87 (0.66-1.14) | 0.272 | 0.90 (0.65-1.24) | 0.439 |
|  | CDAI |  | 1.06 (0.94-1.19) | 0.324 | 1.04 (0.93-1.17) | 0.391 | 0.89 (0.77-1.03) | 0.118 | 0.98 (0.81-1.20) | 0.852 |
|  | DII |  | 0.91 (0.81-1.03) | 0.119 | 0.96 (0.85-1.08) | 0.424 | 1.12 (0.99-1.27) | 0.078 | 1.02 (0.87-1.18) | 0.818 |
|  | Hypertension | No | Reference |  | Reference |  | Reference |  | Reference |  |
|  |  | Yes | 1.01 (0.81-1.25) | 0.947 | 0.96 (0.65-1.41) | 0.803 | 2.56 (1.89-3.45) | <0.001 | 2.10 (1.56-2.83) | <0.001 |
|  | Obesity | No | Reference |  | Reference |  | Reference |  | Reference |  |
|  |  | Yes | 0.86 (0.64-1.15) | 0.284 | 1.01 (0.69-1.47) | 0.972 | 1.89 (1.35-2.64) | <0.001 | 1.70 (1.13-2.54) | 0.018 |
|  | Multimorbidity | No | Reference |  | Reference |  | Reference |  | Reference |  |
|  |  | Yes | 0.98 (0.76-1.27) | 0.866 | 0.99 (0.78-1.25) | 0.900 | 2.53 (1.81-3.54) | <0.001 | 1.51 (1.12-2.04) | 0.015 |
| Men | Educational levels | Less than college | Reference |  | Reference |  | Reference |  | Reference |  |
|  |  | Some college | 1.13 (0.77-1.68) | 0.507 | 0.91 (0.52-1.58) | 0.672 | 0.79 (0.50-1.25) | 0.294 | 1.11 (0.80-1.54) | 0.466 |
|  |  | College graduate or above | 1.38 (0.93-2.04) | 0.103 | 0.82 (0.48-1.39) | 0.386 | 0.55 (0.32-0.94) | 0.032 | 0.58 (0.36-0.93) | 0.030 |
|  | Married status | Never married | Reference |  | Reference |  | Reference |  | Reference |  |
|  |  | Divorced, separated or widowed | 1.48 (0.91-2.42) | 0.106 | 1.13 (0.60-2.12) | 0.642 | 0.89 (0.48-1.66) | 0.693 | 1.57 (0.63-3.92) | 0.267 |
|  |  | Married or living with partner | 1.15 (0.71-1.87) | 0.549 | 1.12 (0.69-1.81) | 0.583 | 1.09 (0.59-2.00) | 0.768 | 1.72 (0.70-4.20) | 0.185 |
|  | Place of birth | US-born | Reference |  | Reference |  | Reference |  | Reference |  |
|  |  | Born outside the US | 1.12 (0.78-1.61) | 0.507 | 1.30 (0.79-2.15) | 0.253 | 0.93 (0.39-2.20) | 0.855 | 0.88 (0.49-1.59) | 0.628 |
|  | PIR levels | Low income | Reference |  | Reference |  | Reference |  | Reference |  |
|  |  | Middle income | 1.12 (0.71-1.76) | 0.598 | 1.21 (0.71-2.07) | 0.413 | 0.85 (0.57-1.27) | 0.407 | 1.21 (0.65-2.24) | 0.484 |
|  |  | High income | 1.16 (0.85-1.58) | 0.338 | 1.53 (1.05-2.25) | 0.034 | 0.77 (0.47-1.26) | 0.278 | 0.65 (0.30-1.41) | 0.221 |
|  | Work | Not-employed | Reference |  | Reference |  | Reference |  | Reference |  |
|  |  | Part-time employee | 1.27 (0.76-2.12) | 0.338 | 1.36 (0.74-2.50) | 0.262 | 0.82 (0.43-1.59) | 0.541 | 0.37 (0.14-1.01) | 0.051 |
|  |  | Full-time employee | 1.36 (0.90-2.06) | 0.131 | 1.21 (0.90-1.62) | 0.166 | 0.87 (0.51-1.49) | 0.586 | 0.77 (0.48-1.25) | 0.235 |
|  | Health insurance | No | Reference |  | Reference |  | Reference |  | Reference |  |
|  |  | Yes | 1.32 (0.91-1.91) | 0.136 | 1.42 (0.73-2.76) | 0.246 | 1.69 (1.05-2.73) | 0.033 | 1.39 (0.45-4.28) | 0.507 |
|  | Depression levels | No/minimal depression | Reference |  | Reference |  | Reference |  | Reference |  |
|  |  | Depression-symptoms | 0.92 (0.66-1.28) | 0.598 | 1.06 (0.71-1.59) | 0.752 | 1.03 (0.66-1.61) | 0.877 | 1.42 (0.78-2.57) | 0.203 |
|  | Smoking status | Never | Reference |  | Reference |  | Reference |  | Reference |  |
|  |  | Ex-smoker | 1.09 (0.73-1.62) | 0.663 | 0.99 (0.64-1.51) | 0.938 | 1.19 (0.70-2.01) | 0.501 | 1.24 (0.67-2.30) | 0.415 |
|  |  | Current smoker | 1.15 (0.85-1.56) | 0.343 | 2.23 (1.39-3.58) | 0.006 | 0.88 (0.60-1.28) | 0.470 | 1.36 (0.77-2.41) | 0.227 |
|  | PA_levels | Low active | Reference |  | Reference |  | Reference |  | Reference |  |
|  |  | Moderate active | 0.87 (0.57-1.33) | 0.498 | 0.96 (0.55-1.68) | 0.871 | 1.31 (0.72-2.37) | 0.354 | 0.75 (0.39-1.47) | 0.334 |
|  |  | High active | 0.90 (0.62-1.30) | 0.551 | 0.85 (0.54-1.35) | 0.427 | 0.69 (0.42-1.13) | 0.128 | 0.66 (0.29-1.54) | 0.274 |
|  | Sleep hours weekdays | <6h | Reference |  | Reference |  | Reference |  | Reference |  |
|  |  | 6-8h | 0.54 (0.36-0.81) | 0.005 | 0.97 (0.61-1.56) | 0.896 | 1.46 (0.88-2.42) | 0.136 | 0.61 (0.24-1.58) | 0.250 |
|  |  | ≥8h | 0.55 (0.32-0.97) | 0.039 | 0.84 (0.41-1.71) | 0.562 | 1.18 (0.63-2.24) | 0.584 | 0.80 (0.30-2.18) | 0.604 |
|  | Sleep hours weekends | <6h | Reference |  | Reference |  | Reference |  | Reference |  |
|  |  | 6-8h | 1.27 (0.85-1.89) | 0.221 | 0.77 (0.36-1.68) | 0.445 | 0.81 (0.44-1.48) | 0.471 | 1.08 (0.52-2.20) | 0.810 |
|  |  | ≥8h | 0.99 (0.70-1.41) | 0.958 | 0.92 (0.50-1.70) | 0.742 | 0.81 (0.39-1.66) | 0.540 | 0.67 (0.36-1.25) | 0.165 |
|  | Energy intake, kcal |  | 1.11 (0.98-1.25) | 0.097 | 0.96 (0.76-1.20) | 0.672 | 0.82 (0.67-0.99) | 0.043 | 1.13 (0.86-1.49) | 0.333 |
|  | Alcohol_consumption_g_week |  | 1.00 (0.88-1.12) | 0.935 | 1.12 (0.95-1.31) | 0.135 | 0.84 (0.62-1.13) | 0.219 | 0.86 (0.58-1.28) | 0.373 |
|  | CDAI |  | 1.06 (0.93-1.20) | 0.362 | 0.94 (0.76-1.15) | 0.482 | 0.94 (0.80-1.11) | 0.466 | 1.08 (0.83-1.41) | 0.517 |
|  | DII |  | 0.90 (0.78-1.04) | 0.138 | 1.03 (0.85-1.25) | 0.695 | 1.19 (1.00-1.42) | 0.044 | 1.01 (0.80-1.28) | 0.925 |
|  | Hypertension | No | Reference |  | Reference |  | Reference |  | Reference |  |
|  |  | Yes | 1.01 (0.78-1.31) | 0.922 | 0.98 (0.59-1.63) | 0.938 | 2.20 (1.50-3.23) | <0.001 | 1.77 (1.10-2.86) | 0.026 |
|  | Obesity | No | Reference |  | Reference |  | Reference |  | Reference |  |
|  |  | Yes | 0.81 (0.49-1.35) | 0.392 | 1.27 (0.66-2.45) | 0.409 | 1.41 (0.78-2.56) | 0.240 | 1.63 (0.95-2.78) | 0.069 |
|  | Multimorbidity | No | Reference |  | Reference |  | Reference |  | Reference |  |
|  |  | Yes | 0.89 (0.63-1.27) | 0.497 | 1.06 (0.76-1.48) | 0.677 | 2.48 (1.44-4.28) | 0.003 | 1.68 (1.26-2.23) | 0.004 |
| Women | Educational levels | Less than college | Reference |  | Reference |  | Reference |  | Reference |  |
|  |  | Some college | 0.86 (0.62-1.18) | 0.317 | 1.05 (0.72-1.52) | 0.775 | 0.70 (0.44-1.12) | 0.127 | 0.87 (0.60-1.25) | 0.378 |
|  |  | College graduate or above | 1.02 (0.78-1.34) | 0.875 | 1.03 (0.67-1.60) | 0.864 | 0.49 (0.27-0.87) | 0.019 | 0.39 (0.20-0.75) | 0.013 |
|  | Married status | Never married | Reference |  | Reference |  | Reference |  | Reference |  |
|  |  | Divorced, separated or widowed | 1.95 (0.95-3.99) | 0.065 | 1.16 (0.67-1.99) | 0.527 | 1.61 (0.92-2.82) | 0.092 | 0.83 (0.43-1.61) | 0.512 |
|  |  | Married or living with partner | 1.51 (0.88-2.59) | 0.123 | 0.99 (0.53-1.85) | 0.960 | 1.58 (1.01-2.47) | 0.048 | 0.82 (0.49-1.38) | 0.385 |
|  | Place of birth | US-born | Reference |  | Reference |  | Reference |  | Reference |  |
|  |  | Born outside the US | 1.57 (1.08-2.28) | 0.022 | 1.20 (0.74-1.94) | 0.401 | 1.32 (0.82-2.12) | 0.233 | 0.73 (0.36-1.48) | 0.320 |
|  | PIR levels | Low income | Reference |  | Reference |  | Reference |  | Reference |  |
|  |  | Middle income | 1.19 (0.85-1.66) | 0.288 | 0.90 (0.62-1.29) | 0.486 | 0.75 (0.52-1.08) | 0.119 | 0.88 (0.55-1.41) | 0.531 |
|  |  | High income | 0.97 (0.70-1.33) | 0.826 | 1.09 (0.71-1.67) | 0.632 | 0.68 (0.44-1.05) | 0.076 | 0.47 (0.24-0.91) | 0.031 |
|  | Work | Not-employed | Reference |  | Reference |  | Reference |  | Reference |  |
|  |  | Part-time employee | 1.02 (0.62-1.68) | 0.941 | 0.99 (0.48-2.02) | 0.971 | 0.62 (0.29-1.33) | 0.200 | 1.12 (0.44-2.84) | 0.773 |
|  |  | Full-time employee | 1.20 (0.86-1.66) | 0.258 | 1.09 (0.70-1.72) | 0.638 | 1.09 (0.74-1.62) | 0.640 | 0.72 (0.50-1.04) | 0.073 |
|  | Health insurance | No | Reference |  | Reference |  | Reference |  | Reference |  |
|  |  | Yes | 0.82 (0.57-1.18) | 0.266 | 0.87 (0.48-1.58) | 0.596 | 0.58 (0.34-0.97) | 0.039 | 1.18 (0.37-3.78) | 0.738 |
|  | Depression levels | No/minimal depression | Reference |  | Reference |  | Reference |  | Reference |  |
|  |  | Depression-symptoms | 1.13 (0.81-1.56) | 0.452 | 0.92 (0.69-1.22) | 0.490 | 1.63 (1.18-2.25) | 0.006 | 1.51 (1.02-2.23) | 0.042 |
|  | Smoking status | Never | Reference |  | Reference |  | Reference |  | Reference |  |
|  |  | Ex-smoker | 0.98 (0.75-1.27) | 0.856 | 0.97 (0.73-1.29) | 0.785 | 1.00 (0.50-2.03) | 0.991 | 1.12 (0.65-1.94) | 0.625 |
|  |  | Current smoker | 1.43 (1.10-1.86) | 0.011 | 1.05 (0.55-2.03) | 0.853 | 1.32 (0.64-2.71) | 0.427 | 1.99 (1.12-3.53) | 0.027 |
|  | PA_levels | Low active | Reference |  | Reference |  | Reference |  | Reference |  |
|  |  | Moderate active | 0.94 (0.56-1.58) | 0.812 | 0.90 (0.62-1.29) | 0.488 | 0.78 (0.39-1.54) | 0.446 | 0.71 (0.37-1.36) | 0.236 |
|  |  | High active | 0.70 (0.47-1.04) | 0.077 | 0.99 (0.71-1.38) | 0.932 | 0.52 (0.31-0.88) | 0.018 | 0.77 (0.46-1.30) | 0.267 |
|  | Sleep hours weekdays | <6h | Reference |  | Reference |  | Reference |  | Reference |  |
|  |  | 6-8h | 1.05 (0.70-1.57) | 0.814 | 0.91 (0.46-1.81) | 0.752 | 0.96 (0.46-1.99) | 0.900 | 0.75 (0.29-1.91) | 0.468 |
|  |  | ≥8h | 0.74 (0.49-1.11) | 0.135 | 1.05 (0.55-2.03) | 0.847 | 0.87 (0.38-1.97) | 0.716 | 0.89 (0.31-2.55) | 0.793 |
|  | Sleep hours weekends | <6h | Reference |  | Reference |  | Reference |  | Reference |  |
|  |  | 6-8h | 0.98 (0.66-1.44) | 0.895 | 1.05 (0.67-1.65) | 0.797 | 0.94 (0.63-1.42) | 0.761 | 0.83 (0.40-1.73) | 0.556 |
|  |  | ≥8h | 0.76 (0.55-1.05) | 0.090 | 0.95 (0.63-1.43) | 0.746 | 0.81 (0.53-1.25) | 0.320 | 0.95 (0.57-1.57) | 0.798 |
|  | Energy intake, kcal |  | 1.01 (0.83-1.23) | 0.916 | 1.31 (1.01-1.70) | 0.046 | 0.80 (0.61-1.05) | 0.106 | 0.85 (0.61-1.17) | 0.257 |
|  | Alcohol_consumption_g_week |  | 0.96 (0.71-1.30) | 0.773 | 1.01 (0.78-1.31) | 0.949 | 0.61 (0.20-1.83) | 0.295 | 0.78 (0.33-1.85) | 0.498 |
|  | CDAI |  | 1.05 (0.86-1.27) | 0.612 | 1.19 (0.98-1.45) | 0.068 | 0.81 (0.66-0.99) | 0.042 | 0.86 (0.66-1.14) | 0.245 |
|  | DII |  | 0.98 (0.79-1.21) | 0.825 | 0.90 (0.77-1.05) | 0.153 | 1.19 (0.99-1.43) | 0.060 | 1.15 (0.89-1.49) | 0.239 |
|  | Hypertension | No | Reference |  | Reference |  | Reference |  | Reference |  |
|  |  | Yes | 0.92 (0.63-1.35) | 0.666 | 0.95 (0.66-1.38) | 0.768 | 3.13 (2.15-4.57) | <0.001 | 2.55 (1.65-3.93) | 0.002 |
|  | Obesity | No | Reference |  | Reference |  | Reference |  | Reference |  |
|  |  | Yes | 0.88 (0.61-1.27) | 0.457 | 0.84 (0.52-1.36) | 0.420 | 2.87 (1.68-4.91) | <0.001 | 1.79 (1.04-3.08) | 0.038 |
|  | Multimorbidity | No | Reference |  | Reference |  | Reference |  | Reference |  |
|  |  | Yes | 1.10 (0.82-1.47) | 0.509 | 0.97 (0.69-1.35) | 0.806 | 2.79 (1.83-4.24) | <0.001 | 1.43 (0.89-2.31) | 0.118 |
| 20-39 years | Educational levels | Less than college | Reference |  | Reference |  | Reference |  | Reference |  |
|  |  | Some college | 1.16 (0.69-1.96) | 0.555 | 1.14 (0.43-2.99) | 0.753 | 0.58 (0.31-1.11) | 0.095 | 0.99 (0.34-2.86) | 0.986 |
|  |  | College graduate or above | 1.71 (0.91-3.21) | 0.092 | 0.65 (0.16-2.62) | 0.474 | 0.63 (0.21-1.92) | 0.396 | 0.39 (0.08-1.84) | 0.186 |
|  | Married status | Never married | Reference |  | Reference |  | Reference |  | Reference |  |
|  |  | Divorced, separated or widowed | 2.50 (1.24-5.05) | 0.014 | 2.22 (1.08-4.54) | 0.035 | 0.76 (0.07-8.25) | 0.811 | 0.00 (0.00-0.00) | <0.001 |
|  |  | Married or living with partner | 1.70 (1.13-2.55) | 0.014 | 1.17 (0.62-2.20) | 0.560 | 1.08 (0.51-2.26) | 0.835 | 1.25 (0.28-5.69) | 0.723 |
|  | Place of birth | US-born | Reference |  | Reference |  | Reference |  | Reference |  |
|  |  | Born outside the US | 0.98 (0.58-1.67) | 0.951 | 1.07 (0.66-1.73) | 0.754 | 6.03 (2.05-17.73) | 0.003 | 0.33 (0.06-1.95) | 0.182 |
|  | PIR levels | Low income | Reference |  | Reference |  | Reference |  | Reference |  |
|  |  | Middle income | 1.19 (0.77-1.84) | 0.397 | 0.76 (0.43-1.34) | 0.272 | 0.69 (0.36-1.34) | 0.257 | 0.86 (0.28-2.63) | 0.744 |
|  |  | High income | 1.35 (0.83-2.20) | 0.206 | 1.16 (0.43-3.10) | 0.721 | 0.62 (0.17-2.25) | 0.437 | 0.18 (0.02-1.37) | 0.083 |
|  | Work | Not-employed | Reference |  | Reference |  | Reference |  | Reference |  |
|  |  | Part-time employee | 0.93 (0.56-1.56) | 0.781 | 0.72 (0.25-2.06) | 0.465 | 0.62 (0.24-1.57) | 0.287 | 0.80 (0.13-4.99) | 0.778 |
|  |  | Full-time employee | 1.43 (0.85-2.41) | 0.160 | 0.67 (0.35-1.28) | 0.176 | 0.38 (0.14-1.00) | 0.050 | 0.72 (0.09-5.89) | 0.713 |
|  | Health insurance | No | Reference |  | Reference |  | Reference |  | Reference |  |
|  |  | Yes | 1.54 (1.02-2.34) | 0.041 | 1.43 (0.75-2.73) | 0.226 | 0.99 (0.40-2.45) | 0.981 | 0.65 (0.14-3.10) | 0.529 |
|  | Depression levels | No/minimal depression | Reference |  | Reference |  | Reference |  | Reference |  |
|  |  | Depression-symptoms | 1.24 (0.74-2.06) | 0.395 | 0.91 (0.59-1.39) | 0.610 | 1.17 (0.63-2.17) | 0.598 | 1.38 (0.45-4.24) | 0.519 |
|  | Smoking status | Never | Reference |  | Reference |  | Reference |  | Reference |  |
|  |  | Ex-smoker | 1.72 (0.87-3.38) | 0.110 | 0.93 (0.41-2.12) | 0.834 | 1.46 (0.43-4.92) | 0.521 | 3.55 (0.84-15.03) | 0.075 |
|  |  | Current smoker | 1.26 (0.89-1.79) | 0.184 | 2.02 (1.20-3.41) | 0.017 | 0.87 (0.33-2.31) | 0.764 | 3.07 (0.52-18.35) | 0.172 |
|  | PA_levels | Low active | Reference |  | Reference |  | Reference |  | Reference |  |
|  |  | Moderate active | 0.53 (0.26-1.08) | 0.078 | 0.70 (0.30-1.68) | 0.355 | 1.50 (0.26-8.56) | 0.627 | 0.29 (0.03-2.72) | 0.222 |
|  |  | High active | 0.62 (0.41-0.95) | 0.029 | 0.94 (0.57-1.53) | 0.746 | 0.67 (0.22-2.03) | 0.458 | 0.95 (0.15-6.10) | 0.947 |
|  | Sleep hours weekdays | <6h | Reference |  | Reference |  | Reference |  | Reference |  |
|  |  | 6-8h | 0.63 (0.31-1.25) | 0.168 | 1.15 (0.53-2.49) | 0.666 | 1.56 (0.59-4.13) | 0.348 | 0.22 (0.03-1.51) | 0.102 |
|  |  | ≥8h | 0.59 (0.26-1.31) | 0.179 | 1.41 (0.62-3.24) | 0.343 | 0.88 (0.32-2.45) | 0.800 | 0.64 (0.12-3.35) | 0.526 |
|  | Sleep hours weekends | <6h | Reference |  | Reference |  | Reference |  | Reference |  |
|  |  | 6-8h | 1.41 (0.79-2.52) | 0.226 | 1.01 (0.40-2.56) | 0.986 | 1.54 (0.66-3.59) | 0.295 | 0.96 (0.26-3.62) | 0.944 |
|  |  | ≥8h | 1.04 (0.61-1.79) | 0.872 | 1.06 (0.59-1.94) | 0.805 | 1.10 (0.50-2.40) | 0.798 | 0.16 (0.04-0.65) | 0.019 |
|  | Energy intake, kcal |  | 0.92 (0.72-1.17) | 0.454 | 1.17 (0.93-1.49) | 0.149 | 1.01 (0.78-1.32) | 0.915 | 0.64 (0.32-1.29) | 0.173 |
|  | Alcohol_consumption_g_week |  | 0.91 (0.75-1.11) | 0.335 | 0.85 (0.58-1.26) | 0.353 | 0.47 (0.12-1.90) | 0.249 | 0.81 (0.24-2.73) | 0.691 |
|  | CDAI |  | 0.93 (0.75-1.16) | 0.502 | 1.10 (0.85-1.41) | 0.411 | 1.14 (0.82-1.59) | 0.424 | 0.51 (0.27-0.95) | 0.037 |
|  | DII |  | 1.19 (0.95-1.49) | 0.115 | 0.95 (0.76-1.19) | 0.600 | 0.73 (0.48-1.11) | 0.133 | 2.09 (1.19-3.69) | 0.018 |
|  | Hypertension | No | Reference |  | Reference |  | Reference |  | Reference |  |
|  |  | Yes | 1.89 (1.33-2.69) | 0.001 | 1.16 (0.61-2.20) | 0.599 | 1.66 (0.58-4.74) | 0.323 | 5.98 (1.93-18.56) | 0.008 |
|  | Obesity | No | Reference |  | Reference |  | Reference |  | Reference |  |
|  |  | Yes | 1.07 (0.57-2.00) | 0.830 | 0.83 (0.42-1.64) | 0.536 | 1.64 (0.59-4.56) | 0.318 | 7.76 (0.98-61.65) | 0.052 |
|  | Multimorbidity | No | Reference |  | Reference |  | Reference |  | Reference |  |
|  |  | Yes | 1.52 (1.04-2.22) | 0.034 | 1.28 (0.73-2.24) | 0.334 | 2.94 (1.17-7.38) | 0.025 | 2.45 (0.92-6.50) | 0.066 |
| 40-59 years | Educational levels | Less than college | Reference |  | Reference |  | Reference |  | Reference |  |
|  |  | Some college | 0.98 (0.62-1.55) | 0.942 | 0.83 (0.49-1.40) | 0.402 | 0.50 (0.30-0.85) | 0.013 | 1.04 (0.51-2.09) | 0.906 |
|  |  | College graduate or above | 0.86 (0.56-1.31) | 0.463 | 0.95 (0.52-1.71) | 0.821 | 0.45 (0.26-0.79) | 0.009 | 0.47 (0.21-1.01) | 0.053 |
|  | Married status | Never married | Reference |  | Reference |  | Reference |  | Reference |  |
|  |  | Divorced, separated or widowed | 1.99 (1.05-3.77) | 0.037 | 0.99 (0.43-2.29) | 0.976 | 1.03 (0.43-2.51) | 0.937 | 1.43 (0.51-4.01) | 0.421 |
|  |  | Married or living with partner | 1.07 (0.63-1.82) | 0.799 | 0.77 (0.37-1.61) | 0.414 | 1.18 (0.61-2.28) | 0.592 | 1.10 (0.34-3.51) | 0.845 |
|  | Place of birth | US-born | Reference |  | Reference |  | Reference |  | Reference |  |
|  |  | Born outside the US | 1.76 (1.17-2.64) | 0.009 | 1.41 (0.77-2.57) | 0.217 | 0.84 (0.47-1.48) | 0.520 | 0.70 (0.26-1.86) | 0.416 |
|  | PIR levels | Low income | Reference |  | Reference |  | Reference |  | Reference |  |
|  |  | Middle income | 1.26 (0.84-1.90) | 0.242 | 0.87 (0.44-1.72) | 0.629 | 0.75 (0.46-1.23) | 0.235 | 1.10 (0.51-2.35) | 0.776 |
|  |  | High income | 0.83 (0.52-1.35) | 0.432 | 1.14 (0.53-2.48) | 0.686 | 0.68 (0.42-1.08) | 0.097 | 0.48 (0.25-0.90) | 0.030 |
|  | Work | Not-employed | Reference |  | Reference |  | Reference |  | Reference |  |
|  |  | Part-time employee | 1.07 (0.50-2.27) | 0.851 | 0.73 (0.42-1.28) | 0.215 | 0.62 (0.30-1.27) | 0.173 | 0.57 (0.16-2.02) | 0.314 |
|  |  | Full-time employee | 1.06 (0.65-1.73) | 0.810 | 1.01 (0.55-1.83) | 0.981 | 0.80 (0.53-1.20) | 0.257 | 0.47 (0.29-0.76) | 0.009 |
|  | Health insurance | No | Reference |  | Reference |  | Reference |  | Reference |  |
|  |  | Yes | 0.75 (0.47-1.19) | 0.205 | 1.13 (0.56-2.28) | 0.699 | 1.37 (0.71-2.65) | 0.327 | 1.72 (0.55-5.39) | 0.296 |
|  | Depression levels | No/minimal depression | Reference |  | Reference |  | Reference |  | Reference |  |
|  |  | Depression-symptoms | 1.29 (0.88-1.89) | 0.173 | 1.19 (0.71-1.99) | 0.455 | 1.14 (0.77-1.69) | 0.495 | 1.92 (0.99-3.71) | 0.052 |
|  | Smoking status | Never | Reference |  | Reference |  | Reference |  | Reference |  |
|  |  | Ex-smoker | 1.33 (0.82-2.16) | 0.236 | 0.94 (0.47-1.88) | 0.826 | 0.99 (0.57-1.72) | 0.975 | 1.13 (0.44-2.89) | 0.766 |
|  |  | Current smoker | 1.47 (1.01-2.14) | 0.045 | 1.34 (0.64-2.84) | 0.368 | 1.03 (0.58-1.83) | 0.913 | 1.60 (0.84-3.06) | 0.123 |
|  | PA_levels | Low active | Reference |  | Reference |  | Reference |  | Reference |  |
|  |  | Moderate active | 1.33 (0.75-2.38) | 0.310 | 0.85 (0.48-1.48) | 0.493 | 0.97 (0.54-1.75) | 0.909 | 0.88 (0.44-1.78) | 0.675 |
|  |  | High active | 0.88 (0.61-1.28) | 0.475 | 0.72 (0.49-1.06) | 0.082 | 0.60 (0.35-1.02) | 0.058 | 0.75 (0.38-1.49) | 0.343 |
|  | Sleep hours weekdays | <6h | Reference |  | Reference |  | Reference |  | Reference |  |
|  |  | 6-8h | 0.85 (0.52-1.39) | 0.481 | 1.14 (0.61-2.15) | 0.622 | 0.71 (0.36-1.42) | 0.310 | 0.41 (0.18-0.94) | 0.040 |
|  |  | ≥8h | 0.70 (0.42-1.19) | 0.171 | 1.15 (0.51-2.61) | 0.688 | 0.78 (0.39-1.57) | 0.463 | 0.50 (0.25-1.02) | 0.056 |
|  | Sleep hours weekends | <6h | Reference |  | Reference |  | Reference |  | Reference |  |
|  |  | 6-8h | 0.93 (0.60-1.47) | 0.755 | 0.69 (0.43-1.10) | 0.098 | 0.66 (0.33-1.33) | 0.227 | 1.27 (0.40-4.06) | 0.623 |
|  |  | ≥8h | 0.58 (0.34-1.00) | 0.049 | 0.91 (0.49-1.70) | 0.724 | 0.66 (0.34-1.29) | 0.205 | 0.88 (0.48-1.59) | 0.604 |
|  | Energy intake, kcal |  | 1.18 (0.97-1.44) | 0.090 | 0.90 (0.71-1.13) | 0.306 | 0.79 (0.58-1.06) | 0.111 | 1.14 (0.87-1.48) | 0.280 |
|  | Alcohol_consumption_g_week |  | 1.05 (0.88-1.24) | 0.582 | 1.11 (0.88-1.40) | 0.273 | 0.75 (0.43-1.33) | 0.295 | 1.00 (0.74-1.34) | 0.992 |
|  | CDAI |  | 1.13 (0.90-1.40) | 0.266 | 0.97 (0.78-1.21) | 0.761 | 0.87 (0.67-1.13) | 0.279 | 1.11 (0.85-1.44) | 0.393 |
|  | DII |  | 0.84 (0.71-1.00) | 0.051 | 1.02 (0.82-1.28) | 0.800 | 1.31 (1.05-1.62) | 0.019 | 0.93 (0.71-1.21) | 0.515 |
|  | Hypertension | No | Reference |  | Reference |  | Reference |  | Reference |  |
|  |  | Yes | 0.97 (0.67-1.40) | 0.855 | 0.88 (0.57-1.36) | 0.506 | 3.62 (2.26-5.80) | <0.001 | 2.21 (1.29-3.77) | 0.010 |
|  | Obesity | No | Reference |  | Reference |  | Reference |  | Reference |  |
|  |  | Yes | 0.71 (0.43-1.19) | 0.180 | 1.40 (0.87-2.26) | 0.132 | 2.30 (1.00-5.31) | 0.051 | 2.18 (0.79-6.02) | 0.110 |
|  | Multimorbidity | No | Reference |  | Reference |  | Reference |  | Reference |  |
|  |  | Yes | 1.11 (0.69-1.79) | 0.640 | 1.02 (0.70-1.48) | 0.906 | 3.14 (2.08-4.74) | <0.001 | 1.89 (1.22-2.91) | 0.011 |
| ≥60 years | Educational levels | Less than college | Reference |  | Reference |  | Reference |  | Reference |  |
|  |  | Some college | 0.84 (0.64-1.11) | 0.206 | 0.94 (0.74-1.19) | 0.515 | 1.09 (0.65-1.82) | 0.733 | 0.94 (0.67-1.33) | 0.678 |
|  |  | College graduate or above | 1.27 (0.86-1.87) | 0.205 | 1.09 (0.85-1.39) | 0.415 | 0.54 (0.32-0.92) | 0.027 | 0.53 (0.27-1.03) | 0.059 |
|  | Married status | Never married | Reference |  | Reference |  | Reference |  | Reference |  |
|  |  | Divorced, separated or widowed | 0.43 (0.23-0.83) | 0.015 | 1.14 (0.63-2.07) | 0.606 | 2.09 (0.94-4.64) | 0.067 | 1.15 (0.53-2.50) | 0.667 |
|  |  | Married or living with partner | 0.39 (0.19-0.84) | 0.019 | 1.11 (0.59-2.10) | 0.690 | 1.86 (0.88-3.95) | 0.099 | 1.16 (0.58-2.33) | 0.606 |
|  | Place of birth | US-born | Reference |  | Reference |  | Reference |  | Reference |  |
|  |  | Born outside the US | 1.60 (1.02-2.52) | 0.043 | 1.16 (0.65-2.06) | 0.565 | 0.72 (0.42-1.25) | 0.226 | 0.89 (0.51-1.54) | 0.622 |
|  | PIR levels | Low income | Reference |  | Reference |  | Reference |  | Reference |  |
|  |  | Middle income | 0.97 (0.53-1.79) | 0.920 | 1.62 (0.91-2.90) | 0.086 | 1.02 (0.68-1.52) | 0.925 | 1.02 (0.59-1.78) | 0.929 |
|  |  | High income | 1.03 (0.54-1.97) | 0.925 | 1.71 (0.97-3.01) | 0.060 | 0.77 (0.41-1.45) | 0.387 | 0.64 (0.32-1.29) | 0.167 |
|  | Work | Not-employed | Reference |  | Reference |  | Reference |  | Reference |  |
|  |  | Part-time employee | 1.20 (0.70-2.05) | 0.476 | 1.56 (0.95-2.57) | 0.072 | 0.55 (0.35-0.88) | 0.016 | 0.61 (0.34-1.10) | 0.087 |
|  |  | Full-time employee | 0.99 (0.68-1.44) | 0.957 | 1.17 (0.82-1.66) | 0.310 | 0.84 (0.52-1.36) | 0.456 | 0.70 (0.43-1.13) | 0.114 |
|  | Health insurance | No | Reference |  | Reference |  | Reference |  | Reference |  |
|  |  | Yes | 1.48 (0.74-2.93) | 0.245 | 1.20 (0.55-2.60) | 0.595 | 0.83 (0.45-1.53) | 0.526 | 1.19 (0.31-4.55) | 0.763 |
|  | Depression levels | No/minimal depression | Reference |  | Reference |  | Reference |  | Reference |  |
|  |  | Depression-symptoms | 0.68 (0.52-0.88) | 0.007 | 0.86 (0.58-1.30) | 0.416 | 1.49 (1.15-1.94) | 0.005 | 1.25 (0.72-2.16) | 0.372 |
|  | Smoking status | Never | Reference |  | Reference |  | Reference |  | Reference |  |
|  |  | Ex-smoker | 0.68 (0.51-0.91) | 0.013 | 1.06 (0.84-1.35) | 0.541 | 1.17 (0.65-2.11) | 0.572 | 1.14 (0.70-1.88) | 0.524 |
|  |  | Current smoker | 0.97 (0.58-1.60) | 0.892 | 1.52 (0.94-2.47) | 0.077 | 0.98 (0.55-1.72) | 0.925 | 1.16 (0.66-2.04) | 0.533 |
|  | PA_levels | Low active | Reference |  | Reference |  | Reference |  | Reference |  |
|  |  | Moderate active | 0.71 (0.47-1.07) | 0.094 | 1.11 (0.82-1.49) | 0.421 | 0.97 (0.53-1.77) | 0.918 | 0.75 (0.45-1.24) | 0.206 |
|  |  | High active | 1.00 (0.63-1.57) | 0.983 | 1.02 (0.69-1.52) | 0.890 | 0.59 (0.40-0.87) | 0.012 | 0.60 (0.34-1.06) | 0.068 |
|  | Sleep hours weekdays | <6h | Reference |  | Reference |  | Reference |  | Reference |  |
|  |  | 6-8h | 0.64 (0.34-1.20) | 0.151 | 0.71 (0.37-1.35) | 0.237 | 1.83 (0.88-3.82) | 0.100 | 1.51 (0.57-4.00) | 0.340 |
|  |  | ≥8h | 0.55 (0.30-1.01) | 0.052 | 0.67 (0.32-1.38) | 0.217 | 1.76 (0.90-3.42) | 0.092 | 1.97 (0.60-6.48) | 0.209 |
|  | Sleep hours weekends | <6h | Reference |  | Reference |  | Reference |  | Reference |  |
|  |  | 6-8h | 1.20 (0.73-1.97) | 0.450 | 1.05 (0.67-1.65) | 0.805 | 1.02 (0.73-1.42) | 0.922 | 0.80 (0.56-1.15) | 0.175 |
|  |  | ≥8h | 1.15 (0.74-1.81) | 0.507 | 0.90 (0.61-1.32) | 0.519 | 1.02 (0.73-1.42) | 0.915 | 0.89 (0.54-1.46) | 0.575 |
|  | Energy intake, kcal |  | 1.18 (0.96-1.44) | 0.103 | 1.11 (0.94-1.33) | 0.185 | 0.74 (0.63-0.88) | 0.002 | 0.92 (0.71-1.19) | 0.468 |
|  | Alcohol_consumption_g_week |  | 0.97 (0.74-1.26) | 0.776 | 1.14 (0.91-1.43) | 0.197 | 0.82 (0.60-1.11) | 0.179 | 0.63 (0.31-1.26) | 0.114 |
|  | CDAI |  | 1.12 (0.97-1.29) | 0.116 | 1.07 (0.98-1.17) | 0.118 | 0.83 (0.70-0.99) | 0.040 | 0.91 (0.76-1.10) | 0.284 |
|  | DII |  | 0.84 (0.70-1.00) | 0.050 | 0.96 (0.85-1.08) | 0.404 | 1.22 (0.99-1.50) | 0.059 | 1.13 (0.96-1.32) | 0.114 |
|  | Hypertension | No | Reference |  | Reference |  | Reference |  | Reference |  |
|  |  | Yes | 0.86 (0.58-1.26) | 0.405 | 1.13 (0.78-1.62) | 0.464 | 2.24 (1.50-3.33) | <0.001 | 1.71 (1.02-2.87) | 0.043 |
|  | Obesity | No | Reference |  | Reference |  | Reference |  | Reference |  |
|  |  | Yes | 1.10 (0.66-1.82) | 0.707 | 0.94 (0.58-1.53) | 0.776 | 1.44 (0.95-2.20) | 0.082 | 1.13 (0.80-1.60) | 0.420 |
|  | Multimorbidity | No | Reference |  | Reference |  | Reference |  | Reference |  |
|  |  | Yes | 0.90 (0.64-1.27) | 0.517 | 1.07 (0.78-1.47) | 0.639 | 2.55 (1.77-3.67) | <0.001 | 1.45 (0.95-2.23) | 0.078 |

Model was adjusted for age, sex, and race. T2D, type 2 diabetes; PIR: poverty income ratio; CDAI: composite dietary antioxidant index; DII: dietary inflammatory index; PA, physical activity.

**Table S6.** Significant interaction effects from the multiple imputation sensitivity analysis.

| Disease | Interaction Term | ROR （95%CI） | β (SE) | P-interaction |
| --- | --- | --- | --- | --- |
| Prediabetes | DII × ≥60 years × pre-pandemic | 0.70 (0.49-0.98) | -0.36 (0.12) | 0.043 |
|  | DII × 40-59 years × pre-pandemic | 0.71 (0.52-0.98) | -0.34 (0.11) | 0.042 |
|  | divorced, separated or widowed × ≥60 years × pre-pandemic | 0.22 (0.05-0.91) | -1.52 (0.51) | 0.041 |
|  | married or living with partner × ≥60 years × pre-pandemic | 0.26 (0.09-0.72) | -1.35 (0.37) | 0.022 |
|  | married or living with partner × ≥60 years × pandemic | 4.17 (1.13-15.37) | 1.43 (0.47) | 0.038 |
|  | low active × 40-59 years × pandemic | 1.90 (1.10-3.28) | 0.64 (0.20) | 0.031 |
|  | high active × ≥60 years × pre-pandemic | 1.83 (1.12-2.99) | 0.60 (0.18) | 0.027 |
|  | moderate active × 40-59 years × pre-pandemic | 2.60 (1.08-6.22) | 0.95 (0.31) | 0.039 |
|  | ex-smoker × ≥60 years × pre-pandemic | 0.38 (0.15-0.99) | -0.96 (0.34) | 0.049 |
|  | multimorbidity × current smoker × pandemic | 0.29 (0.09-0.88) | -1.24 (0.40) | 0.036 |
|  | current smoker × pandemic | 2.14 (1.03-4.45) | 0.76 (0.26) | 0.045 |
| T2D | CDAI × ≥60 years × pandemic | 2.45 (1.11-5.41) | 0.90 (0.29) | 0.035 |
|  | CDAI × 40-59 years × pandemic | 2.86 (1.21-6.75) | 1.05 (0.31) | 0.028 |
|  | CDAI × pandemic | 0.45 (0.21-0.98) | -0.79 (0.28) | 0.047 |
|  | DII × ≥60 years × pandemic | 0.36 (0.16-0.82) | -1.01 (0.29) | 0.026 |
|  | DII × 40-59 years × pandemic | 0.28 (0.12-0.65) | -1.29 (0.31) | 0.014 |
|  | DII × pandemic | 2.53 (1.16-5.54) | 0.93 (0.28) | 0.03 |
|  | health insurance × male | 3.16 (1.22-8.18) | 1.15 (0.34) | 0.029 |
|  | obesity × male | 0.49 (0.28-0.87) | -0.70 (0.20) | 0.025 |
|  | born outside the US × 40-59 years | 0.29 (0.09-0.94) | -1.22 (0.42) | 0.043 |
|  | multimorbidity × pandemic | 0.61 (0.38-0.98) | -0.49 (0.17) | 0.044 |
|  | multimorbidity × US-born × pandemic | 0.53 (0.31-0.90) | -0.63 (0.19) | 0.03 |
|  | multimorbidity × CDAI × pre-pandemic | 0.73 (0.56-0.94) | -0.32 (0.09) | 0.026 |
|  | multimorbidity × pandemic | 0.53 (0.30-0.94) | -0.64 (0.21) | 0.036 |

Model was adjusted for age, sex, and race. Reference groups: pre-pandemic period, less than college, never married, men, never-smoker, US-born, low income, not employed, no/minimal depression, no health insurance, low active, 20-39 years old, non-obese, non-hypertension, and <6 hours sleep. CDAI: composite dietary antioxidant index; DII: dietary inflammatory index; ROR, ratio of odds ratios; T2D, type 2 diabetes.

**Table S7. Alcohol consumption and smoking patterns by depressive symptoms in prediabetes and type 2 diabetes**

| Variables | Prediabetes (n = 736) | | T2D (n = 441) | | P-value |
| --- | --- | --- | --- | --- | --- |
|  | 2017-2020 (n = 422) | 2021-2023 (n = 314) | 2017-2020 (n = 283) | 2021-2023 (n = 158) |  |
| Alcohol consumption, g/week | 16.1(4.8,97.7) | 16.1(2.4,55.8) | 4.8(1.2, 8.1) | 8.1(1.2,27.9) | < 0.0001 |
| Smoking status |  |  |  |  | < 0.0001 |
| Never | 212(44.0) | 173(57.6) | 151(46.5) | 64(40.9) |  |
| Ex-smoker | 107(28.4) | 89(26.3) | 87(39.1) | 62(37.4) |  |
| Current smoker | 103(27.6) | 52(16.1) | 45(14.4) | 32(21.7) |  |

Data are presented as weighted medians (interquartile ranges) for continuous variables and percentages for categorical variables. T2D, type 2 diabetes.
